# A Halogen-Atom Transfer (XAT)-Based Approach to Indole Synthesis Using Aryl Diazonium Salts and Alkyl Iodides

**DOI:** 10.1021/acs.orglett.2c02840

**Published:** 2022-10-21

**Authors:** Sebastian Govaerts, Kento Nakamura, Timothée Constantin, Daniele Leonori

**Affiliations:** † Department of Chemistry, University of Manchester, Oxford Road, Manchester M13 9PL, U.K.; ‡ Institute of Organic Chemistry, RWTH Aachen University, Landoltweg 1, Aachen 52056, Germany

## Abstract

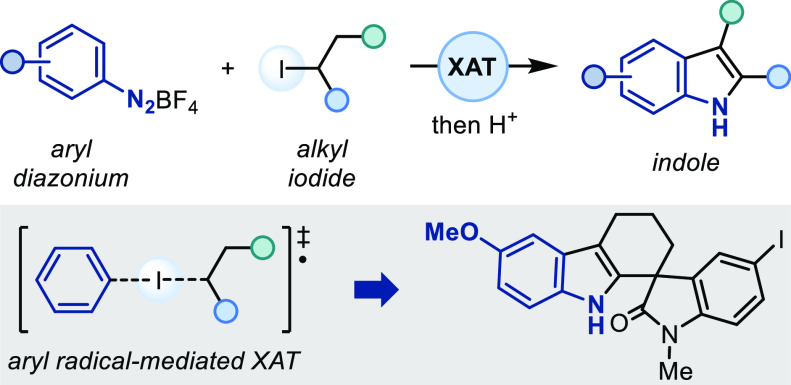

Indoles are among the most important *N*-heterocycles in pharmaceuticals. Here, we present an alternative to the classic Fischer indole synthesis based on the radical coupling between aryl diazoniums and alkyl iodides. This iron-mediated strategy features a double role for the aryl diazoniums that sequentially activate the alkyl iodides through halogen-atom transfer and then serve as radical acceptors. The process operates under mild conditions and enables the preparation of densely functionalized indoles.

The indole moiety is one of the most prevalent *N*-heterocycles in the structure of natural products and pharmaceuticals (Scheme 1A).^1^ The value of this motif can be realized by considering that in 2020, the indole-containing blockbuster drugs osimertinib, tezacaftor, and alectinib accounted together for more than $9 billion in sales.^2^

**Scheme 1:**
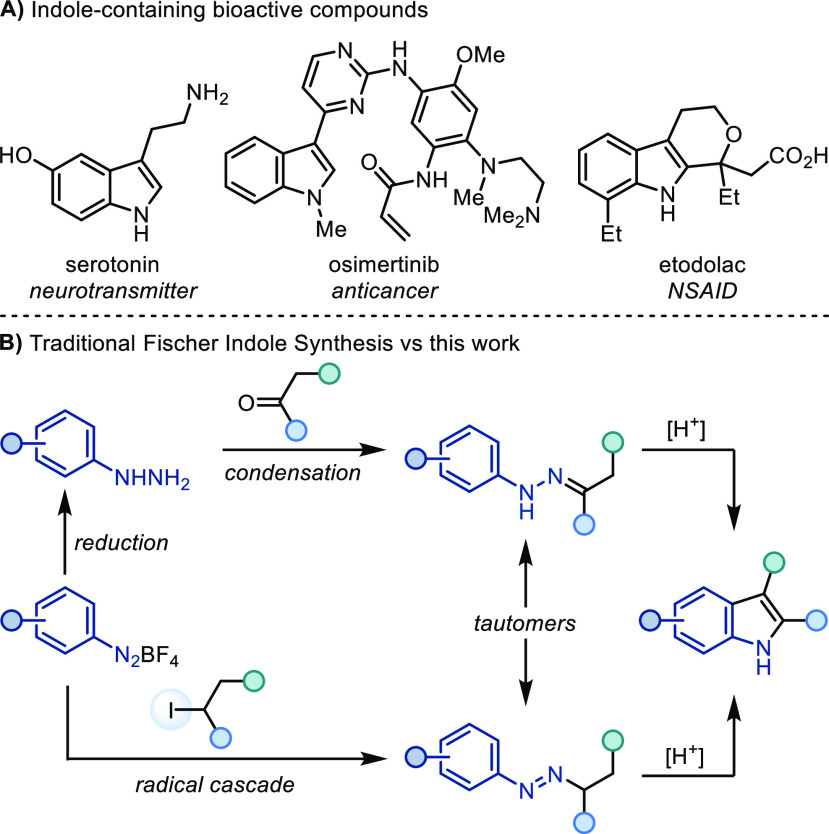
(A) Indole-Containing Bioactive Compounds and (B) Traditional Fischer Indole Synthesis versus This Work

Among the numerous methods for accessing indoles,^3^ the Fischer indole synthesis (FIS) introduced by Fischer in 1883^4,5^ continues to be commonly employed, with applications spanning small-scale drug discovery programs to large-scale manufacturing processes.^6,7^ In a typical FIS, an aryl hydrazine is condensed with a carbonyl species (ketone or aldehyde) to provide the corresponding hydrazone that cyclizes in the presence of a Brønsted or Lewis acid at, generally, high temperatures (Scheme 1B).

Despite its importance, the FIS suffers from several drawbacks, mainly associated with aryl hydrazines and harsh and/or anhydrous conditions usually needed for the condensation step. This sometimes results in a diminished tolerance of acid sensitive functional groups especially at increased temperatures. Furthermore, aryl hydrazines are often toxic^8^ and also of limited commercial availability. Current routes for their preparation require either a two-step synthesis from aniline (diazotization followed by reduction) or a transition metal-catalyzed cross-coupling reaction.^9^

Due to the relevance of indoles in the discovery, evolution, and manufacture of high-value pharmaceuticals, the identification of novel strategies for their assembly continues to remain a relevant goal. Herein, we discuss the development of an Fe(II)-mediated strategy for indole construction that directly engages aryl diazonium salts and alkyl iodides as coupling partners.

This novel disconnection is based on a radical azo coupling (Scheme 1B) and provides an alternative disconnection to the FIS by using readily available alkyl iodides and diazonium salts as building blocks under mild conditions. The key radical C–N bond formation step occurs at room temperature in the absence of acid and carbonyl electrophiles, which allows circumvention of some of the limitations associated with condensation between carbonyls and hydrazines. Although diazonium salts can be unstable and must be handled with care (see the Supporting Information), the use of BF_4_^–^ as a non-nucleophilic counteranion helps to mitigate safety risks.^10,11^

Building on the work of Citterio and Minisci,^12,13^ we envisioned a strategy in which SET (single-electron transfer) reduction of aryl diazonium salt (**A**) would deliver the corresponding aryl radical (**B**), which can in turn engage the alkyl iodide (**C**) in a XAT step (via collinear transition state **TS1**) (Scheme 2A). This would generate the corresponding alkyl radical (**E**) that could add across the N≡N bond of another molecule of diazonium (**A**), thus yielding a transient azo radical cation (**F**). This species should be easy to reduce to the corresponding *N*-alkyl-*N*′-aryl azo (**G**),^14−16^ which is a tautomeric form of the aryl hydrazone and would undergo acid-mediated indolization.

**Scheme 2:**
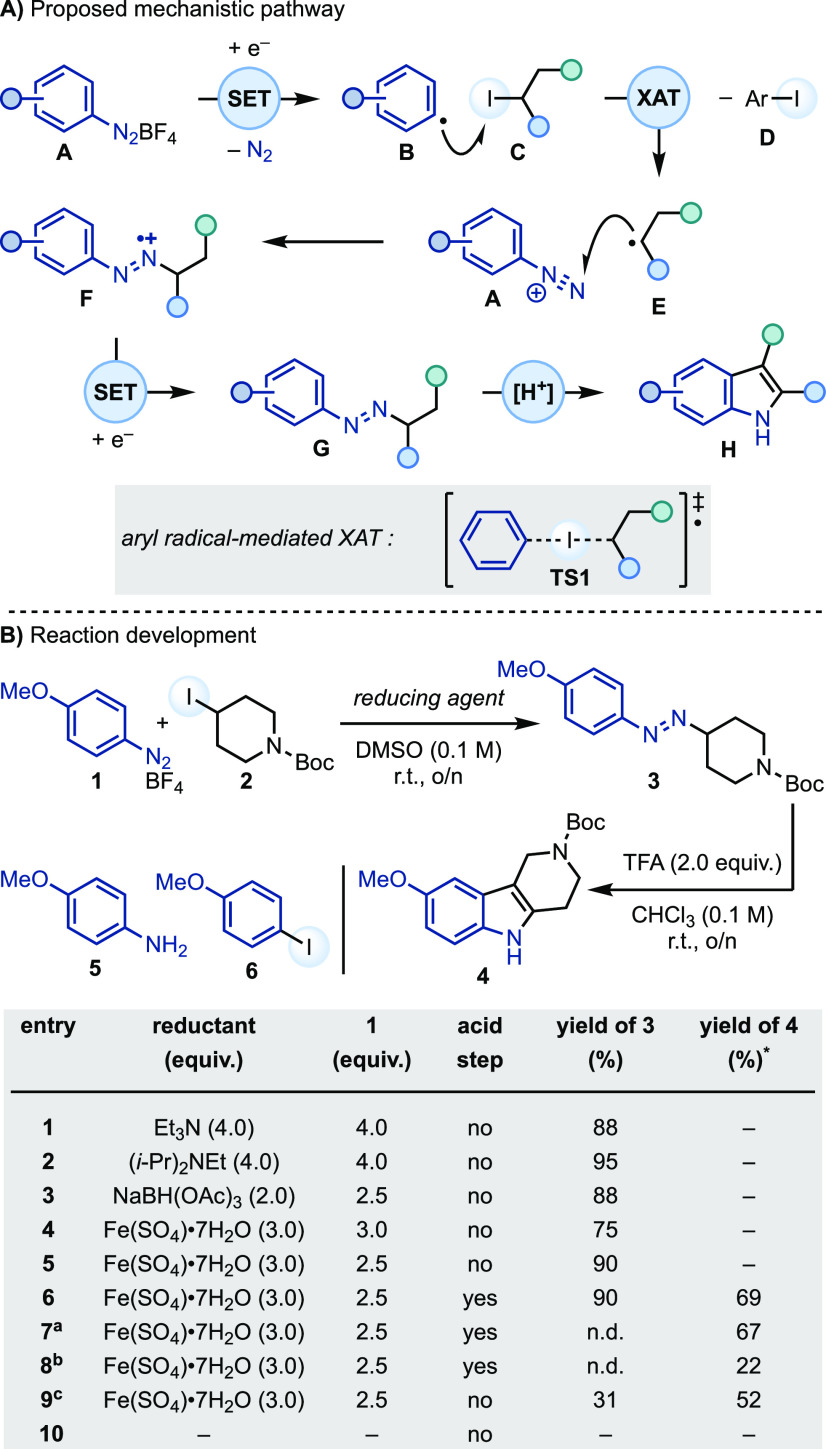
Proposed Reaction and its Optimization Using **1** and **2** Yield over two steps. Yield over two steps starting from **1**. Yield over three steps starting from **5**. Reaction run under open air.

We evaluated the implementation of this mechanistic proposal by studying the model reaction between diazonium **1** and alkyl iodide **2** to access piperidine-annellated indole **4** (via azo **3**) (Scheme 2B). DMSO was found to be the best solvent, allowing the reaction to be run at rt. We initially evaluated an array of bases with the aim of triggering a Gomberg–Bachmann type mechanism.^17^ Et_3_N and (*i*-Pr)_2_NEt were found to be suitable promoters for the formation of **3** (entries 1 and 2).^18^ However, the resulting reaction mixtures contained numerous byproducts, probably arising from the base-induced decomposition of **1**. This required a sizable excess of **1** and ultimately did not allow a smooth indolization process. We then decided to evaluate reductants with the aim of synergistically decreasing the amounts of **1** as well as the formation of unwanted side products. While the use of NaBH(OAc)_3_ was found to be successful (entry 3), we eventually identified the use of 3.0 equiv of cost-effective and benign Fe(SO_4_)·7H_2_O to be optimal (entry 4). Under these conditions, the concentration of **1** was decreased to 2.5 equiv and **3** was obtained in a high 90% yield (entry 5). Pleasingly, this azo product could be successfully converted into the corresponding indole **4** upon simple treatment with TFA (2.0 equiv) in CHCl_3_ at room temperature (entry 6). This stepwise approach could be telescoped in a single operation starting either from aryl diazonium **1** [two sequential steps (radical addition and indolization), entry 7] or aniline **5** [three sequential steps (diazotization, radical addition, and indolization), entry 8], albeit in lower yields.

To gain some insights supporting our proposed mechanism, we performed several control experiments. First, analysis of all of the successful reactions revealed the formation of 4-iodoanisole **6**, which is indicative of XAT taking place during the process.^19^ Second, while this reactivity is not particularly sensitive to air (entry 9), no formation of **3** or **6** was observed when [Fe(II)] was omitted, further highlighting its role in the activation of the diazonium species. Furthermore, addition of K_4_[Fe(II)(CN)_6_]·3H_2_O to the crude reaction mixture was used to confirm the presence of [Fe(III)] species via the formation of an intense Prussian blue color.^20^ Additionally, upon workup with NH_4_Cl, HRMS analysis revealed the presence of [Fe(III)Cl_4_]^−^ in the aqueous layer, confirming the single-electron oxidation of [Fe(II)] as part of the radical process. Combined, these evidences support our mechanistic proposal.

With a set of optimal reaction conditions in hand, we evaluated the scope of the process (Scheme 3). We initially focused on varying the nature of the alkyl iodide using commercially available **1** as the diazonium coupling partner, leading to valuable C5-substituted indoles (the most commonly encountered aromatic substitution pattern in indole drugs).^21^ Primary alkyl iodides (e.g., phthalimide **7**) were found to be compatible in the process, affording protected 5-MeO-tryptamine **23**, albeit in lower yield, owing to the more challenging XAT activation^19^ and indolization steps. Tryptamines with C5 substitution such as **23** are known for their biological activity^22,23^ encountered, for example, in the triptan migraine drugs,^24^ while oxygenated derivatives include the sleeping hormone melatonin^25^ and the natural product bufotenin.^26^ Secondary acyclic iodides (**8** and **9**) performed well, affording the corresponding indoles (**24** and **25**) in good yields. We then turned our attention to the engagement of cyclic alkyl iodides, which are high-value fragments in medicinal chemistry programs. Pleasingly, a series of C4-substituted cyclohexyl iodides (**10–15**) were found to be compatible (**26–31**). Six-membered heterocyclic derivatives (**16** and **17**) were also engaged successfully, leading to the assembly of **32** and **33** in moderate to good yields. Larger cyclic systems were evaluated next, and we successfully engaged a C3-iodo-azepane (**18**) in the process, thus giving tricyclic azepino[4,5-*b*]indole **34**. N-Methylated analogues of **34**, which can be obtained in one step by LiAlH_4_ reduction of the Boc functionality, are currently under investigation because of their therapeutic potential as nonhallucinogenic antidepressant alternatives to the alkaloid ibogaine.^27^ Thus far, this evaluation of scope demonstrated the process tolerates several important functionalities often encountered in organic synthesis like thioether (**32**), acetal (**31**), esters, and *N*-Boc-protected amines (**4**, **30**, **33**, **34**, **37**, and **38**). The latter are notable for being incompatible with the classical FIS, because they undergo acidic hydrolysis as a result of the hydrazone condensation step, and their manipulation requires additional steps following assembly of the core indole structure.^28,29^ The reaction could be scaled up without any erosion in yield as exemplified by the 1.0 mmol scale synthesis of **43**.

**Scheme 3:**
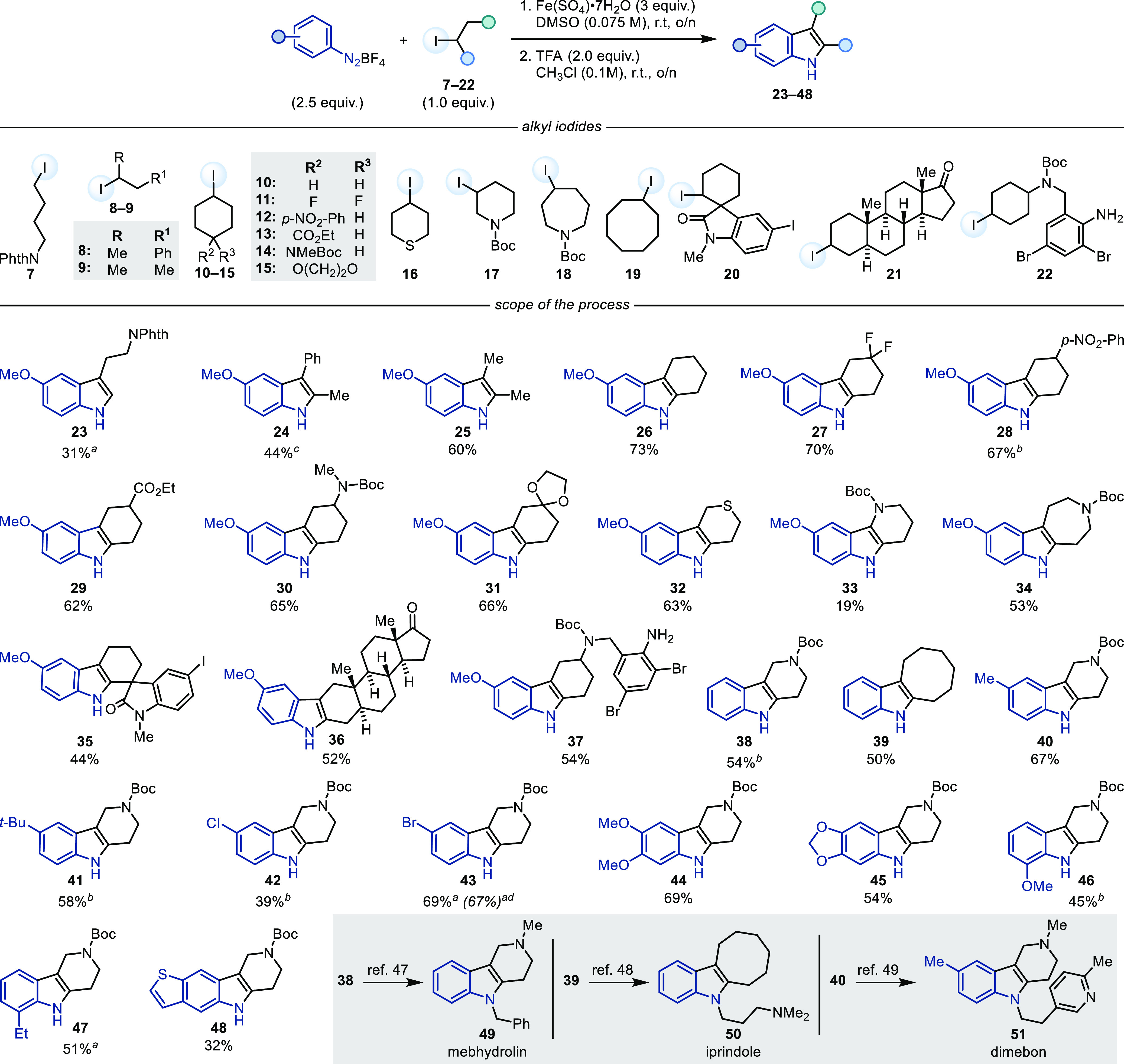
Substrate Scope Cyclization run at 50 °C. Cyclization conditions: l-tartaric acid (6.7 equiv), *s*-BuOH (1 M), 90 °C, 1–6 h. Cyclization conditions: TsOH·H_2_O (1.0 equiv), CH_3_CN (0.5 M), rt, 1 h. Reaction run on a 1.0 mmol scale.

It is pertinent to point out that aryl radicals are often used as competent H-atom transfer reagents targeting hydridic and enthalpically activated positions.^30,31^ It is therefore worth noting the compatibility of iodides **14** and **16**–**18** [hydridic α-heteroatom C(*sp*^*3*^)–H bonds], as well as **9**, and **12** [weak benzylic C(*sp*^*3*^)–H bonds], which could potentially give rise to HAT side processes.

The retrosynthetic advantage that this novel disconnection might bring to the assembly of complex indoles was demonstrated by using complex alkyl iodides. **20** can be conveniently prepared by iodo-amidation and gave spirocyclic indole **35** in good yield. This example also highlighted the strong chemoselectivity offered by the process that can discriminate between alkyl and aryl iodide, leading only to the XAT activation of the former. **21** and **22** can be obtained by Appel reaction on the neurosteroid androsterone and the cough suppressant ambroxol. These two derivatives gave indoles **36** and **37**, respectively, in useful yields, demonstrating tolerance of the ketone functionality (offering orthogonal selectivity to the FIS by leaving carbonyl groups untouched), as well as free aniline and aryl bromide, which are often employed in conjunction with an *ortho*-substitution pattern as handles for transition metal-catalyzed indole syntheses.^32^

Having benchmarked this reactivity on a diverse set of alkyl iodides, we evaluated its feasibility while modulating the substitution pattern of the diazonium salt using **2** as the alkyl iodide. Pleasingly, phenyl diazonium could be engaged successfully with both **2** as well as cyclooctyl iodide (**19**), thus giving indoles **37** and **38** in useful yields. These species are intermediates in the synthesis of antihistamine mebhydrolin **49**(33) and antidepressant iprindole **50**.^34^*para*-substituted derivatives with both weakly electron rich *t*-Bu and Me groups as well as electron-withdrawing Cl and Br atoms could be used in the chemistry, leading to functionalized indoles **40–43**. **40** is a direct precursor in the synthesis of antihistamine drug dimebon **51** [requiring only two steps (LiAlH_4_ reduction and *N*-alkylation)].^35^ Highly oxygenated diazoniums incorporating the 3,4-dimethoxy and 3,4-methylenedioxy motifs, which are common pharmacophores because of their function as protected catechols,^36,37^ were also amenable to this reactivity (**44** and **45**), as well as *ortho*-substituted derivatives (**46** and **47**). While electron-poor diazoniums based on *N*-heterocycles (e.g., pyridine) could not be used in this transformation, we successfully engaged a benzothiophene diazonium that gave tetracyclic **48** in moderate yield.

In conclusion, we have reported a novel strategy for preparing highly functionalized indoles via an Fe(II)-mediated radical coupling of readily available aryl diazonium salts and alkyl iodides. This strategy, based on an SET/XAT interplay, allows access to aryl-alkyl azo compounds that undergo indolization under mild conditions and tolerate several functional groups otherwise incompatible with traditional methods. We hope that the broad availability of alkyl iodides and the mild conditions will make this process of interest to both academic and industrial end-users.

## Notes

The data underlying this study are available in the published article and its Supporting Information.

## Supporting Information Available

Additional optimization, experimental details and procedures, and characterization of new compounds (PDF)
