# Supplementary material for: A Halogen-Atom Transfer (XAT)-Based Approach to Indole Synthesis Using Aryl Diazonium Salts and Alkyl Iodides
Source: Org Lett. 2022 Oct 21;24(43):7883–7. doi: 10.1021/acs.orglett.2c02840 (PMC9641658; doi:10.1021/acs.orglett.2c02840)
Supplement: Supplementary file 1 — ol2c02840_si_001.pdf [file ol2c02840_si_001.pdf]

A Halogen-Atom Transfer (XAT)-Based Approach to Indole Synthesis Using Aryl Diazonium Salts and Alkyl Iodides

Sebastian Govaerts[1†], Kento Nakamura[1†], Timothée Constantin[1]*, and Daniele Leonori[1,2]*

[1] *School of Chemistry, University of Manchester, Oxford Road, Manchester M13 9PL, UK*

[2] *Institute of Organic Chemistry, RWTH Aachen University, Landoltweg 1, 52056, Aachen, Germany*

[†] These authors contributed equally to this work.

*timothee.constantin@manchester.ac.uk

*daniele.leonori@rwth-aachen.de

Table of contents

1 General Experimental Details 2

2 Starting Material Synthesis 3

Synthesis of alkyl iodides 3

Synthesis of diazonium salts 8

3 Indole Synthesis via XAT 12

Reaction Optimization with DIPEA 12

Reaction Optimization with Different Reductants 13

Reaction Optimization for the Indoles Synthesis From Alkyl Iodides 16

Scope of the Indole Synthesis From Alkyl Iodides 18

Procedure for 1 mmol scale reaction 30

4 Mechanistic Studies 32

HRMS Detection of Fe(III) 32

Prussian Blue Test 32

5 NMR Spectra 34

6 References 59

1 General Experimental Details

All required fine chemicals were used directly without purification unless stated otherwise. All air and moisture sensitive reactions were carried out under nitrogen atmosphere using standard Schlenk manifold techniques. All solvents were bought from Acros as 99.8% purity. ^{1}H and ^{13}C Nuclear Magnetic Resonance (NMR) spectra were acquired at various field strengths as indicated and were referenced to $CHCl_3$ (7.26 and 77.0 ppm for ^{1}H and ^{13}C respectively). ^{1}H NMR coupling constants are reported in Hertz and refer to apparent multiplicities and not true coupling constants. Data are reported as follows: chemical shift, integration, multiplicity (s = singlet, br s = broad singlet, d = doublet, t = triplet, q = quartet, p = pentet, sxt = sextet, hept = heptet, m = multiplet, dd = doublet of doublets, etc.), proton assignment (determined by 2D NMR experiments: COSY, HSQC and HMBC) where possible. High-resolution mass spectra were obtained using a Thermo Q Exactive hybrid quadrupole-orbitrap mass spectrometer. or a Fissions VG Trio 2000 quadrupole mass spectrometer. Spectra were obtained using electron impact ionization (EI) and chemical ionization (CI) techniques, or positive electrospray (ES). Analytical TLC: aluminum backed plates pre-coated (0.25 mm) with Merck Silica Gel 60 F_{254}. Compounds were visualized by exposure to UV-light or by dipping the plates in permanganate ($KMnO_4$), phosphomolybdic acid (PMA) or ninhydrin stain followed by heating. Flash column chromatography was performed using Merck Silica Gel 60 (40–63 μm). All mixed solvent eluents are reported as v/v solutions. All the reactions were conducted in CEM 10 mL glass microwave tubes. When needed, reactions were heated up using an oil bath or an aluminum block.

2 Starting Material Synthesis

2.1 Synthesis of alkyl iodides

***tert*-Butyl (2-Amino-3,5-Dibromobenzyl)(4-hydroxycyclohexyl)carbamate (S1)**

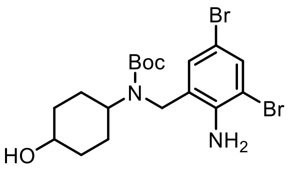

An oven-dried round-bottom flask was charged with a stirring bar, ambroxol hydrochloride (1.24 g, 3.0 mmol, 1.0 equiv.) and placed under an atmosphere of N_2. Anhydrous THF (30 mL, 0.1 M) was added, the mixture was cooled to 0 ℃ followed by addition of Et_3N (840 mL, 6.0 mmol, 2.0 equiv.). After stirring for 5 minutes, a solution of Boc_2O (720 mg, 3.3 mmol, 1.1 equiv.) in anhydrous THF (10 mL, 0.33 M) was added over the course of 5 minutes. The reaction mixture was allowed to warm to room temperature overnight, diluted with water (30 mL), extracted with Et_2O (20 mL x2) and EtOAc (20 mL). The combined organic layers were dried over $MgSO_4$, filtered and evaporated. The crude product was purified by flash column chromatography on silica gel eluting petrol–EtOAc to give **S1** as a solid (1.28 g, 89%). ^{1}H NMR (400 MHz, $CDCl_3$) δ 7.47 (1H, d, J = 3.0 Hz), 7.08 (1H, d, J = 3.0 Hz), 4.85 (2H, br s), 4.28 (2H, s), 3.52–3.50 (2H, m), 1.68–1.56 (5H, m), 1.46 (9H, s), 1.32–1.22 (2H, m); ^{13}C NMR (101 MHz, $CDCl_3$) δ 156.2, 142.2, 133.8, 131.8, 124.8, 110.1, 108.0, 81.0, 70.0, 56.6, 46.8, 35.0, 28.5, 28.2; HRMS (ESI$^+$): m/z calcd. for $[M+Na]^+$ $[C_{18}H_{26}Br_2N_2O_3Na]^+$ 501.0182, found 501.0180.

2,5'-Diiodo-1'-methylspiro[cyclohexane-1,3'-indolin]-2'-one (20)

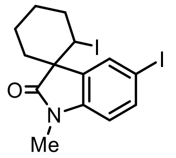

20 was prepared according to a literature procedure[1]: A round-bottom flask was charged with a stirring bar and *N*-methyl-*N*-phenylcyclohex-1-ene-1-carboxamide (465 mg, 2.16 mmol, 1.0 equiv.). MeCN (21.6 mL, 0.1 M) was added, followed by $PhI(OAc)_2$ (1.04 g, 3.24 mmol, 1.5 equiv.) and I_2 (822 mg, 3.24 mmol, 1.5 equiv.). The solution was stirred for 3 h at room temperature, quenched with $Na_2S_2O_3$ sat. (10 mL) and extracted with EtOAc (20 mL ×3). The combined organic layers were dried over $MgSO_4$, filtered and evaporated. The crude mixture was purified by flash column chromatography on silica gel eluting petrol–EtOAc to give **20** as a solid (101 mg, 10%). ^{1}H NMR (400 MHz, $CDCl_3$) δ 7.62 (1H, dd, J = 8.0, 1.0 Hz), 7.38 (1H,

d, J = 1.0 Hz), 6.60 (1H, d, J = 8.5 Hz), 4.26 (1H, dd, J = 13.0, 4.5 Hz), 3.19 (3H, s), 2.99 (1H, dq, J = 13.0, 4.5 Hz), 2.37–2.24 (2H, m), 1.95–1.91 (1H, m), 1.83–1.75 (2H, m), 1.71–1.68 (1H, m), 1.53–1.44 (1H, m). Data in accordance with the literature. [1]

General procedure for the preparation of alkyl iodides (Appel reaction) – GP1

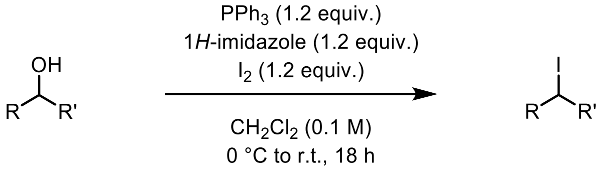

To a stirred solution of the alcohol (1.0 equiv.) in CH_2Cl_2 (0.1 M) were added PPh_3 (1.2 equiv.), imidazole (1.2 equiv.) and I_2 (1.2 equiv.) under N_2 atmosphere at 0 °C. The reaction was stirred for 16 h at room temperature and then diluted with H_2O (30 mL). The mixture was extracted with CH_2Cl_2 (30 mL ×3). The combined organic layers were washed with $Na_2S_2O_3$ sat., (30 mL), brine (30 mL), dried over $MgSO_4$, filtered and evaporated. Purification by flash column chromatography eluting petrol–EtOAc on silica gel gave the products.

(2-Iodopropyl)benzene (8)

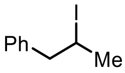

Following **GP1**, 1-phenylpropan-2-ol (1.36 g, 10.0 mmol) gave (2-iodopropyl)benzene **8** as an oil (1.19 g, 48%). ^{1}H NMR (400 MHz, $CDCl_3$) δ 7.32–7.24 (3H, m), 7.17 (2H, d, J = 6.8 Hz), 4.33 (1H, app sxt, J = 6.8 Hz), 3.31–3.26 (1H, m), 3.08–3.03 (1H, m), 1.89 (3H, d, J = 6.8 Hz). Data in accordance with the literature. [8]

1,1-Difluoro-4-iodocyclohexane (11)

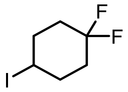

Following **GP1**, 4,4-difluorocyclohexan-1-ol (0.68 g, 5.0 mmol) gave 1,1-difluoro-4-iodocyclohexane **11** as an oil (0.33 g, 27%). ^{1}H NMR (400 MHz, $CDCl_3$) δ 4.52–4.41 (1H, m), 2.21–2.09 (4H, m), 2.08–2.00 (2H, m), 1.99–1.84 (2H, m). Data in accordance with the literature. [9]

1-(4-Iodocyclohexyl)-4-nitrobenzene (12)

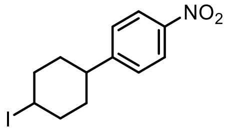

Step i: A 25 mL oven-dried round-bottom flask was charged with a stirring bar and 4-(4-nitrophenyl)cyclohexan-1-one (509 mg, 2.32 mmol, 1.0 equiv.). MeOH (4.6 mL, 0.5 M) was added, the mixture was cooled to 0 °C followed by addition of $NaBH_4$ (105 mg, 2.78 mmol, 1.2 equiv.). The mixture was allowed to warm up to room temperature over the course of 3 h, water (10 mL) was added and the mixture was extracted with CH_2Cl_2 (20 mL x3). The combined organic layers were dried over $MgSO_4$, filtered and evaporated to give crude 4-(4-nitrophenyl)cyclohexan-1-ol which was used directly in the next step without further purification.

Step ii: Following **GP1** but using PPh_3 (854 mg, 3.48 mmol, 1.5 equiv.), imidazole (245 mg, 3.60 mmol, 1.55 equiv.), I_2 (883 mg, 3.25 mmol, 1.4 equiv.) and crude 4-(4-nitrophenyl)cyclohexan-1-ol gave 1-(4-iodocyclohexyl)-4-nitrobenzene **12** as a solid (291 mg, 38% yield over 2 steps). ^{1}H NMR (400 MHz, $CDCl_3$) δ 8.13 (2H, d, *J* = 8.8 Hz), 7.40 (2H, d, *J* = 8.8 Hz), 4.95–4.86 (1H, m), 2.69 (1H, tt, *J* = 12.2, 3.5 Hz), 2.27–2.12 (2H, m), 2.11–1.89 (2H, m), 1.85–1.52 (4H, m); ^{13}C NMR (101 MHz, $CDCl_3$) δ 154.2, 146.4, 128.0, 123.7, 43.7, 36.3, 35.1, 29.8; HRMS (ASAP): m/z calcd. for $[M+H]^+$ $[C_{12}H_{15}INO_2]^+$ 332.0142, found 332.0143.

Ethyl 4-Iodocyclohexanecarboxylate (13)

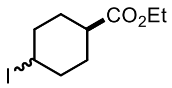

Following **GP1**, ethyl 4-hydroxycyclohexane carboxylate (517 mg, 3.00 mmol) gave ethyl 4-iodocyclohexane carboxylate **13** as an oil (447 mg, 53%, *cis*/*trans* 5.7:1). ^{1}H NMR (400 MHz, $CDCl_3$, major *cis* isomer) δ 4.67–4.64 (1H, m), 4.16 (2H, q, *J* = 7.2 Hz) 2.43–2.37 (1H, m), 2.17–2.09 (2H, m), 2.02–1.95 (2H, m), 1.83–1.72 (3H, m), 1.27 (3H, t, *J* = 7.2 Hz). Data in accordance with the literature. [13]

***tert*-Butyl *N*-(4-Iodocyclohexyl)-*N*-methylcarbamate (14)**

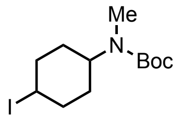

Following **GP1**, *tert*-butyl *N*-(*trans*-4-hydroxycyclohexyl)-*N*-methylcarbamate (645 mg, 2.81 mmol) gave *tert*-butyl *N*-(4-iodocyclohexyl)-*N*-methylcarbamate **14** as a solid (237 mg, 70%, *cis*/*trans* 4:1). ^{1}H NMR (400 MHz, $CDCl_3$, major *cis* isomer) δ 4.80–4.79 (1H, m), 4.04 (1H, br s), 2.81 (3H, s), 2.15–2.11 (2H, m), 2.05–1.96 (2H, m), 1.67–1.55 (4H, m), 1.46 (9H, s). Data in accordance with the literature. [10]

8-Iodo-1,4-dioxaspiro[4.5]decane (15)

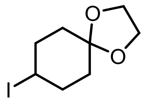

Following **GP1**, 1,4-dioxaspiro[4.5]decan-8-ol (0.79 g, 5.0 mmol) gave 8-iodo-1,4-dioxaspiro[4.5]decane **15** as an oil (0.99 g, 74%). ^{1}H NMR (400 MHz, $CDCl_3$) δ 4.49–4.32 (1H, m), 4.01–3.83 (4H, m), 2.15–2.02 (4H, m), 1.80 (2H, ddd, *J* = 12.4, 7.7, 4.9 Hz), 1.60 (2H, ddd, *J* = 13.1, 8.3, 4.6 Hz). Data in accordance with the literature. [11]

4-Iodotetrahydro-2*H*-thiopyran (16)

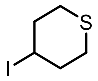

Following **GP1**, tetrahydrothiopyran-4-ol (0.96 g, 8.2 mmol) gave 4-iodotetrahydro-2*H*-thiopyran **16** as an oil (1.41 g, 75%). ^{1}H NMR (400 MHz, $CDCl_3$) δ 4.55–4.40 (1H, m), 2.90–2.74 (2H, m), 2.65–2.45 (2H, m), 2.40–2.15 (4H, m). Data in accordance with the literature. [11]

***tert*-Butyl 4-Iodoazepane-1-carboxylate (18)**

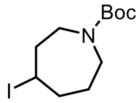

Following **GP1**, *tert*-butyl 4-hydroxyazepane-1-carboxylate (500 mg, 2.32 mmol) gave *tert*-butyl 4-iodoazepane-1-carboxylate **18** as an oil (547 mg, 72%). ^{1}H NMR (500 MHz, $CDCl_3$, rotamers) δ 4.48 (1H, app br s), 3.49–3.38 (2H, m), 3.36–3.26 (2H, m), 2.24 (2H, app br s), 2.15–2.04 (2H, m), 1.84 (1H, app br s), 1.76–1.68 (1H, m), 1.46 (9H, s). Data in accordance with the literature. [12]

Iodocyclooctane (19)

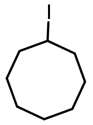

Following **GP1**, cyclooctanol (1.98 mL, 10.0 mmol) gave iodocyclooctane **19** as an oil (2.34 g, 95%). ^{1}H NMR (500 MHz, $CDCl_3$, rotamers) δ 4.53–4.42 (1H, m), 3.52–3.39 (2H, m), 3.37–3.24 (2H, m), 2.30–2.19 (2H, m), 2.14–2.02 (1H, m), 1.77–1.69 (1H, m), 1.46 & 1.46 (9H, s). Data in accordance with the literature.[8]

(5*S*,8*R*,9*S*,10*S*,13*S*,14*S*)-3-Iodo-10,13-dimethylhexadecahydro-17*H*-cyclopenta[*a*]phenanthren-17-one (21)

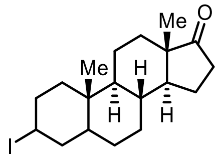

Following **GP1**, androsterone (553 mg, 3.00 mmol) gave **21** as a solid (880 mg, 95%) as a mixture of diastereoisomers. dr = 2.1:1. ^{1}H NMR (400 MHz, $CDCl_3$, major isomer) δ 4.93–4.87 (1H, m), 2.43 (1H, ddd, *J* = 19.2, 8.8, 1.1 Hz), 2.14–1.99 (1H, m), 1.99–1.86 (2H, m), 1.85–1.61 (7H, m), 1.61–1.39 (5H, m), 1.34–1.14 (5H, m), 1.07–1.04 (1H, m), 0.88 (3H, s), 0.85 (3H, s). Data in accordance with the literature.[14]

***tert*-Butyl (2-Amino-3,5-dibromobenzyl)(4-iodocyclohexyl)carbamate (22)**

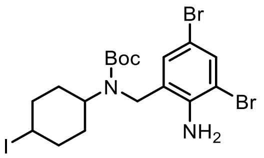

Following **GP1** but using PPh_3 (404 mg, 1.65 mmol, 1.5 equiv.), (116 mg, 1.71 mmol, 1.55 equiv.) and I_2 (419 mg, 1.54 mmol, 1.4 equiv.), *tert*-butyl (2-amino-3,5-dibromobenzyl)(*trans*-4-hydroxycyclohexyl)carbamate **S1** (522 mg, 1.1 mmol) gave **22** as a solid (233 mg, 35%, *cis*/*trans* 7.7:1). ^{1}H NMR (400 MHz, $CDCl_3$, major *cis* isomer) δ 7.53–7.48 (1H, m), 7.22–7.15 (1H, m), 5.56 (1H, br s), 5.29–4.84 (2H, m), 4.77–4.66 (1H, m), 4.37 (2H, s), 3.82–3.05 (1H, m), 2.20–1.95 (4H, m), 1.61–1.48 (13H, m); ^{13}C NMR (101 MHz, $CDCl_3$) δ 156.3, 142.5, 133.8, 132.3, 126.6, 125.6, 124.7, 109.9, 107.8, 81.0, 80.8, 56.7, 47.0, 36.3, 33.6, 28.7, 28.6, 26.7; HRMS (ESI$^+$): m/z calcd. for [M+Na]$^+$ $[C_{18}H_{25}Br_2IN_2O_2Na]^+$ 610.9205, found 610.9208.

2.2 Synthesis of diazonium salts

General procedure for the preparation of aryl diazonium tetrafluoroborates – GP2

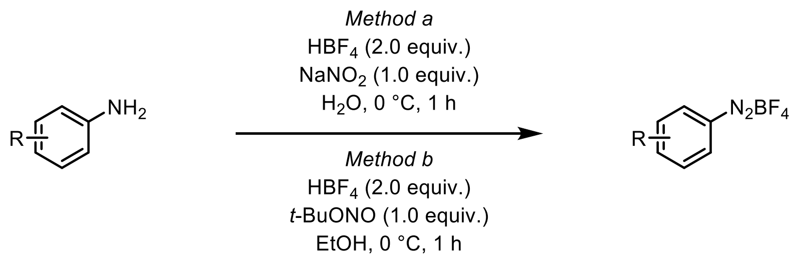

GP2a: The aniline (1.0 equiv.) was suspended in H_2O (2 M) in a large round-bottom flask. The suspension was cooled to 0 °C and treated with HBF_4 (48% in water, 2.0 equiv.) and stirred until homogeneous. An aqueous solution (10 M) of $NaNO_2$ (1.0 equiv.) was added slowly (*caution*: HONO is evolved) and the mixture was stirred for 1 h. The resulting solid was filtered off, thoroughly washed with ice-cold water and Et_2O. The crude product was recrystallized from acetone/Et_2O to give the pure product as a solid.

GP2b (for anilines poorly soluble in H_2O/HBF_4): The aniline was dissolved in EtOH (1.0 M) in a round-bottom flask equipped with a stirring bar at 0 °C. HBF_4 (48% in water, 2.0 equiv.) was slowly added, followed by dropwise addition of *t*-BuONO (1.0 equiv.). After completion of the addition, the reaction was stirred for 1 h. The resulting solid was filtered off, washed with ice-cold water and Et_2O. The crude product was recrystallized from acetone/Et_2O to yield the pure product as a solid.

All diazonium salts were used directly after filtration following recrystallisation. If necessary, the product was dried under a stream of N_2 gas but never under reduced pressure or by heating (see safety note below).

Safety note: Diazonium salts are inherently unstable molecules and decompose to release a molar equivalent of N_2 gas. While no incident has been observed in our laboratory, diazonium salts must be handled with great care. All diazonium salts were dried under atmospheric pressure at room temperature, under a flow of air or nitrogen, were manipulated with PTFE spatula, never exposed to metal surfaces, and stored in the freezer. We synthesized diazonium tetrafluoroborate salts to mitigate the explosion risk.

Note: Distilling aniline before use is recommended to increase the shelf-life and stability of the corresponding diazonium salts.

4-Methoxybenzenediazonium Tetrafluoroborate (1)

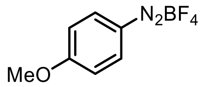

Following **GP2a**, *p*-anisidine (2.46 g, 20.0 mmol) gave 4-methoxybenzenediazonium tetrafluoroborate **1** as a solid (4.28 g, 97%). ^{1}H NMR (400 MHz, DMSO-d_6) δ 8.61 (2H, d, J = 9.4 Hz), 7.48 (2H, d, J = 9.4 Hz), 4.04 (3H, s). Data in accordance with the literature.[2]

Benzenediazonium Tetrafluoroborate (S2)

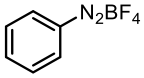

Following **GP2a**, aniline (0.91 mL, 10.0 mmol) gave benzenediazonium tetrafluoroborate **S2** as a solid (1.87g, 98%). ^{1}H NMR (400 MHz, DMSO-d_6) δ 8.73–8.59 (2H, m), 8.26 (1H, t, J = 7.7 Hz), 7.98 (2H, t, J = 8.2 Hz). Data in accordance with the literature.[3]

4-Methylbenzenediazonium Tetrafluoroborate (S3)

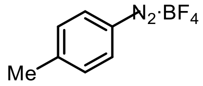

Following **GP2a**, *p*-toluidine (1.07 g, 10.0 mmol) gave 4-methylbenzenediazonium tetrafluoroborate **S3** as a solid (1.85 g, 90%). ^{1}H NMR (400 MHz, DMSO-d_6) δ 8.54 (2H, d, J = 8.6 Hz), 7.79 (2H, d, J = 8.6 Hz), 2.57 (3H, s). Data in accordance with the literature.[2]

4-(*tert*-Butyl)benzenediazonium Tetrafluoroborate (S4)

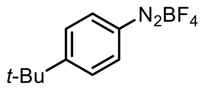

Following **GP2a**, 4-(*tert*-butyl)aniline (1.59 mL, 10.0 mmol) gave 4-(*tert*-butyl)benzenediazonium tetrafluoroborate **S4** as a solid (2.35 g, 95%). ^{1}H NMR (400 MHz, DMSO-d_6) δ 8.59 (2H, d, J = 9.0 Hz), 8.03 (2H, d, J = 9.0 Hz), 1.35 (9H, s). Data in accordance with the literature.[4]

4-Chlorobenzenediazonium Tetrafluoroborate (S5)

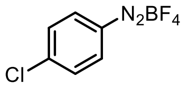

Following **GP2a**, 4-chloroaniline (1.28 g, 10.0 mmol) gave 4-chlorobenzenediazonium tetrafluoroborate **S5** as a solid (2.12 g, 94%). ^{1}H NMR (400 MHz, DMSO-d_6) δ 8.69 (2H, d, J = 9.0 Hz), 8.11 (2H, d, J = 8.8 Hz). Data in accordance with the literature. [2]

4-Bromobenzenediazonium Tetrafluoroborate (S6)

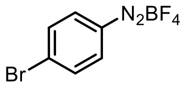

Following **GP2a**, 4-bromoaniline (1.72 mL, 15.0 mmol) gave 4-bromobenzenediazonium tetrafluoroborate **S6** as a solid (3.7 g, 90%). ^{1}H NMR (400 MHz, acetone-d_6) δ 8.78 (2H, d, J = 9.2 Hz), 8.32 (2H, d, J = 9.2 Hz). Data in accordance with the literature. [5]

3,4-Dimethoxybenzenediazonium Tetrafluoroborate (S7)

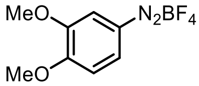

Following **GP2a**, 3,4-dimethoxyaniline (1.53 g, 10.0 mmol) gave 3,4-dimethoxybenzenediazonium tetrafluoroborate **S7** as a solid (1.45 g, 65%). ^{1}H NMR (400 MHz, DMSO-d_6) δ 8.44 (1H, dd, J = 9.1, 2.8 Hz), 8.18 (1H, d, J = 2.8 Hz), 7.52 (1H, d, J = 9.1 Hz), 4.05 (3H, s), 3.89 (3H, s). Data in accordance with the literature. [6]

Benzo[*d*][1,3]dioxole-5-diazonium Tetrafluoroborate (S8)

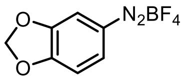

Following **GP2a**, benzo[*d*][1,3]dioxol-5-amine (1.37 g, 10.0 mmol, 1.0 equiv.) gave benzo[*d*][1,3]dioxole-5-diazonium tetrafluoroborate **S8** as a solid (1.92 g, 82%). ^{1}H NMR (400 MHz, DMSO-d_6) δ 8.42 (1H, dd, J = 8.8, 2.2 Hz), 8.05 (1H, d, J = 2.2 Hz), 7.49 (1H, d, J = 8.7 Hz), 6.45 (2H, s). Data in accordance with the literature. [6]

2-Methoxybenzenediazonium Tetrafluoroborate (S9)

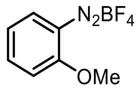

Following **GP2a**, *o*-anisidine (1.12 mL, 10.0 mmol) gave 2-methoxybenzenediazonium tetrafluoroborate **S9** as a solid (1.86 g, 84%). ^{1}H NMR (600 MHz, DMSO-d_6) δ 8.52 (1H, dd, *J* = 8.4, 1.4 Hz), 8.26–8.21 (1H, m), 7.69 (1H, d, *J* = 8.4 Hz), 7.47–7.42 (m, 1H), 4.20 (3H, s). Data in accordance with the literature. [4]

2-Ethylbenzenediazonium Tetrafluoroborate (S10)

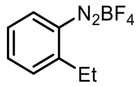

Following **GP2a**, 2-ethylaniline (1.23 mL, 10.0 mmol) gave 2-ethylbenzenediazonium tetrafluoroborate **S10** as a solid (78%, 1.72 g). ^{1}H NMR (400 MHz, acetone-d_6) δ 8.75 (1H, dd, *J* = 8.3, 1.5 Hz), 8.25 (1H, td, *J* = 8.2, 1.5 Hz), 7.94 (1H, dd, *J* = 8.2, 1.2 Hz), 7.85 (1H, td, *J* = 8.0, 1.2 Hz), 2.92 (2H, q, *J* = 4.8 Hz), 1.64 (3H, t, *J* = 4.8 Hz). Data in accordance with the literature. [7]

Benzo[*b*]thiophene-5-diazonium Tetrafluoroborate (S11)

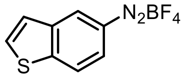

Following **GP2b**, benzo[*b*]thiophen-5-amine (299 mg, 2.0 mmol) gave benzo[*b*]thiophene-5-diazonium tetrafluoroborate **S11** as a solid (395 mg, 80%). ^{1}H NMR (400 MHz, DMSO-d_6) δ 9.28 (1H, d, *J* = 2.0 Hz), 8.65 (1H, d, *J* = 8.8 Hz), 8.45 (1H, dd, *J* = 8.8, 2.0 Hz), 8.30 (2H, d, *J* = 5.6 Hz), 7.91 (1H, dd, *J* = 5.6, 0.8 Hz); ^{13}C NMR (101 MHz, DMSO-d_6) δ 150.7, 139.1, 134.4, 129.8, 125.9, 124.7, 124.2, 110.2; HRMS (ESI^{+}): m/z calcd. for $[M]^{+}$ $[C_8H_5N_2S]^{+}$ 161.0168, found 161.0160..

3 Indole Synthesis via XAT

3.1 Reaction Optimization with DIPEA

General Procedure for the Reaction Optimization with DIPEA (Diazene Formation) – GP3

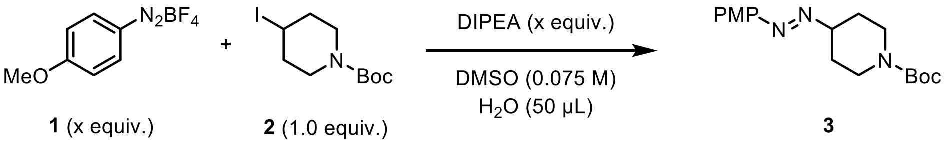

A dry tube equipped with a stirring bar was charged with *tert*-butyl 4-iodopiperidine-1-carboxylate **2** (0.10 mmol, 1.0 equiv.) and 4-methoxybenzenediazonium tetrafluoroborate **1**. The tube was capped with a Supelco aluminium crimp seal with septum (PTFE/butyl), evacuated and refilled with N_2 (x 3). Degassed solvent (1.5 mL, 0.075 M) and H_2O (50 µL) was added. DIPEA was then added dropwise. The reaction was stirred at room temperature in the dark for 16 hours. NH_4Cl sat. (3 mL) and EtOAc (3 mL) were added and the mixture was shaken vigorously. 1,3-Dinitrobenzene (17 mg, 0.1 mmol, 1.0 equiv.) was added and the layers were separated. The aqueous layer was extracted with EtOAc, the combined organic layers were dried ($MgSO_4$), filtered and evaporated. $CDCl_3$ (0.5 mL) was added and the mixture was analysed by ^1H NMR spectroscopy.

Table S1 – Optimization With DIPEA

Entry	Diazonium 1 (equiv.)	DIPEA (equiv.)	Yield of 3 (%)
**1**	2	1.5	54
**2**	3	1.5	56
**3**	4	1.5	59
**4**	4.5	1.5	58
**5**	4	1.2	48
**6**	4	2	71
**7**	4	2.5	80
**8**	4	3	93
**9**	4	4	95

In spite of the excellent NMR yields obtained (Table S1), this methodology suffered from difficult separation, probably because of the organic by-products arising from a Gomberg-

Bachmann mechanism and/or from a HAT/XAT sequence involving the amine. We therefore turned our attention towards the use of other reductants.

3.2 Reaction Optimization with Different Reductants

General Procedure for the Reaction Optimization with Different Reductants (Diazene Formation) – GP4

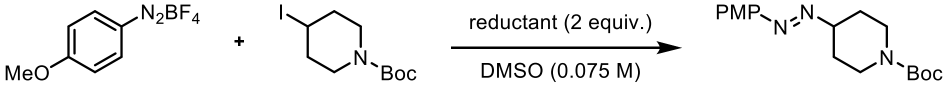

1 (2.0 equiv.) **2** (1.0 equiv.) **3**

A dry tube equipped with a stirring bar was charged with *tert*-butyl 4-iodopiperidine-1-carboxylate **2** (0.10 mmol, 1.0 equiv.), the reductant (0.20 mmol, 2.0 equiv.) and 4-methoxybenzenediazonium tetrafluoroborate **1** (44.1 mg, 0.2 mmol, 2.0 equiv.). The tube was capped with a Supelco aluminium crimp seal with septum (PTFE/butyl), evacuated and refilled with N_2 (x 3). Degassed solvent (1.5 mL, 0.075 M) was added at once (vigorous gas evolution was observed in certain cases). The reaction was stirred at room temperature in the dark for 16 hours. NH_4Cl sat. (3 mL) and EtOAc (3 mL) were added and the mixture was shaken vigorously. 1,3-Dinitrobenzene (17 mg, 0.1 mmol, 1.0 equiv.) was added and the layers were separated. The aqueous layer was extracted with EtOAc, the combined organic layers were dried ($MgSO_4$), filtered and evaporated. $CDCl_3$ (0.5 mL) was added and the mixture was analysed by ^{1}H NMR spectroscopy.

Table S2 – Reductant Screening

Entry	Reductant	Yield of 3 (%)
**1**	Mg	22
**2**	Zn	25
**3**	Cu powder	56
**4**	Cu wire	33
**5**	CuI	24
**6**	CuCN	25
**7**	Mn	50
**8**	Rongalite	0
**9**	$NaBH_3CN$	7
**10**	$NaBH(OAc)_3$	65

**11**	$FeSO_4 \cdot 7H_2O$	71
**12**	$FeClO_4 \cdot H_2O$	43
**13**	1p coin	37

All the reductants screened gave the desired product (Table S2), with the exception of rongalite. Among them, $NaBH(OAc)_3$ (Table S2, entry 10) and $FeSO_4 \cdot 7H_2O$ (Table S2, entry 11) were the most efficient ones. A screening of different Fe(II) salts proved $FeSO_4 \cdot 7H_2O$ to be the best candidate (not shown here). We decided to carry out the solvent screening with those two reductants (Table S3).

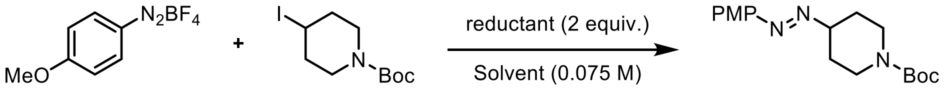

1 (2.0 equiv.) **2** (1.0 equiv.) **3**

Table S3 – Solvent screening

Entry	Solvent	Yield 3 (STAB as red, %)	Yield 3 ([Fe] as red, %)
**1**	THF	20	traces
**2**	EtOH	32	traces
**3**	EtOAc	traces	traces
**4**	Acetone	25	traces
**5**	DMF	30	43
**6**	DMSO	65	71

DMSO was found to be the most suitable solvent (Table S3, entry 6), while $FeSO_4 \cdot 7H_2O$ was found to slightly outperform $NaBH(OAc)_3$ (STAB) as reductant. We anticipated it to result in a greater chemoselectivity than the borohydride, which could potentially react with carbonyl groups. $FeSO_4 \cdot 7H_2O$ was therefore chosen as reductant and used to carry out a screening of the reaction stoichiometry (Table S4). The results show that the highest yields of azo compound**3** (90%) were obtained when a slight excess of diazonium **1** (2.5 equiv.) was used in combination with 3.0 equiv. of $FeSO_4 \cdot 7H_2O$ as reductant (Table S4, entry 12) while keeping alkyl iodide **2** as the limiting reagent.

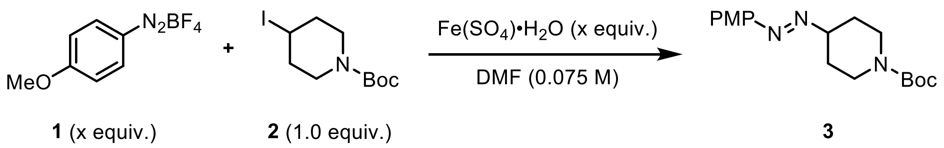

Table S4 – Effect of the Stoichiometry

Entry	1 (equiv.)	[Fe] (equiv.)	Yield 3 (%)
**1**	1.75	1.75	67
**2**		2	67
**3**		2.5	66
**4**		3	71
**5**	2	1.75	68
**6**		2	71
**7**		2.5	72
**8**		3	77
**9**	2.5	1.75	69
**10**		2	62
**11**		2.5	80
**12**		3	90
**13**		4	90
**14**	3	1.75	70
**15**		2	77
**16**		2.5	73
**17**		3	75
**18**		4	76

During the optimization, it was noted that the granulometry of $Fe(SO_4)•7H_2O$ had an impact on the outcome of the reaction: finely ground $Fe(SO_4)•7H_2O$ resulted in violent gas evolution and consistently lower yields than commercial samples. On the other hand, selecting only larger particle-sized $Fe(SO_4)•7H_2O$ give a slower reaction but slightly higher yields. However, for convenience, commercial samples containing particles of different sizes were used.

3.3 Reaction Optimization for the Indoles Synthesis From Alkyl Iodides

General Procedure for the Streamlined Indolization Reaction Optimization from Alkyl Iodides – GP6

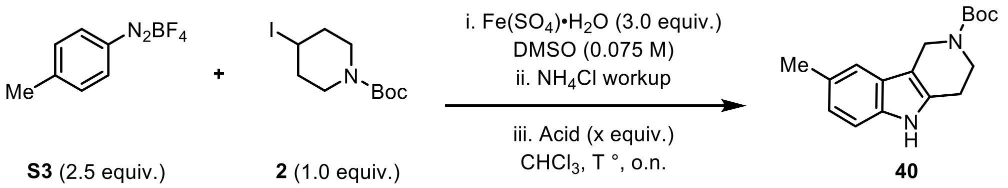

Step i: A dry tube equipped with a stirring bar was charged with *tert*-butyl 4-iodopiperidine-1-carboxylate **2** (0.10 mmol, 1.0 equiv.), $Fe(SO_4)\bullet 7H_2O$ (83 mg, 0.3 mmol, 3.0 equiv.) and 4-methylbenzenediazonium tetrafluoroborate **S3** (55 mg, 0.25 mmol, 2.5 equiv.). The tube was capped with a Supelco aluminium crimp cap with septum (PTFE/butyl), evacuated and refilled with N_2 (x 3). Degassed solvent (1.5 mL, 0.075 M) was added at once. The reaction was stirred at room temperature in the dark for 16 hours. NH_4Cl sat. (3 mL) and EtOAc (3 mL) were added, the mixture was shaken vigorously and the layers were separated. The aqueous layer was extracted with EtOAc, the combined organic layers were dried ($MgSO_4$), filtered and evaporated.

Step ii: The crude material was redissolved in $CHCl_3$ (1 mL, 0.1 M) followed by addition of the acid. The reaction was stirred at the given temperature for 16 hours (if heating was required, the reaction vessels were placed in an oil bath). NH_4Cl sat. (3 mL) and $CHCl_3$ (3 mL) were added and the mixture was shaken vigorously. 1,3-Dinitrobenzene (17 mg, 0.1 mmol, 1.0 equiv.) was added and the layers were separated. The aqueous layer was extracted with $CHCl_3$, the combined organic layers were dried ($MgSO_4$), filtered and evaporated. $CDCl_3$ (0.5 mL) was then added and the mixture was analysed by ^{1}H NMR spectroscopy.

As the results in Table S5 show, reaction with 2 equivalents of trifluoroacetic acid (TFA) at room temperature resulted in a ^{1}H NMR yield of 67% for indole **40** over two steps and marked the endpoint of the optimization (Table S5, entry 11).

Note: 4-methylbenzenediazonium tetrafluoroborate **S3** was used instead of the parent *p*-OMe (**1**) owing to its smaller electronic bias toward cyclization.

Table S5 – Optimization of the Streamlined Cyclization

Entry	Acid	Temperature	Acid (equiv.)	Yield of 39 (over 2 steps)
**1**	MsOH	r.t.	1	58
**2**			1.5	22
**3**			2	15
**4**			3	0
**5**		50°C	1	37
**6**			1.5	9
**7**			2	0
**8**			3	0
**9**	TFA	r.t.	1	14
**10**			1.5	25
**11**			2	67
**12**			3	36
**13**		50°C	1	37
**14**			1.5	34
**15**			2	55
**16**			3	37

3.4 Scope of the Indole Synthesis From Alkyl Iodides

General Procedure for Indole Synthesis – GP7

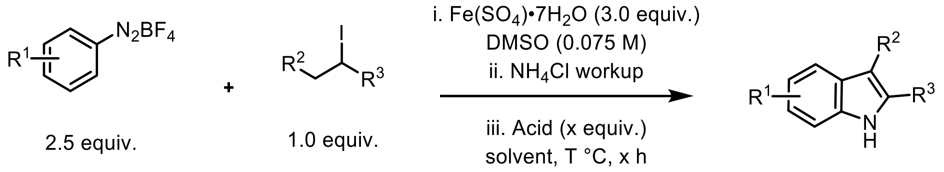

<u>*step i*</u>. A dry 10 mL microwave vial equipped with a stirring bar was charged with $Fe(SO_4)•7H_2O$ (166 mg, 0.6 mmol, 3.0 equiv.) and the diazonium tetrafluoroborate salt (0.5 mmol, 2.5 equiv.). The tube was capped with a Supelco aluminium crimp seal with septum (PTFE/butyl), evacuated and refilled with N_2 (x3). A solution of the alkyl iodide (0.2 mmol, 1.0 equiv.) in DMSO (2.7 mL, 0.075 M) was then added under stirring to the microwave vial containing the solids under N_2 (<u>*caution*</u>: N_2 evolution). The reaction was then stirred at room temperature in the dark for 16 hours.

Aqueous NH_4Cl sat. (3 mL) and EtOAc (3 mL) were added, the mixture was shaken vigorously and the layers were separated. The aqueous layer was extracted with EtOAc (5 mL x2), the combined organic layers were thoroughly washed with brine, dried ($MgSO_4$), filtered and evaporated.

GP7a: <u>*step ii*</u>. The crude material from step *i* was redissolved in dry and degassed $CHCl_3$ (0.1 M) and TFA (2.0 equiv.) were added and the reaction was stirred at the given temperature for 16 h. If heating was required, the reaction vessels were placed in an oil bath. NH_4Cl sat. (3 mL) and $CHCl_3$ (3 mL) were added and the mixture was shaken vigorously. The layers were separated and the aqueous layer was extracted with CH_2Cl_2 (5 mL x3). The combined organic layers were dried (MgSO4), filtered and evaporated. Purification by column chromatography on silica gel gave the products.

GP7b: <u>*step ii*</u>. L-Tartaric acid (6.7 equiv.) and dry and degassed *s*-BuOH (1M) were added to the crude material from step i and the mixture was heated at 90 °C in an oil bath until completion (1–6 hours), indicated by TLC.[15] Aqueous NH_4Cl sat. (3 mL) and EtOAc (3 mL) were added and the mixture was shaken vigorously. The layers were separated and the aqueous layer was extracted with CH_2Cl_2 (5 mL x3). The combined organic layers were dried ($MgSO_4$), filtered and evaporated. Purification by column chromatography on silica gel eluting with a gradient of petrol–EtOAc (up to 30% EtOAc) gave the products.

tert-Butyl 8-Methoxy-1,3,4,5-tetrahydro-2_H_-pyrido[4,3-_b_]indole-2-carboxylate (4)

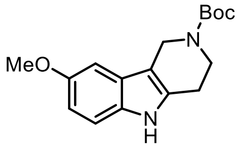

Following **GP7a**, 4-methoxybenzenediazonium tetrafluoroborate **1** (222 mg, 1.0 mmol) and t*ert*-butyl 4-iodopiperidine-1-carboxylate **2** (124 mg, 0.4 mmol) gave **4** as a solid (91 mg, 75%). ^{1}H NMR (400 MHz, CD_3CN) δ 8.88 (1H, br s), 7.21 (1H, d, *J* = 8.7 Hz), 6.93 (1H, s), 6.72 (2H, dd, *J* = 8.7, 2.4 Hz), 4.54 (2H, s), 3.79 (3H, s), 3.74 (2H, t, *J* = 5.8 Hz), 2.77 (2H, t, *J* = 5.7 Hz), 1.47 (9H, s); ^{13}C NMR (101 MHz, CD_3CN) δ 155.9, 154.8, 134.4, 132.0, 126.9, 112.4, 111.5, 107.6, 100.6, 80.1, 56.1, 42.7, 41.5, 28.6, 24.1; HRMS (ESI^{+}): m/z calcd. for $[M]^{+}$ $[C_{17}H_{22}N_2O_3]^{+}$ 302.1630, found 302.1622.

2-(2-(5-Methoxy-1_H_-indol-3-yl)ethyl)isoindoline-1,3-dione (23)

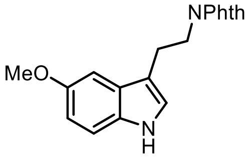

Following **GP7a** but the cyclization step was performed at 50 °C, 4-methoxybenzenediazonium tetrafluoroborate **1** (111 mg, 0.5 mmol) and 2-(4-iodobutyl)isoindoline-1,3-dione **7** (66 mg, 0.2 mmol) gave 2-(2-(5-methoxy-1*H*-indol-3-yl)ethyl)isoindoline-1,3-dione **23** as a solid (20 mg, 31%). ^{1}H NMR (400 MHz, $CDCl_3$) δ 7.95 (1H, br s), 7.87–7.85 (2H, m), 7.74–7.72 (2H, m), 7.26 (1H, d, *J* = 8.8 Hz), 7.20 (1H, d, *J* = 2.5 Hz), 7.11 (1H, d, *J* = 2.5 Hz), 6.86 (1H, dd, *J* = 8.8, 2.5 Hz), 4.61 (2H, br s), 4.02 (2H, t, *J* = 7.8 Hz), 3.89 (3H, s), 3.15 (2H, t, *J* = 7.8 Hz). Data in accordance with the literature.[16]

5-**Methoxy-2-methyl-3-phenyl-1_H_-indole (24)**

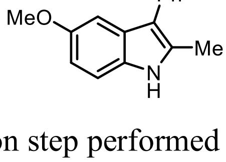

Following **GP3** but with the cyclization step performed using *p*-TsOH·H_2O (76 mg, 0.4 mmol, 1.0 equiv.) in MeCN (0.5 M) for 1 h, 4-methoxybenzenediazonium tetrafluoroborate **1** (222 mg, 1.0 mmol) and (2-iodopropyl)benzene **8** (98 mg, 0.4 mmol) gave 5-methoxy-2-methyl-3-phenyl-1*H*-indole **24** as a solid (42 mg, 44%). ^{1}H NMR (400 MHz, $CDCl_3$) δ 7.87 (1H, br s), 7.58–7.44 (4H, m), 7.35–7.30 (1H, m), 7.22 (2H, d, *J* = 8.7 Hz), 7.16 (1H, d, *J* = 2.5 Hz), 6.85 (1H, dd, *J* = 8.7, 2.5 Hz), 3.84 (3H, s), 2.48 (3H, s); ^{13}C NMR (126 MHz, $CDCl_3$) δ 154.6,

135.7, 132.5, 130.4, 129.4, 128.7, 128.4, 125.9, 114.5, 111.4, 111.1, 101.1, 56.1, 12.7. Data in accordance with the literature.[17]

5-Methoxy-2,3-dimethyl-1*H*-indole (25)

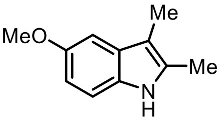

Following **GP7a**, 4-methoxybenzenediazonium tetrafluoroborate **1** (111 mg, 0.5 mmol) and 2-iodobutane **9** (23 µL, 0.2 mmol) gave 5-methoxy-2,3-dimethyl-1*H*-indole **25** as a solid (21 mg, 60%). ^{1}H NMR (400 MHz, $CDCl_3$) δ 7.49 (1H, br s), 7.01 (1H, d, *J* = 8.7 Hz), 6.84 (1H, d, *J* = 2.4 Hz), 6.67 (1H, dd, *J* = 8.6, 2.5 Hz), 3.77 (3H, s), 2.22 (3H, s), 2.10 (3H, s). Data in accordance with the literature.[18]

6-Methoxy-2,3,4,9-tetrahydro-1*H*-carbazole (26)

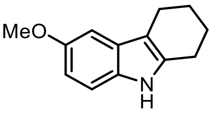

Following **GP7a**, 4-methoxybenzenediazonium tetrafluoroborate **1** (111 mg, 0.5 mmol) and iodocyclohexane **10** (26 µL, 0.2 mmol) gave 6-methoxy-2,3,4,9-tetrahydro-1*H*-carbazole **26** as an oil (29 mg, 73%). ^{1}H NMR (400 MHz, $CDCl_3$) δ 7.56 (1H, br s), 7.16 (1H, d, *J* = 8.2 Hz), 6.94 (1H, d, *J* = 2.1 Hz), 6.77 (1H, dd, *J* = 2.1, 8.2 Hz), 3.86 (3H, s), 2.73–2.66 (4H, m), 1.96–1.83 (4H, m). Data in accordance with the literature.[19]

3,3-Difluoro-6-methoxy-2,3,4,9-tetrahydro-1*H*-carbazole (27)

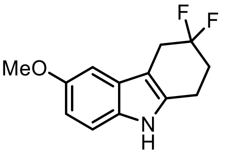

Following **GP7a**, 4-methoxybenzenediazonium tetrafluoroborate **1** (222 mg, 1.0 mmol) and 1,1-difluoro-4-iodocyclohexane **11** (98 mg, 0.4 mmol) gave 3,3-difluoro-6-methoxy-2,3,4,9-tetrahydro-1*H*-carbazole **27** as a solid (66 mg, 70%). ^{1}H NMR (400 MHz, $CDCl_3$) δ 7.70 (1H, br s), 7.17 (1H, d, *J* = 8.2 Hz), 6.90 (1H, d, *J* = 2.8 Hz), 6.83 (1H, dd, *J* = 8.2, 2.8 Hz), 3.87 (3H, s), 3.23 (2H, t, *J* = 14 Hz), 2.96–2.92 (2H, m), 2.39–2.29 (2H, m); ^{13}C NMR (101 MHz, $CDCl_3$) δ 154.3, 132.2 (m), 131.8, 127.6, 124.0 (t, $J_{C–F}$ = 242.0 Hz), 111.6, 111.5, 105.9 (t, $J_{C–F}$ = 6.2 Hz), 100.1, 56.0, 31.7 (t, $J_{C–F}$ = 27.2 Hz), 31.1 (t, $J_{C–F}$ = 25.7 Hz), 20.8 (t, $J_{C–F}$ = 4.0

Hz); ^{19}F NMR (376 MHz, $CDCl_3$) δ (–97.5)–(–97.6) (2F, m); HRMS (APCI$^+$): m/z calcd. for $[M]^+$ $[C_{13}H_{13}F_2NO]^+$ 237.0965, found 237.0967.

6-Methoxy-3-(4-nitrophenyl)-2,3,4,9-tetrahydro-1*H*-carbazole (28)

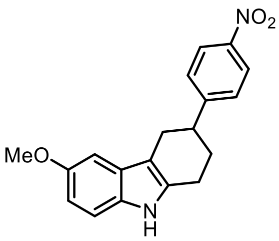

Following **GP7b** (1 h for the cyclization step), 4-methoxybenzenediazonium tetrafluoroborate **1** (111 mg, 0.5 mmol) and 1-(4-iodocyclohexyl)-4-nitrobenzene **12** (66 mg, 0.2 mmol) gave 6-methoxy-3-(4-nitrophenyl)-2,3,4,9-tetrahydro-1*H*-carbazole **28** as an oil (43 mg, 67%). ^{1}H NMR (400 MHz, $CDCl_3$) δ 8.19 (2H, d, J = 8.8 Hz), 7.73 (1H, s), 7.46 (2H d, J = 8.8 Hz), 7.21 (1H, d, J = 8.8 Hz), 6.93 (1H, d, J = 2.4 Hz), 6.82 (1H, dd, J = 8.8, 2.4 Hz), 3.65 (3H, s), 3.25–3.18 (1H, m), 3.08 (1H, dd, J = 14.8, 5.6 Hz), 2.97–2.88 (2H, m), 2.85–2.77 (2H, m), 2.26–2.10 (2H, m); ^{13}C NMR (101 MHz, $CDCl_3$) δ 154.4, 154.1, 146.6, 134.4, 131.2, 128.0, 127.8, 123.9, 111.3, 111.1, 109.3, 100.3, 56.0, 41.2, 30.0, 29.0, 23.2; HRMS (ESI$^+$): m/z calcd. for $[M+Na]^+$ $[C_{19}H_{18}N_2O_3Na]^+$ 345.1210, found 345.1207. requires.

Ethyl 6-Methoxy-2,3,4,9-tetrahydro-1*H*-carbazole-3-carboxylate (29)

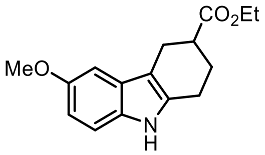

Following **GP7a**, 4-methoxybenzenediazonium tetrafluoroborate **1** (111 mg, 0.5 mmol) and ethyl 4-iodocyclohexane-1-carboxylate **13** (56 mg, 0.2 mmol) gave ethyl 6-methoxy-2,3,4,9-tetrahydro-1*H*-carbazole-3-carboxylate **29** as a solid (34 mg, 62%). ^{1}H NMR (400 MHz, $CDCl_3$) δ 7.66 (1H, br s), 7.16 (1H, d, J = 8.8 Hz), 6.94 (1H, d, J = 2.4 Hz), 6.80 (2H, dd, J = 8.8, 2.4 Hz), 4.21 (2H, q, J = 7.2 Hz), 3.86 (3H, s), 3.04 (1H, dd, J = 14.8, 2.6 Hz), 2.91–2.79 (4H, m), 2.33–2.29 (1H, m), 2.07–1.97 (1H, m), 1.31 (3H, t, J = 7.2 Hz); ^{13}C NMR (101 MHz, $CDCl_3$) δ 175.5, 153.9, 133.9, 130.9, 127.8, 111.1, 110.9, 108.4, 100.1, 60.5, 55.9, 40.3, 25.7, 23.9, 22.4, 14.2; HRMS (ESI$^+$): m/z calcd. for $[M+Na]^+$ $[C_{16}H_{19}NO_3Na]^+$ 296.1257, found 296.1254.

tert-Butyl (6-Methoxy-2,3,4,9-tetrahydro-1*H*-carbazol-3-yl)(methyl)carbamate (30)

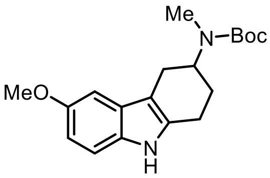

Following **GP7a**, 4-methoxybenzenediazonium tetrafluoroborate **1** (111 mg, 0.5 mmol) and *tert*-butyl (4-iodocyclohexyl)(methyl)carbamate **14** (68 mg, 0.2 mmol) gave *tert*-butyl (6-methoxy-2,3,4,9-tetrahydro-1*H*-carbazol-3-yl)(methyl)carbamate **30** as a solid (42 mg, 65%). ^{1}H NMR (400 MHz, $CDCl_3$) δ 7.85 (1H, br s), 7.15 (1H, d, *J* = 8.4 Hz), 6.89 (1H, br s), 6.77 (1H, d, *J* = 8.4 Hz), 4.52–4.36 (1H, m), 3.85 (3H, s), 2.93–2.75 (7H, m), 2.05–1.98 (2H, m), 1.50 (9H, s); ^{13}C NMR (101 MHz, $CDCl_3$, rotamers) δ 155.7, 153.8, 133.6, 131.3, 128.0, 111.1, 110.8, 108.6, 100.0, 79.5, 55.9, 52.7, 51.6, 39.9, 28.5, 27.4, 24.2, 23.0; HRMS (ESI$^+$): m/z calcd. for $[M+Na]^+$ $[C_{18}H_{24}N_2O_4Na]^+$ 353.1836, found 353.1828.

6-Methoxy-1,2,4,9-tetrahydrospiro[carbazole-3,2'-[1,3]dioxolane] (31)

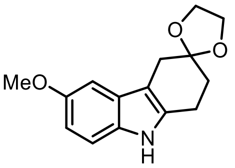

Following **GP7a**, 4-methoxybenzenediazonium tetrafluoroborate **1** (111 mg, 0.5 mmol) and 8-iodo-1,4-dioxaspiro[4.5]decane **15** (53 mg, 0.2 mmol, 1.0 equiv.) gave 6-methoxy-1,2,4,9-tetrahydrospiro[carbazole-3,2'-[1,3]dioxolane] **31** as an oil (34 mg, 66%). ^{1}H NMR (400 MHz, $CDCl_3$) δ 7.65 (1H, br s), 7.15 (1H, d, *J* = 8.7 Hz), 6.87 (1H, d, *J* = 2.5 Hz), 6.76 (1H, dd, *J* = 8.7, 2.5 Hz), 4.18–3.96 (4H, m), 3.84 (3H, s), 2.97–2.88 (4H, m), 2.07 (2H, t, *J* = 6.5 Hz); ^{13}C NMR (101 MHz, $CDCl_3$) δ 154.1, 133.4, 131.8, 128.2, 111.2, 111.0, 109.3, 108.3, 100.3, 64.8, 56.1, 32.2, 32.0, 21.8; HRMS (ESI$^+$): m/z calcd. for $[M]^+$ $[C_{15}H_{17}NO_3]^+$ 259.1208, found M^+ 259.1196.

8-Methoxy-1,3,4,5-tetrahydrothiopyrano[4,3-*b*]indole (32)

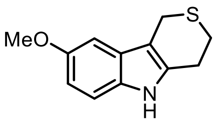

Following **GP7a**, 4-methoxybenzenediazonium tetrafluoroborate **1** (222 mg, 1.0 mmol) and 4-iodotetrahydro-2*H*-thiopyran **16** (91 mg, 0.4 mmol, 1.0 equiv.) gave 8-methoxy-1,2,4,5-

tetrahydrothiopyrano[4,3-*b*]indole **32** as an oil (55 mg, 63%). ^{1}H NMR (500 MHz, $CDCl_3$) δ 7.71 (1H, br s), 7.17 (1H, d, *J* = 8.7 Hz), 6.90 (1H, d, *J* = 2.5 Hz), 6.80 (1H, dd, *J* = 8.7, 2.5 Hz), 3.86 (3H, s), 3.83 (2H, s), 2.99 (4H, app s); ^{13}C NMR (126 MHz, $CDCl_3$) δ 154.1, 134.1, 129.6, 127.4, 111.3, 111.2, 106.6, 100.0, 56.0, 25.7, 25.3, 22.8; HRMS (APCI$^+$): m/z calcd. for $[M]^+$ $[C_{12}H_{13}NOS]^+$ 219.0718, found 219.0718.

***tert*-Butyl 8-Methoxy-2,3,4,5-tetrahydro-1*H*-pyrido[3,2-*b*]indole-1-carboxylate (33)**

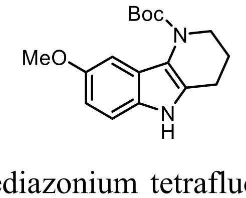

Following **GP7a**, 4-methoxybenzenediazonium tetrafluoroborate **1** (222 mg, 1.0 mmol) and *tert*-butyl 3-iodopiperidine-1-carboxylate **17** (124 mg, 0.4 mmol) gave *tert*-butyl 8-methoxy-2,3,4,5-tetrahydro-1*H*-pyrido[3,2-*b*]indole-1-carboxylate **33** as an oil (23 mg, 19%). ^{1}H NMR (400 MHz, $CDCl_3$) δ 7.59 (1H, br s), 7.15–7.06 (2H, m), 6.78 (1H, dd, *J* = 8.8, 2.5 Hz), 3.86 (3H, s), 3.79–3.72 (2H, m), 2.81 (2H, t, *J* = 6.8 Hz), 2.11–1.99 (2H, m), 1.54 (9H, s); ^{13}C NMR (101 MHz, $CDCl_3$) δ 154.3, 153.5, 129.8, 126.4, 123.1, 116.9, 111.6, 111.2, 103.3, 80.6, 56.0, 45.2, 28.7, 23.2, 21.8; HRMS (ESI$^+$): m/z calcd. for $[M]^+$ $[C_{17}H_{22}N_2O_3]^+$ 302.1630, found 302.1614.

***tert*-Butyl 9-Methoxy-1,4,5,6-tetrahydroazepino[4,5-*b*]indole-3(2*H*)-carboxylate (34)**

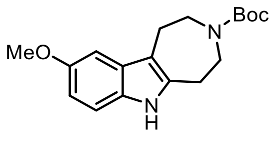

Following **GP7a**, 4-methoxybenzenediazonium tetrafluoroborate **11** (222 mg, 1.0 mmol) and *tert*-butyl 4-iodoazepane-1-carboxylate **18** (130 mg, 0.4 mmol) gave *tert*-butyl 9-methoxy-1,4,5,6-tetrahydroazepino[4,5-*b*]indole-3(2*H*)-carboxylate **34** as an oil (67 mg, 53%). ^{1}H NMR (400 MHz, $CDCl_3$, rotamers) δ 7.75 & 7.71 (1H, br s), 7.16 (1H, d, *J* = 8.7 Hz), 6.91 (1H, d, *J* = 2.4 Hz), 6.78 (1H, dd, *J* = 8.7, 2.4 Hz), 3.85 (3H, s), 3.76–3.58 (4H, m), 3.05–2.82 (4H, m), 1.49 (9H, s); ^{13}C NMR (101 MHz, $CDCl_3$, rotamers) δ 155.2, 154.9, 154.2, 136.0, 135.5, 129.8, 129.7, 111.9, 111.5, 111.4, 111.2, 111.15, 111.1, 111.0, 100.2, 79.8, 56.1, 49.3, 48.7, 47.4, 46.8, 30.2, 29.7, 28.7, 25.5, 24.9; HRMS (APCI$^+$): m/z calcd. for $[M+H]^+$ 317.1860, found 317.1858.

5'-Iodo-6-methoxy-1'-methyl-2,3,4,9-tetrahydrospiro[carbazole-1,3'-indolin]-2'-one (35)

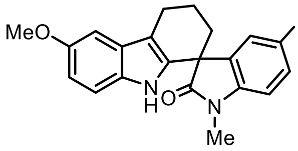

Following **GP7a,** 4-methoxybenzenediazonium tetrafluoroborate **1** (111 mg, 0.5 mmol) and 2,5'-diiodo-1'-methylspiro[cyclohexane-1,3'-indolin]-2'-one **20** (93 mg, 0.2 mmol) gave 5'-iodo-6-methoxy-1'-methyl-2,3,4,9-tetrahydrospiro[carbazole-1,3'-indolin]-2'-one **35** as a solid (40 mg, 44%). ^{1}H NMR (400 MHz, THF-*d*$_8$) δ 9.25 (br s, 1H), 7.63 (1H, dd, *J* = 8.0, 1.6 Hz), 7.39 (1H, d, *J* = 1.6 Hz), 6.97–6.91 (2H, m), 6.82 (1H, d, *J* = 8.0 Hz), 6.65 (1H, dd, *J* = 8.8, 2.4 Hz), 3.77 (3H, s), 3.18 (3H, s), 2.84 (2H, t, *J* = 6.4 Hz), 2.46–2.40 (1H, m), 2.19–2.14 (1H, m) 2.01–1.94 (2H, m); ^{13}C NMR (101 MHz, THF-*d*$_8$) δ 177.6, 154.9, 144.6, 138.0, 137.6, 133.3, 133.0, 132.1, 128.4, 113.6, 112.4, 112.1, 111.0, 100.9, 85.3, 55.9, 49.8, 36.2, 26.4, 21.6, 20.3; HRMS (ESI$^+$): m/z calcd. for [M]$^+$ [$C_{21}H_{19}IN_2O_2$]$^+$ 458.0491,found 458.0488.

(3a*S*,3b*R*,5a*S*,12a*S*,12b*S*,14a*S*)-10-Methoxy-12a,14a-dimethyl-3,3a,3b,4,5,5a,6,7,12,12a,12b,13,14,14a-tetradecahydrocyclopenta[5,6]naphtho[2,1-*b*]carbazol-1(2*H*)-one (36)

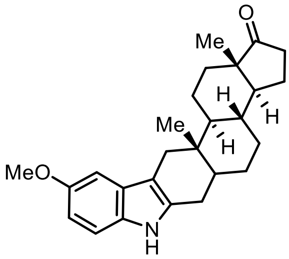

Following **GP7a** but using DMSO:DCE (1:1, 6 mL, 0.034 M) as the solvent, 4-methoxybenzenediazonium tetrafluoroborate **1** (178 mg, 0.8 mmol, 4.0 equiv.) and $FeSO_4·7H_2O$ (266 mg, 0.96 mmol, 4.8 equiv.), androsteryl iodide **21** (80 mg, 0.2 mmol) gave **36** as a solid (41 mg, 52%). ^{1}H NMR (400 MHz, $CDCl_3$) δ 7.57 (1H, s), 7.15 (1H, d, *J* = 7.2 Hz), 6.90 (1H, d, *J* = 2.4 Hz), 7.76 (1H, dd, *J* = 7.2, 2.4 Hz), 3.85 (3H, s), 2.75 (1H, d, *J* = 15.2 Hz), 2.62–2.56 (1H, m), 2.51–2.37 (2H, m), 2.30 (1H, d, *J* = 15.2 Hz), 2.15–2.06 (1H, m), 2.01–2.85 (4H, m), 1.69–1.23 (7H, m), 1.11–0.88 (3H, m), 0.92 (3H, s), 0.84 (3H, s); ^{13}C NMR (101 MHz, $CDCl_3$) δ 221.6, 154.0, 133.6, 131.1, 128.7, 111.2, 110.8, 109.2, 100.1, 56.0, 54.2, 51.5, 47.9, 42.6, 36.6, 36.0, 35.7, 35.4, 31.8, 30.8, 29.1, 28.1, 22.0, 20.7, 13.9, 11.9; HRMS (ASAP): m/z calcd. for [M]$^-$ [$C_{26}H_{33}NO_2$]$^-$ 390.2439, found 390.2444.

***tert*-Butyl (2-Amino-3,5-dibromobenzyl)(6-methoxy-2,3,4,9-tetrahydro-1*H*-carbazol-3-yl)carbamate (37)**

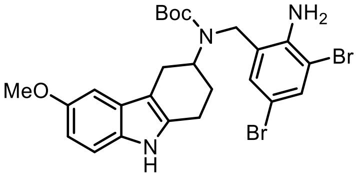

Following **GP7a**, 4-methoxybenzenediazonium tetrafluoroborate **1** (111 mg, 0.5 mmol, 2.5 equiv.) and *tert*-butyl (2-amino-3,5-dibromobenzyl)(4-iodocyclohexyl)carbamate **22** (118 mg, 0.2 mmol, 1.0 equiv.) gave *tert*-butyl (2-amino-3,5-dibromobenzyl)(6-methoxy-2,3,4,9-tetrahydro-1*H*-carbazol-3-yl)carbamate **37** as a solid (62 mg, 54%). ^{1}H NMR (400 MHz, THF-d_8) δ 9.54 (1H, s), 7.46 (1H, d, J = 2.4 Hz), 7.24 (1H, d, J = 2.4 Hz), 7.03 (1H, d, J = 8.4 Hz), 6.80 (1H, d, J = 2.4 Hz) 6.62 (1H, dd, J = 8.8, 2.4 Hz), 5.38 (2H, br s), 4.49–4.37 (2H, m), 4.22–3.82 (1H, m), 3.74 (3H, s), 2.98–2.92 (1H, m), 2.82–2.72 (3H, m), 2.20 (1H, br s), 1.81–1.77 (1H, m), 1.46 (9H, s); ^{13}C NMR (101 MHz, THF-d_8, rotamers) δ 157.0, 154.8, 143.9, 134.4 & 134.3, 134.1, 133.0 & 132.9, 132.5, 129.1 & 129.0, 126.3, 111.6 & 111.5, 111.1, 109.9 & 109.8, 108.6 & 108.6, 107.6, 100.4, 80.8, 56.3, 55.8, 47.9, 30.8 & 30.7, 29.1, 28.7, 24.2; HRMS (ESI$^+$): m/z calcd. for [M+Na]$^+$ [$C_{25}H_{29}Br_2N_3O_3Na$]$^+$ 602.0448, found 602.0453.

***tert*-Butyl 1,3,4,5-Tetrahydro-2*H*-pyrido[4,3-*b*]indole-2-carboxylate (38)**

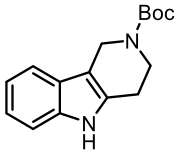

Following **GP7b** (90 minutes for the cyclization step), benzenediazonium tetrafluoroborate **S2** (96 mg, 0.5 mmol) and tert-butyl 4-iodopiperidine-1-carboxylate **2** (62 mg, 0.2 mmol) gave *tert*-butyl 1,3,4,5-tetrahydro-2*H*-pyrido[4,3-*b*]indole-2-carboxylate **38** as an oil (29 mg, 54%). ^{1}H NMR (400 MHz, $CDCl_3$) δ 8.02 (1H, br s), 7.45 (1H, d, J = 6.4 Hz), 7.31 (1H, d, J = 6.4 Hz), 7.17–7.09 (2H, m), 4.65 (2H, s), 3.90–3.75 (2H, m), 2.82 (2H, t, J = 4.8 Hz), 1.54 (9H, s); ^{13}C NMR (126 MHz, $CDCl_3$, rotamers) δ 155.4 & 154.6, 136.0, 132.4 & 132.0, 125.7, 121.7, 119.7, 117.6, 110.9, 107.6, 80.6 & 80.0, 41.6 & 41.3, 40.7, 28.6 & 28.5, 23.6; HRMS (APCI$^+$): m/z calcd. for [M+H]$^+$ [$C_{16}H_{21}N_2O_2$]$^+$ 273.1598, found 273.1590.

tert-Butyl 5-Methoxy-2,3,4,5-tetrahydro-1H-pyrido[3,2-b]indole-1-carboxylate (39)

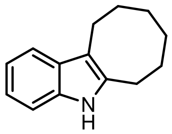

Following **GP7a**, benzenediazonium tetrafluoroborate **S2** (96 mg, 0.5 mmol) and iodocyclooctane **19** (24 mg, 0.2 mmol) gave *tert*-butyl 5-methoxy-2,3,4,5-tetrahydro-1*H*-pyrido[3,2-*b*]indole-1-carboxylate **39** as a solid (20 mg, 50%). ^{1}H NMR (400 MHz, $CDCl_3$) δ 7.54 (1H, br s), 7.42–7.39 (1H, m), 7.18–7.13 (1H, m), 7.04–6.97 (2H, m), 2.79–2.71 (4H, m), 1.69–1.61 (4H, m), 1.41–1.29 (4H, m); ^{13}C NMR (101 MHz, $CDCl_3$) δ 135.7, 135.1, 128.7, 120.7, 119.0, 117.8, 111.7, 110.4, 42.1, 29.7, 29.6, 26.1, 26.0, 22.3; HRMS (APCI$^+$): m/z calcd. for $[M+H]^+$ $[C_{14}H_{18}N]^+$ 200.1434, found 200.1425.

tert-Butyl 8-Methyl-1,3,4,5-tetrahydro-2H-pyrido[4,3-b]indole-2-carboxylate (40)

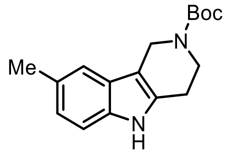

Following **GP7a**, 4-methylbenzenediazonium tetrafluoroborate **S3** (103 mg, 0.5 mmol) and *tert*-butyl 4-iodopiperidine-1-carboxylate **2** (62 mg, 0.2 mmol) gave *tert*-butyl 8-methyl-1,3,4,5-tetrahydro-2*H*-pyrido[4,3-*b*]indole-2-carboxylate **40** as a solid (38 mg, 67%). ^{1}H NMR (400 MHz, $CDCl_3$) δ 8.00 (1H, br s), 7.27 (1H, s), 7.22 (1H, d, J = 8.2 Hz), 7.01 (1H, d, J = 8.2 Hz), 4.65 (2H, s), 3.90–3.85 (2H, m), 2.83 (2H, t, J = 5.4 Hz), 2.48 (3H, s), 1.55 (9H, s). Data in accordance with the literature.[20]

tert-Butyl 8-(tert-Butyl)-1,3,4,5-tetrahydro-2H-pyrido[4,3-b]indole-2-carboxylate (41)

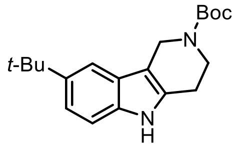

Following **GP7b** (30 minutes for the cyclization step), 4-(*tert*-butyl)benzenediazonium tetrafluoroborate **S4** (124 mg, 0.5 mmol) and *tert*-butyl 4-iodopiperidine-1-carboxylate **2** (62 mg, 0.2 mmol) gave *tert*-butyl 8-(*tert*-butyl)-1,3,4,5-tetrahydro-2*H*-pyrido[4,3-*b*]indole-2-carboxylate **41** as an oil (38 mg, 58%). ^{1}H NMR (500 MHz, $CDCl_3$) δ 7.84 (1H, br s), 7.43 (1H, s), 7.30–7.18 (2H, m), 4.65 (2H, s), 3.87–3.76 (2H, m), 2.86–2.75 (2H, m), 1.52 (9H, s), 1.39 (9H, s); ^{13}C NMR (101 MHz, $CDCl_3$, rotamers) δ 155.5 & 154.6, 142.7, 134.1, 132.5, 125.5,

119.9, 113.5, 110.3, 107.5, 80.6 & 80.0, 41.6 & 41.3, 40.8, 34.7, 32.1, 28.7 & 28.5, 23.7; HRMS (APCI$^+$): m/z calcd. for $[M]^+$ $[C_{20}H_{28}N_2O_2]^+$ 328.2151, found 328.2149.

***tert*-Butyl 8-Chloro-1,3,4,5-tetrahydro-2*H*-pyrido[4,3-*b*]indole-2-carboxylate (42)**

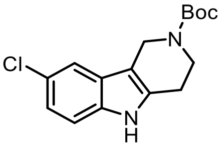

Following **GP7b** (6 h for the cyclization step), 4-chlorobenzenediazonium tetrafluoroborate **S5** (113 mg, 0.5 mmol) and *tert*-butyl 4-iodopiperidine-1-carboxylate **2** (62 mg, 0.2 mmol) gave *tert*-butyl 8-chloro-1,3,4,5-tetrahydro-2*H*-pyrido[4,3-*b*]indole-2-carboxylate **42** as a solid (24 mg, 39%). ^{1}H NMR (400 MHz, $CDCl_3$) δ 8.24 (1H, s), 7.44–7.38 (1H, m), 7.25–7.21 (1H, m), 7.11 (1H, d, J = 8.4 Hz,), 4.62 (2H, s), 3.90–3.79 (2H, m), 2.83 (2H, t, J = 5.6 Hz), 1.54 (9H, s). Data in accordance with the literature.[20]

***tert*-Butyl 8-Bromo-1,3,4,5-tetrahydro-2*H*-pyrido[4,3-*b*]indole-2-carboxylate (43)**

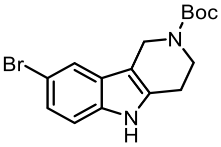

Following **GP7a** (cyclization step performed at 50 °C), 4-bromobenzenediazonium tetrafluoroborate **S6** (135 mg, 0.5 mmol) and *tert*-butyl 4-iodopiperidine-1-carboxylate **2** (62 mg, 0.2 mmol) gave *tert*-butyl 8-bromo-1,3,4,5-tetrahydro-2*H*-pyrido[4,3-*b*]indole-2-carboxylate **43** as a solid (48 mg, 69%). ^{1}H NMR (600 MHz, CD_3CN) δ 9.31 (1H, br s), 7.55–7.52 (1H, m), 7.22 (1H, d, J = 8.6 Hz), 7.15 (1H, dd, J = 8.6, 1.9 Hz), 4.52 (2H, s), 3.73 (2H, t, J = 5.7 Hz), 2.77 (2H, t, J = 5.7 Hz), 1.46 (9H, s). Data in accordance with the literature.[21]

Following **GP7a** (cyclization step performed at 50 °C), 4-bromobenzenediazonium tetrafluoroborate **S6** (675 mg, 2.5 mmol) and *tert*-butyl 4-iodopiperidine-1-carboxylate **2** (310 mg, 1.0 mmol) gave *tert*-butyl 8-bromo-1,3,4,5-tetrahydro-2*H*-pyrido[4,3-*b*]indole-2-carboxylate **43** as a solid (235 mg, 67%).

***tert*-Butyl 7,8-Dimethoxy-1,3,4,5-tetrahydro-2*H*-pyrido[4,3-*b*]indole-2-carboxylate (44)**

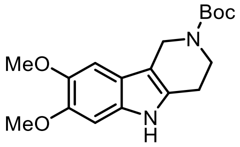

Following **GP7a**, 3,4-dimethoxybenzenediazonium tetrafluoroborate **S7** (126 mg, 0.5 mmol) and *tert*-butyl 4-iodopiperidine-1-carboxylate **2** (62 mg, 0.2 mmol) gave *tert*-butyl 7,8-dimethoxy-1,3,4,5-tetrahydro-2*H*-pyrido[4,3-*b*]indole-2-carboxylate **44** as a solid (45 mg, 69%). ^{1}H NMR (400 MHz, $CDCl_3$) δ 7.97 (1H, br s), 6.88 (1H, s), 6.83 (1H, s), 4.59 (2H, s), 3.93 (3H, s), 3.87 (3H, s), 3.83–3.73 (2H, m), 2.77 (2H, t, J = 5.6 Hz), 1.51 (9H, s); ^{13}C NMR (101 MHz, $CDCl_3$) δ 155.4, 146.6, 144.9, 130.8, 130.2, 118.4, 107.3, 100.0, 94.9, 80.0, 56.5, 56.4, 41.6, 40.8, 28.6, 23.7; HRMS (ESI$^+$): m/z calcd. for $[M+Na]^+$ $[C_{18}H_{24}N_2O_4Na]^+$ 355.1628, found 355.1635.

***tert*-Butyl 5,6,7,9-Tetrahydro-8*H*-[1,3]dioxolo[4,5-*f*]pyrido[4,3-*b*]indole-8-carboxylate (45)**

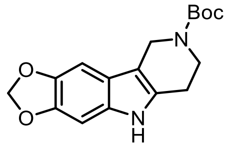

Following **GP7a**, benzo[*d*][1,3]dioxole-5-diazonium tetrafluoroborate **S8** (118 mg, 0.5 mmol) and *tert*-butyl 4-iodopiperidine-1-carboxylate **2** (62 mg, 0.2 mmol) gave *tert*-butyl 5,6,7,9-tetrahydro-8*H*-[1,3]dioxolo[4,5-*f*]pyrido[4,3-*b*]indole-8-carboxylate **45** as a solid (34 mg, 54%). ^{1}H NMR (400 MHz, $CDCl_3$) δ 7.87 (1H, br s), 6.83 (1H, s), 6.79 (1H, s), 5.91 (2H, s), 4.56 (2H, s), 3.85–3.73 (2H, m), 2.77 (2H, t, J = 5.6 Hz), 1.50 (9H, s); ^{13}C NMR (101 MHz, $CDCl_3$) δ 155.3, 144.5, 142.7, 130.9, 130.7, 119.5, 107.8, 100.6, 96.9, 92.3, 80.0, 41.5, 40.9, 28.6, 23.7; HRMS (ESI$^+$): m/z calcd. for $[M+Na]^+$ $[C_{17}H_{20}N_2O_4Na]^+$ 339.1315, found 339.1309.

***tert*-Butyl 6-Methoxy-1,3,4,5-tetrahydro-2*H*-pyrido[4,3-*b*]indole-2-carboxylate (46)**

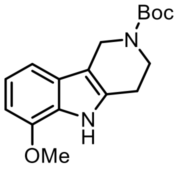

Following **GP7b** (90 minutes for the cyclization step), 2-methoxybenzenediazonium tetrafluoroborate **S9** (111 mg, 0.5 mmol) and *tert*-butyl 4-iodopiperidine-1-carboxylate **2** (62

mg, 0.2 mmol) gave *tert*-butyl 6-methoxy-1,3,4,5-tetrahydro-2*H*-pyrido[4,3-*b*]indole-2-carboxylate **46** as a solid (27 mg, 45%). ^{1}H NMR (400 MHz, $CDCl_3$) δ 8.12 (1H, br s), 7.06–7.01 (2H, m), 6.64 (1H, d, *J* = 7.6 Hz), 4.63 (2H, s), 3.95 (3H, s), 3.87–3.75 (2H, m), 2.89–2.73 (2H, m), 1.51 (9H, s); ^{13}C NMR (101 MHz, $CDCl_3$) δ 155.4, 145.9, 131.8, 126.9, 126.2, 120.1, 110.6, 108.1, 102.1, 80.0, 55.4, 41.7, 40.8, 28.5, 23.6; HRMS (ASAP): m/z calcd. for $[M]^-$ $[C_{17}H_{22}N_2O_3]^-$ 301.1558, found 301.1556.

tert-Butyl 6-Ethyl-1,3,4,5-tetrahydro-2*H*-pyrido[4,3-*b*]indole-2-carboxylate (47)

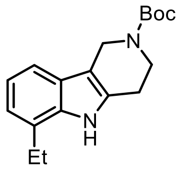

Following **GP7a** (cyclization step performed at 50 °C), 2-ethylbenzenediazonium tetrafluoroborate **S10** (110 mg, 0.5 mmol) and *tert*-butyl 4-iodopiperidine-1-carboxylate **2** (62 mg, 0.2 mmol) gave *tert*-butyl 6-ethyl-1,3,4,5-tetrahydro-2*H*-pyrido[4,3-*b*]indole-2-carboxylate **47** as a solid (31 mg, 51%). ^{1}H NMR (500 MHz, $CDCl_3$) δ 7.93 (1H, br s), 7.31 (1H, d, *J* = 7.6 Hz), 7.09–7.00 (2H, m), 4.64 (2H, s), 3.88–3.76 (2H, m), 2.88–2.81 (4H, m), 1.51 (9H, s), 1.36 (3H, t, *J* = 7.6 Hz); ^{13}C NMR (126 MHz, $CDCl_3$) δ 155.4, 134.7, 132.0, 126.3, 125.4, 120.5, 120.0, 115.4, 112.8, 80.0, 41.3, 28.6, 28.6, 28.5, 24.3, 23.7, 14.1; HRMS (ESI$^+$): m/z calcd. for $[M+Na]^+$ $[C_{18}H_{24}N_2O_2Na]^+$ 323.1730, found 323.1735.

tert-Butyl 5,6,7,9-Tetrahydro-8*H*-pyrido[4,3-*b*]thieno[2,3-*f*]indole-8-carboxylate (48)

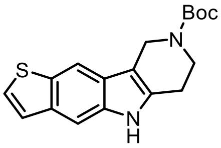

Following GP7a, benzo[*b*]thiophene-5-diazonium tetrafluoroborate S11 (124 mg, 0.5 mmol) and *tert*-butyl 4-iodopiperidine-1-carboxylate 2 (62 mg, 0.2 mmol) gave *tert*-butyl 5,6,7,9-tetrahydro-8*H*-pyrido[4,3-*b*]thieno[2,3-*f*]indole-8-carboxylate 48 as a solid (21 mg, 34%). ^{1}H NMR (400 MHz, $CDCl_3$) δ 8.16 (1H, br s), 7.61 (1H, d, *J* = 8.8 Hz), 7.56–7.54 (1H, m), 7.51 (1H, br s), 7.33 (1H, d, *J* = 8.8 Hz), 4.93 (2H, s), 3.93–3.75 (2H, m), 2.89 (2H, t, *J* = 2.4 Hz), 1.53 (9H, s); ^{13}C NMR (101 MHz, $CDCl_3$, rotamers) δ 155.4, 132.8, 132.7, 131.7, 131.1, 125.8, 121.7, 120.1, 115.8, 109.4, 108.1, 80.1, 42.7 & 42.4, 40.6, 28.6, 23.7; HRMS (ASAP): m/z calcd. for $[M]^-$ $[C_{18}H_{20}N_2O_2S]^-$ 327.1173, found 327.1178.

3.5 Procedure for 1 mmol scale reaction

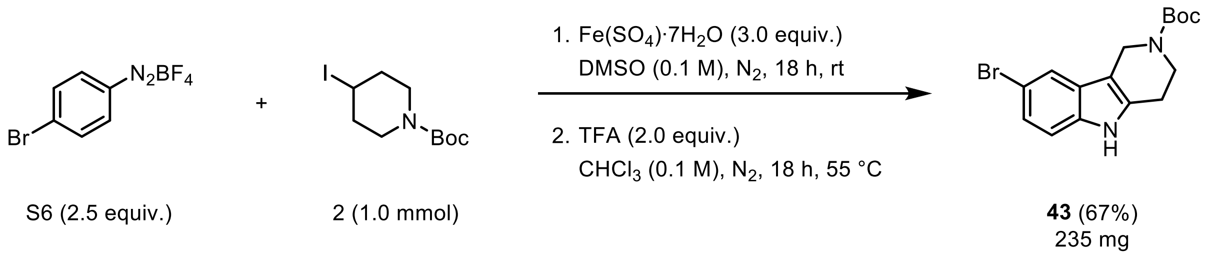

Step i: A 35 mL-microwave vial was charged with a stirring bar, $Fe(SO_4)\cdot 7H_2O$ (830 mg, 3.0 mmol, 3.0 equiv.) and 4-bromophenyldiazonium tetrafluoroborate S6 (675 mg, 2.5 mmol, 2.5 equiv.), capped, placed under N_2 atmosphere and fitted with a balloon (Figure S1). A stock solution of *tert*-butyl 4-iodopiperidine-1-carboxylate 2 (310 mg, 1.0 mmol, 1.0 equiv.) in dry and degassed DMSO (10 mL) was added rapidly to the microwave vial under stirring. At this point, evolution of gas from the heterogeneous mixture is observed along with a colour change from yellow to bright red. The mixture was stirred overnight at room temperature, quenched with NH_4Cl sat. (10 mL) and extracted with EtOAc (50 mL x 3). The combined organic layers were thoroughly washed with brine (200 mL x 3), dried over $MgSO_4$, filtered, and evaporated.

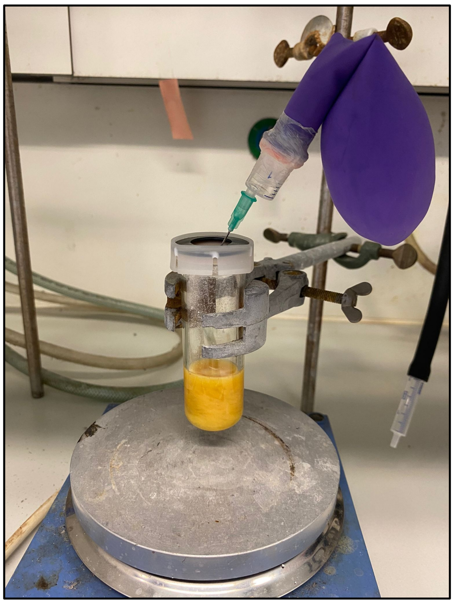

Figure S2 - Crude mixture from step i.

Step ii: The crude mixture from step i was placed under N_2 atmosphere in a round-bottom flask capped with a septum. Anhydrous and degassed $CHCl_3$ (5 mL) was added (a red solution is observed) and the contents of the flask were transferred via syringe to an oven-dried 35 mL microwave vial equipped with a stirring bar under N_2 atmosphere. This was repeated two times

(2.5 mL x 2) before addition of TFA (151 mL, 2.0 mmol, 2.0 equiv.) upon which the solution immediately darkens. The vial was sealed with parafilm, placed in a pre-heated oil bath, and stirred overnight at 55 °C. The crude mixture was evaporated under reduced pressure, redissolved in CH_2Cl_2 (10 mL) and washed with $NaHCO_3$ sat. (10 mL). The aqueous layer was extracted with CH_2Cl_2 (20 mL x 3), dried over $MgSO_4$, filtered, and evaporated. The crude mixture was purified by column chromatography on silica gel eluting petrol–EtOAc (7:3) to give 43 as a solid (235 mg, 67%).

4 Mechanistic Studies

4.1 HRMS Detection of Fe(III)

The aqueous layer after NH_4Cl sat. work-up of the first step (formation of the diazene **3**) was submitted to HRMS. $[FeCl_4]^-$ was identified by HRMS (ESI^-): m/z calcd. for $[M]^-$ $[FeCl_4]^-$ 197.8109, found 197.8106 (Δ 1.51 ppm).

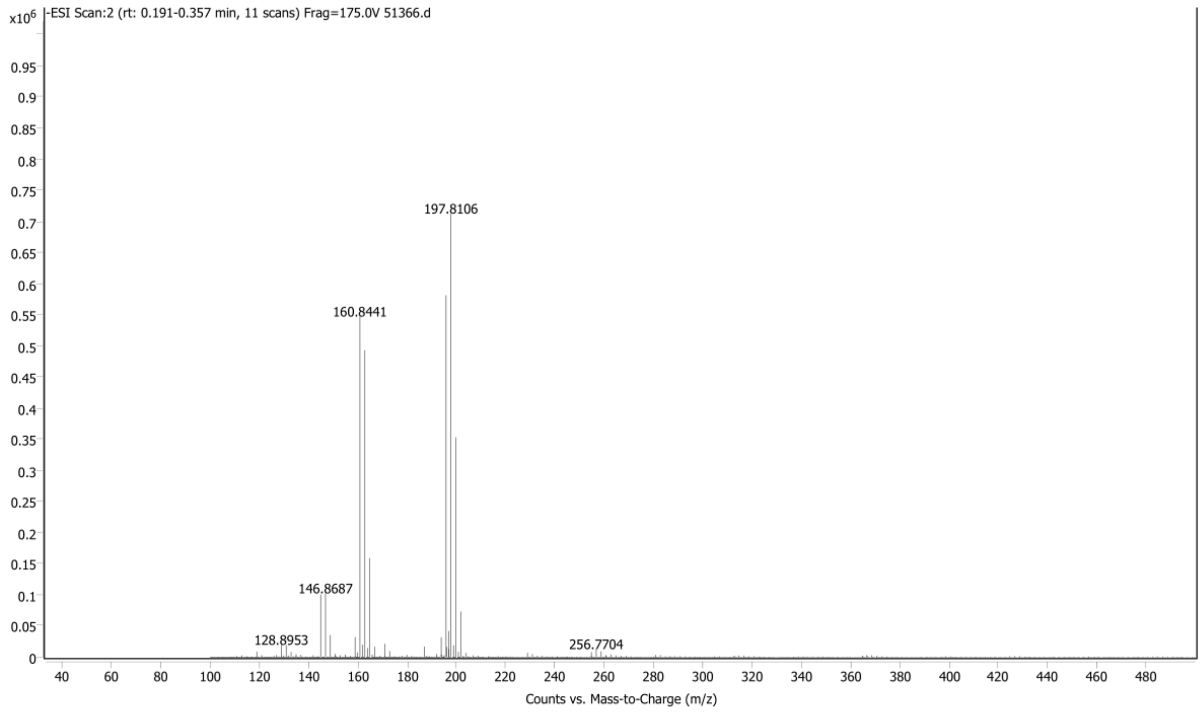

4.2 Prussian Blue Test

In order to support the formation of Fe(III), diagnostic of the SET oxidation of $Fe(SO_4)$•$7H_2O$, we added $K_4[Fe(II)(CN)_6]$•$3H_2O$ to the crude reaction mixture. A characteristic deep Prussian blue color immediately appeared, confirming the existence of Fe(III) salts in solution (see Figure S2). No Prussian blue colour was noticed in the control experiment in the absence of diazonium salt **1**.

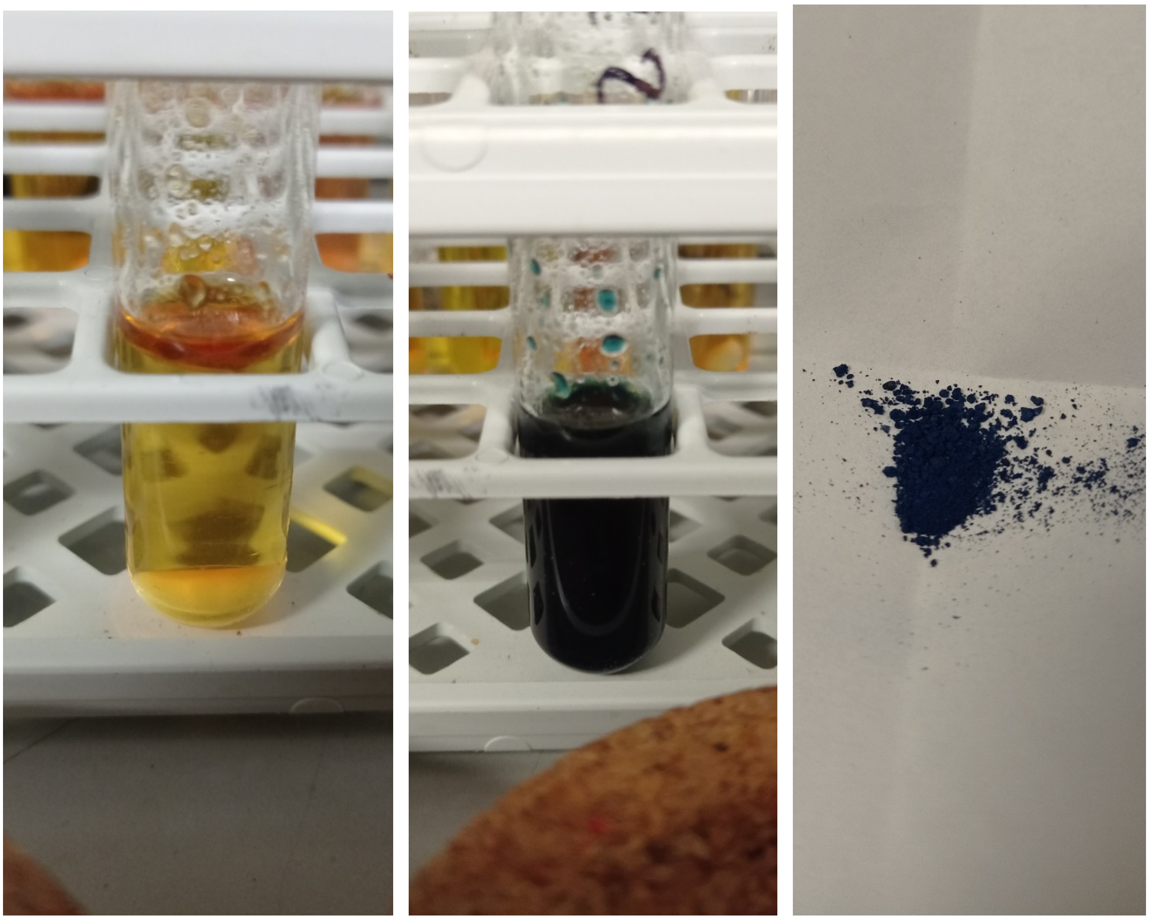

Figure S2 – From left to right: aqueous layer of the diazene **3** formation after NH_4Cl sat. workup (the bright yellow colour is due to $FeCl_4^-$), aqueous layer after addition of $K_4[Fe(II)(CN)_6]\cdot3H_2O$ (formation of Prussian Blue, $Fe^{III}{}_4[Fe^{II}(CN)_6]$), isolated Prussian Blue solid.

5 NMR Spectra

S1 – ¹H NMR (400 MHz, CDCl₃)

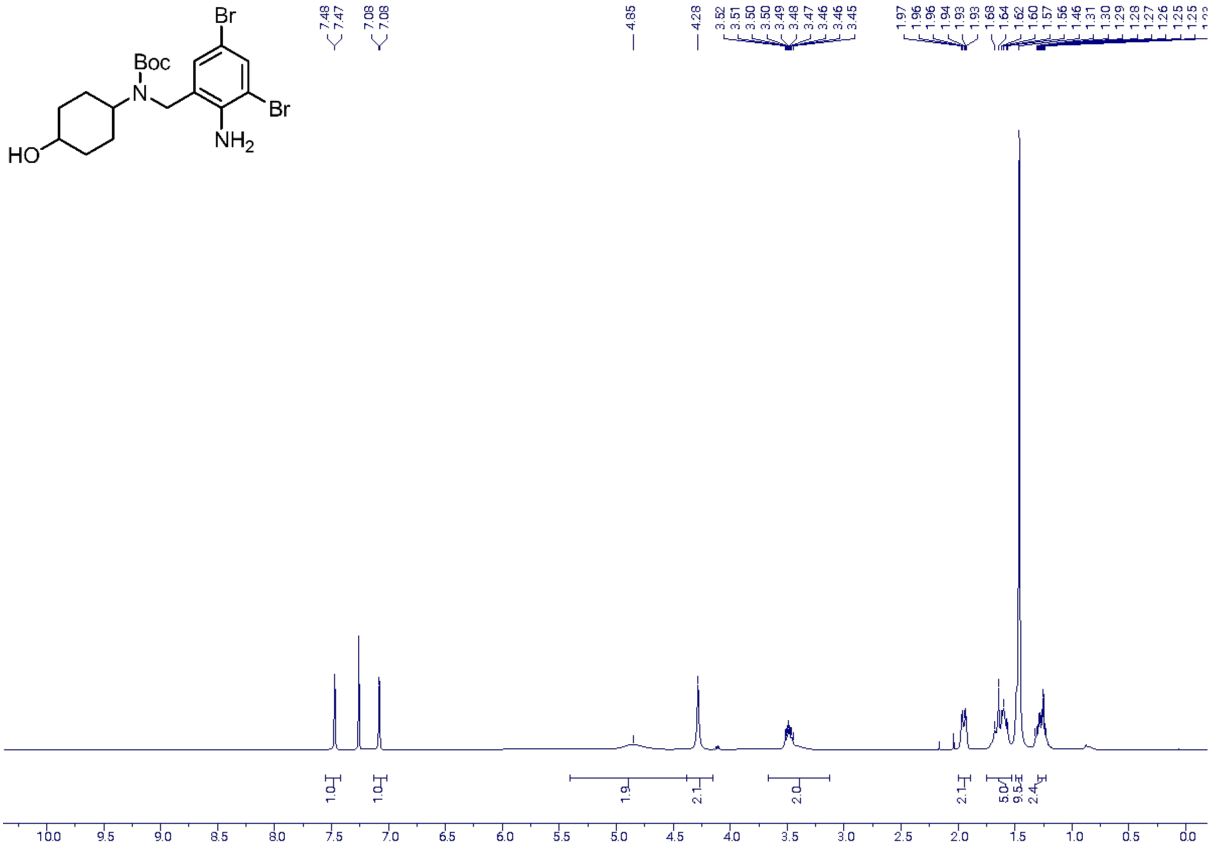

S1 – ¹³C NMR (101 MHz, CDCl₃)

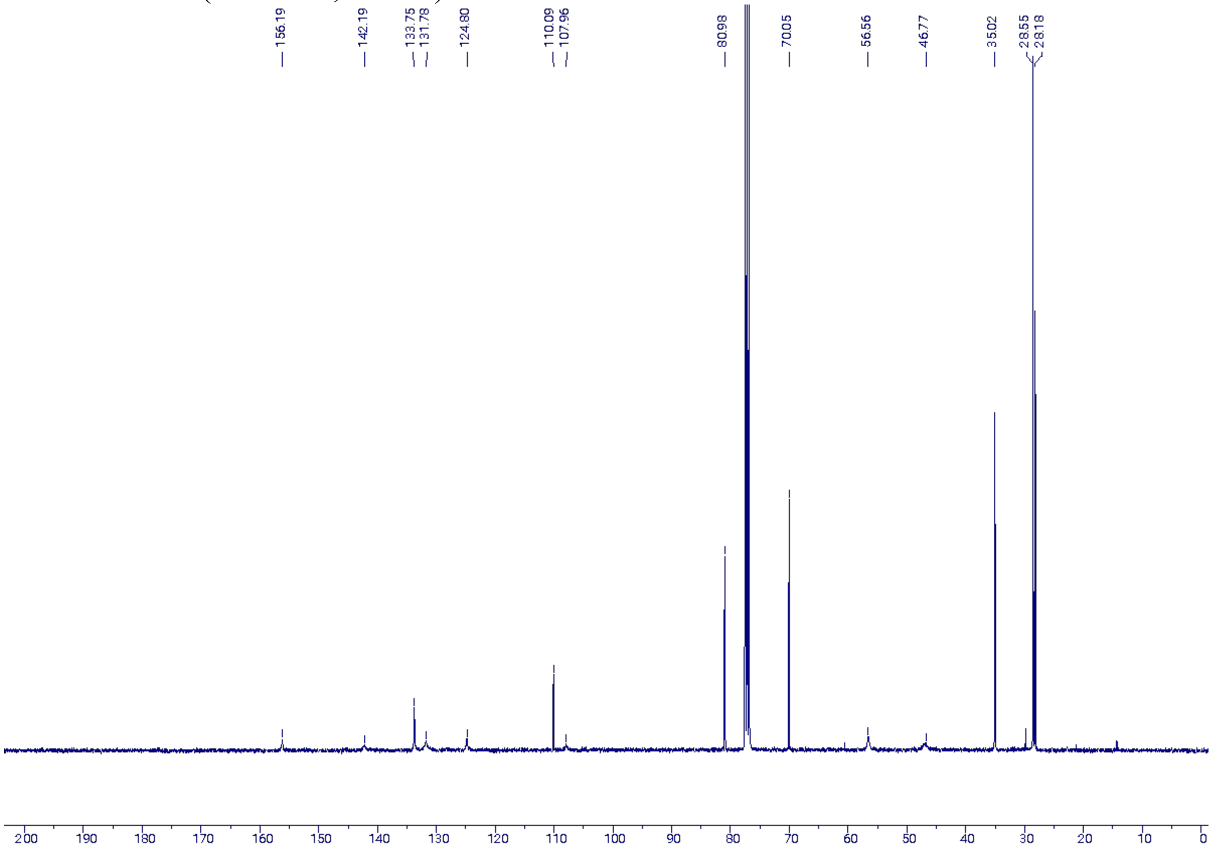

S11 – ^{1}H NMR (400 MHz, DMSO-*d*$_6$)

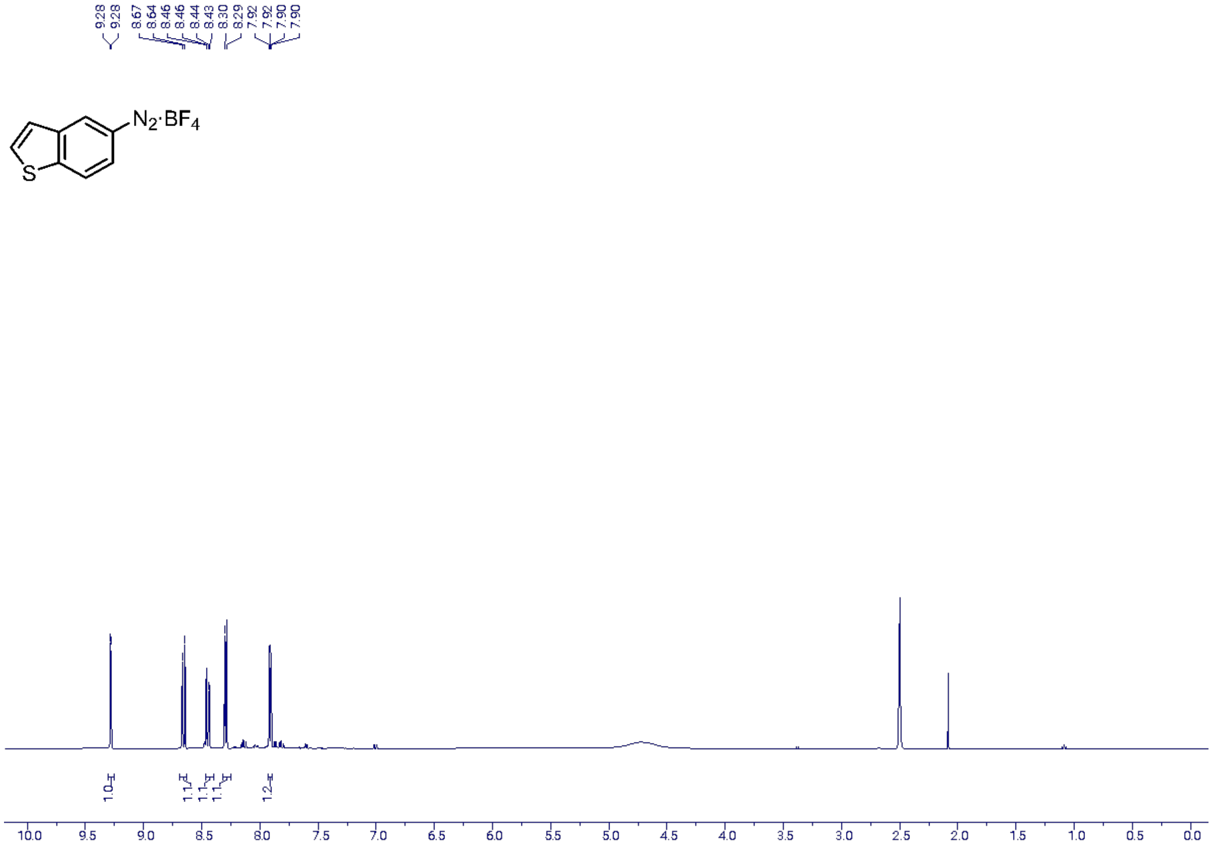

S11 – ^{13}C NMR (101 MHz, DMSO-*d*$_6$)

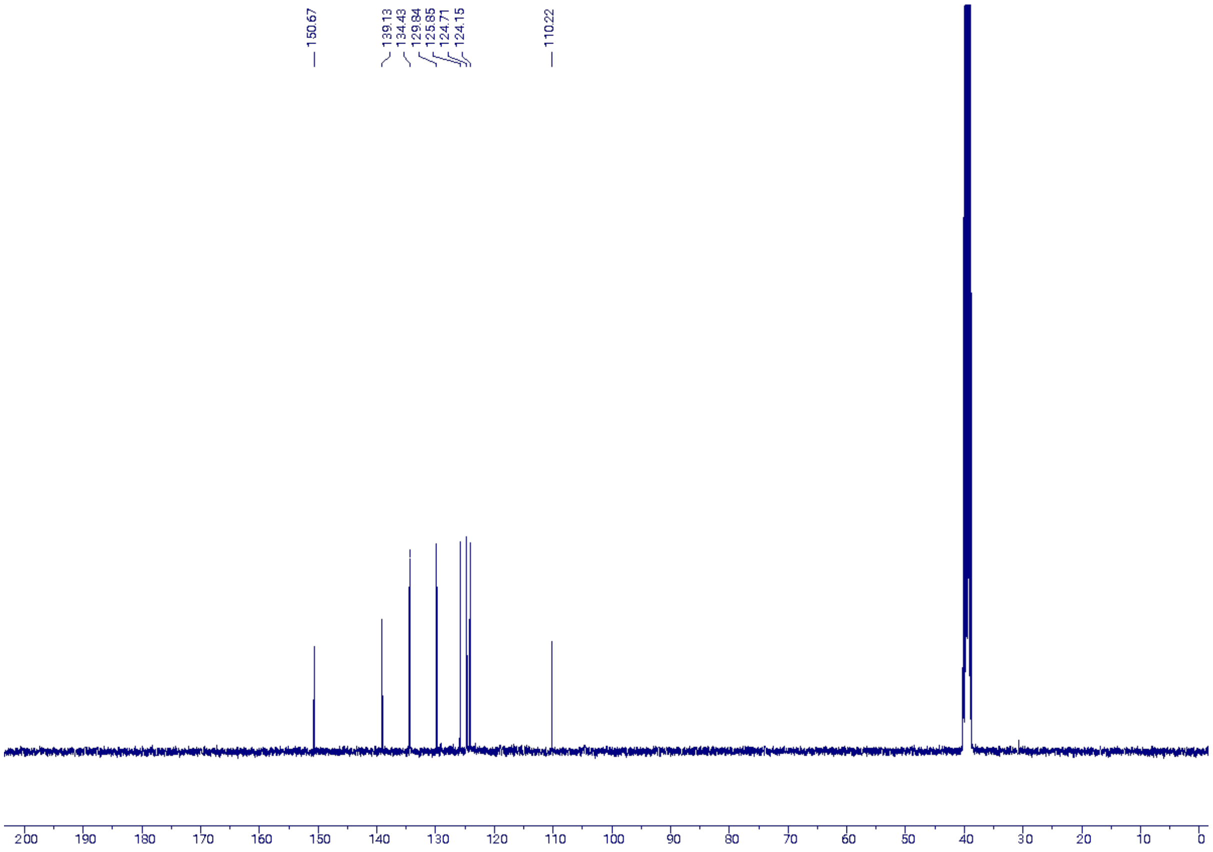

12 – ^{1}H NMR (400 MHz, $CDCl_3$)

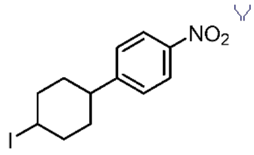

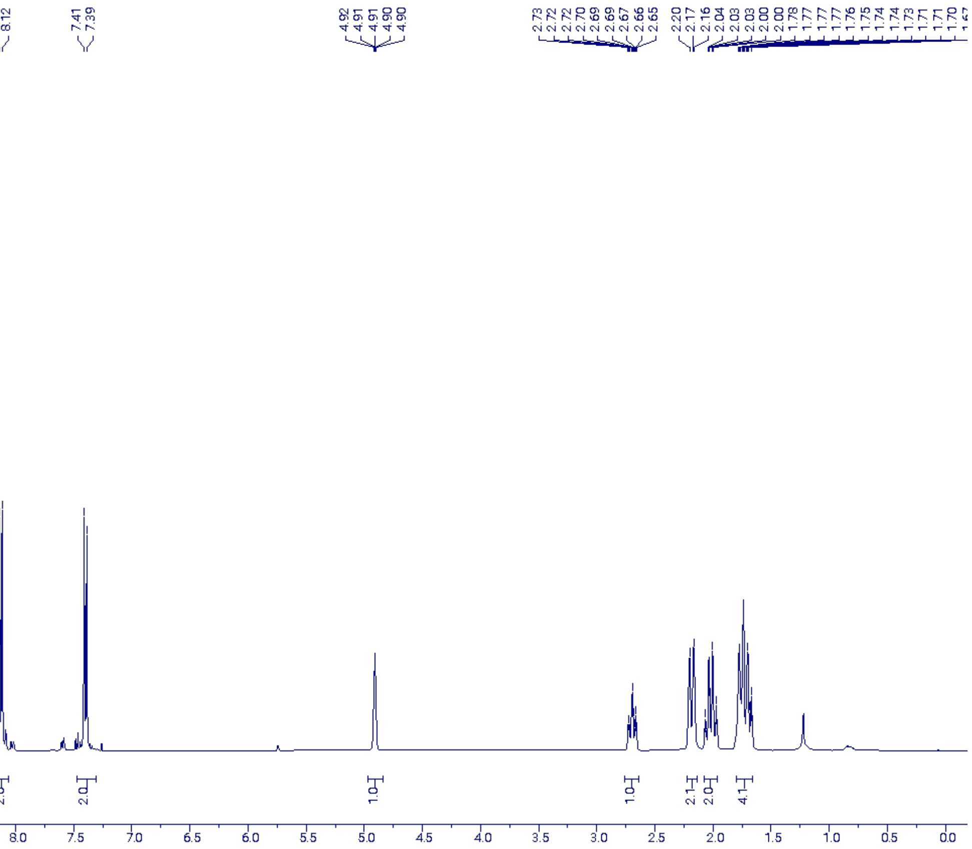

12 –^{13}C NMR (101 MHz, $CDCl_3$)

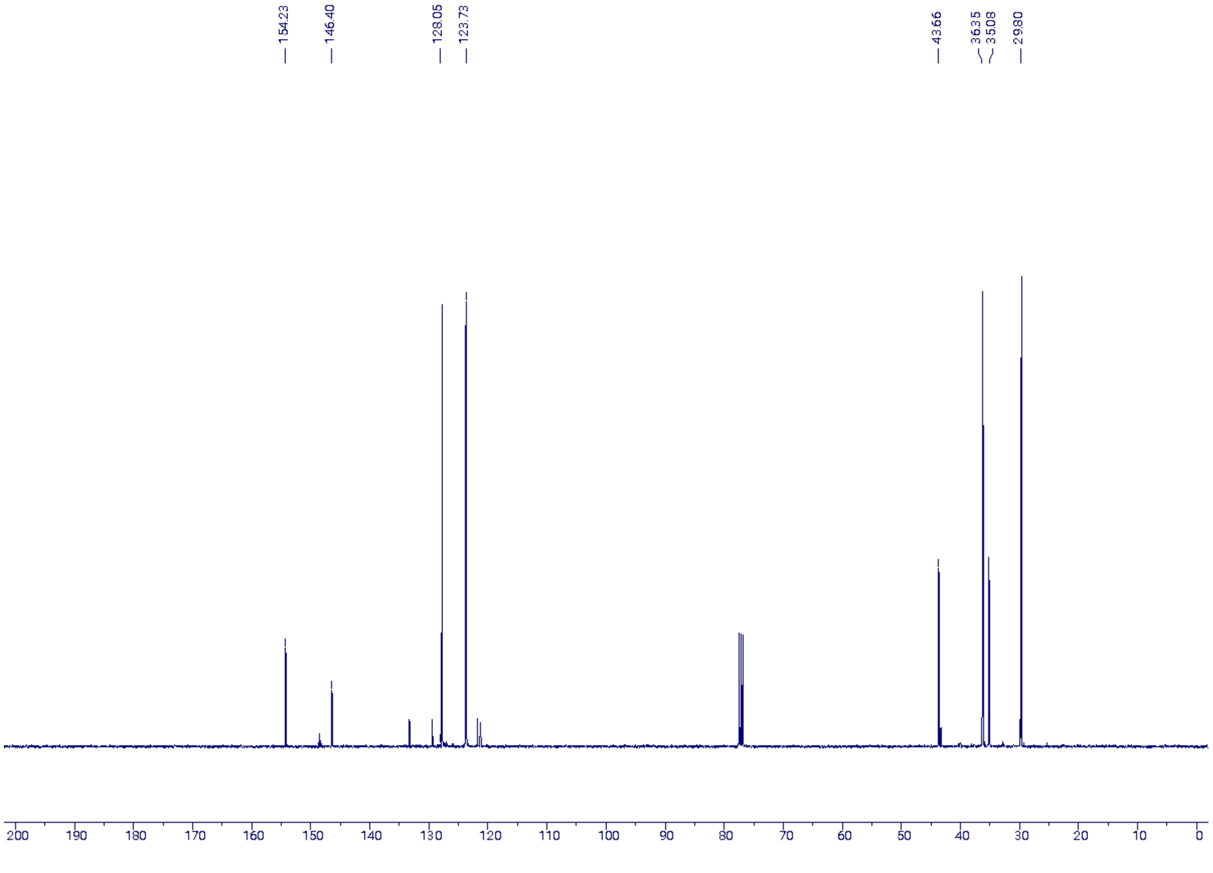

22 – ^{1}H NMR (400 MHz, $CDCl_3$, *cis/trans* mixture)

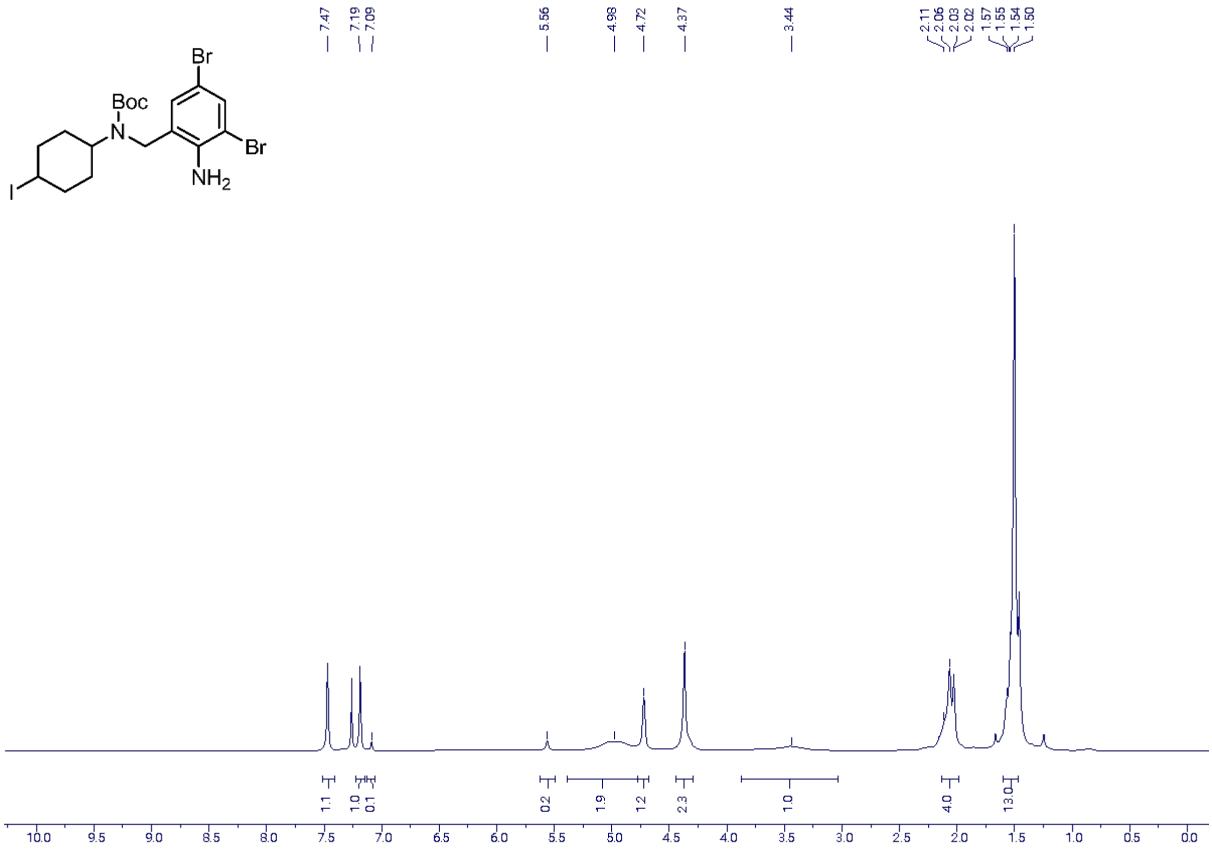

22 – ^{13}C NMR (101 MHz, $CDCl_3$, *cis/trans* mixture)

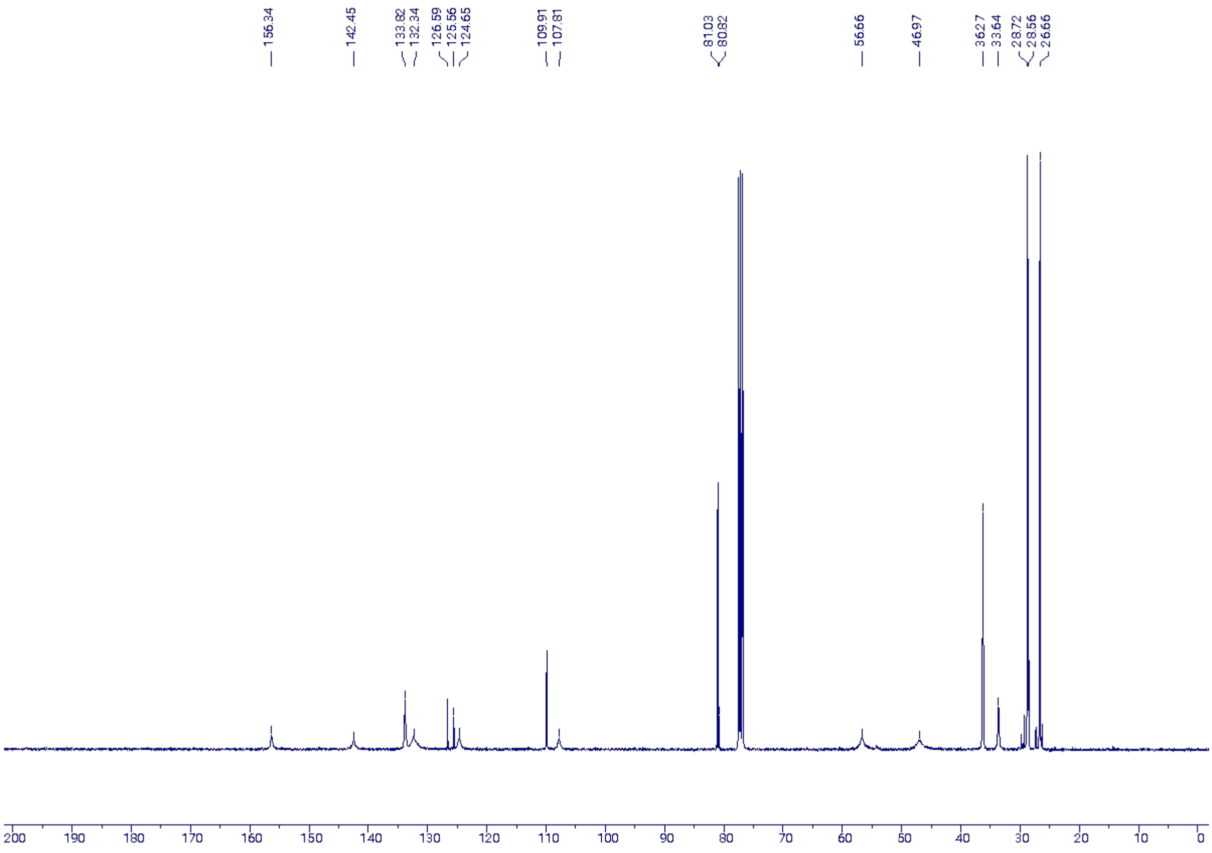

4 – ^{1}H NMR (400 MHz, CD_3CN)

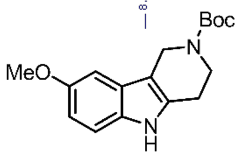

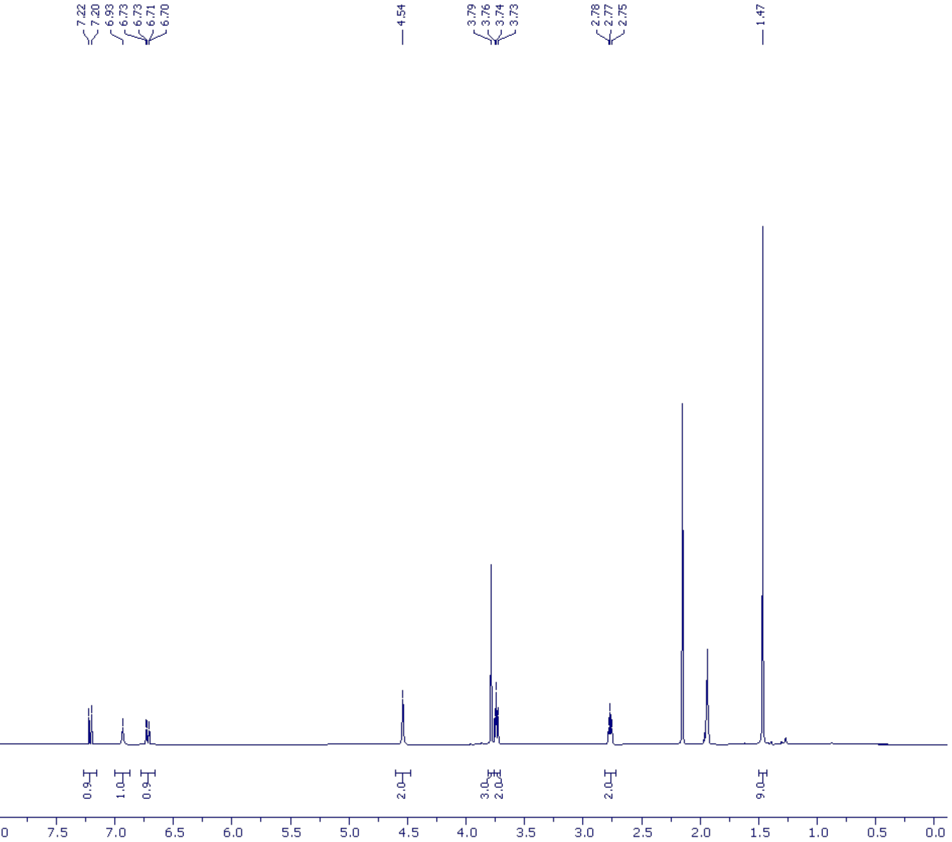

4 – ^{13}C NMR (101 MHz, CD_3CN)

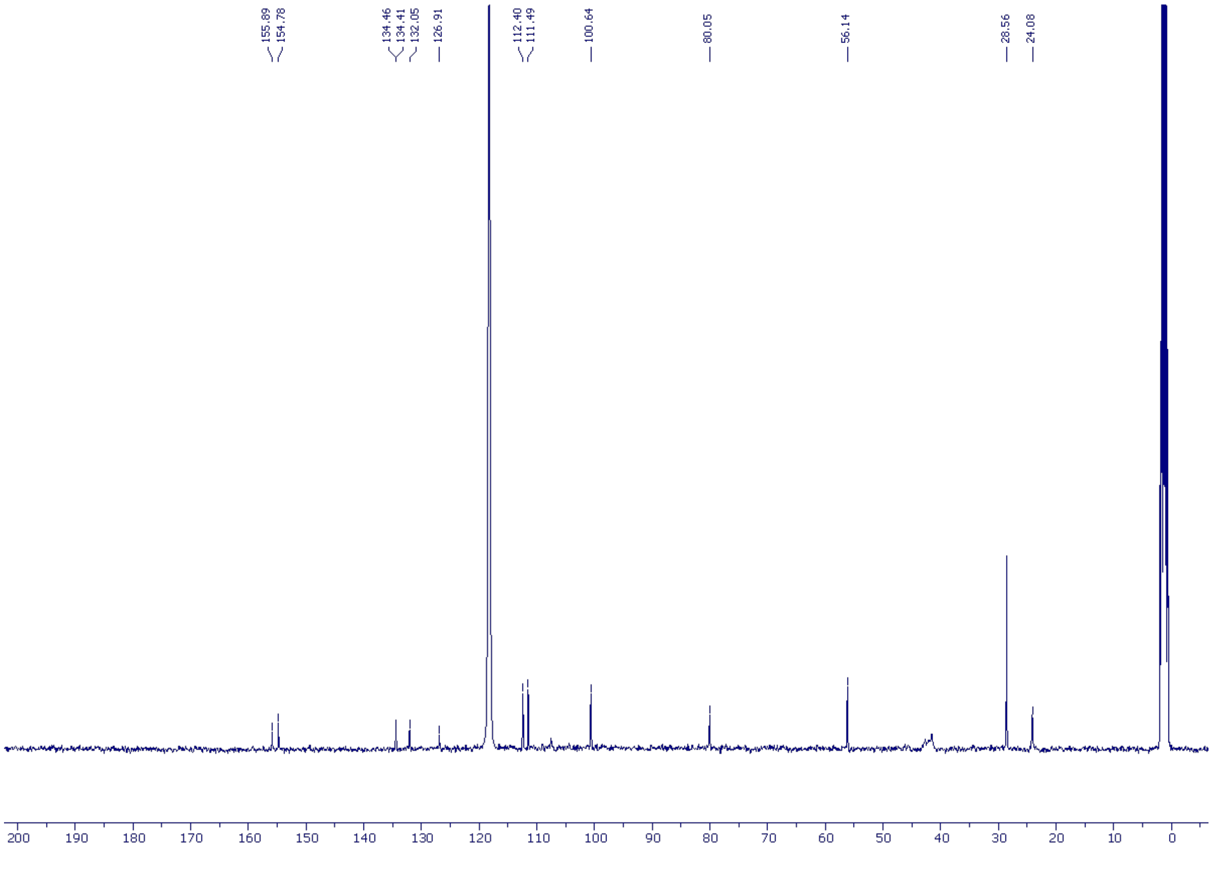

27 – ^{1}H NMR (400 MHz, CDCl$_3$)

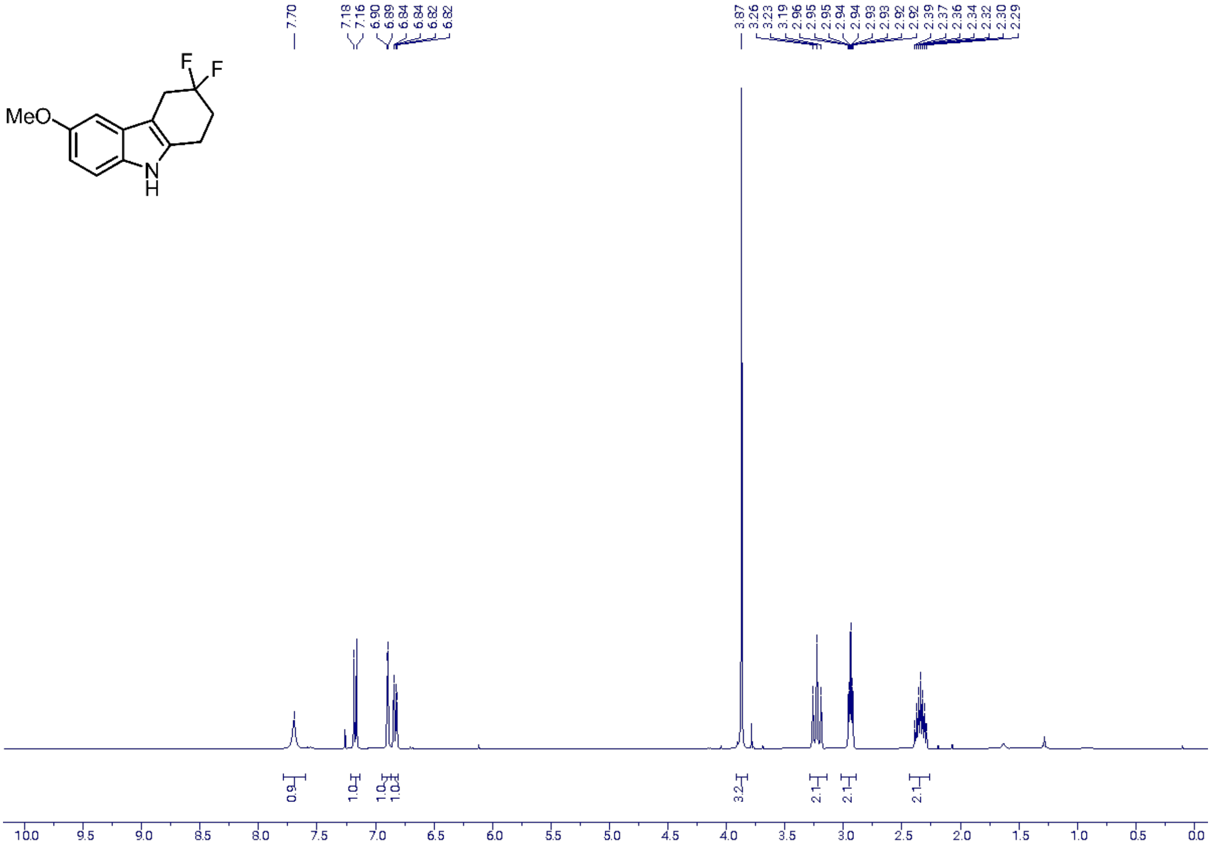

27 – ^{13}C NMR (101 MHz, CDCl$_3$)

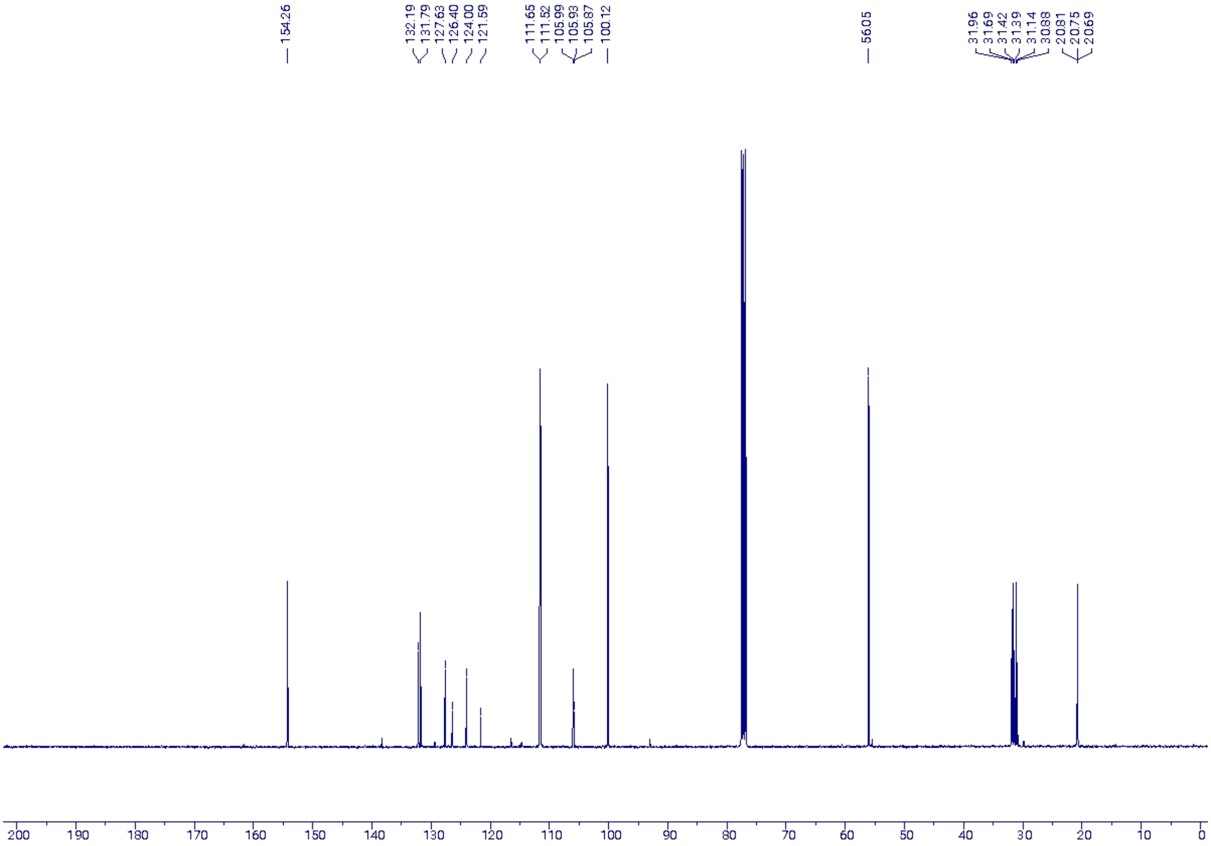

27 – ^{19}F NMR (376 MHz, $CDCl_3$, $PhCF_3$ as reference)

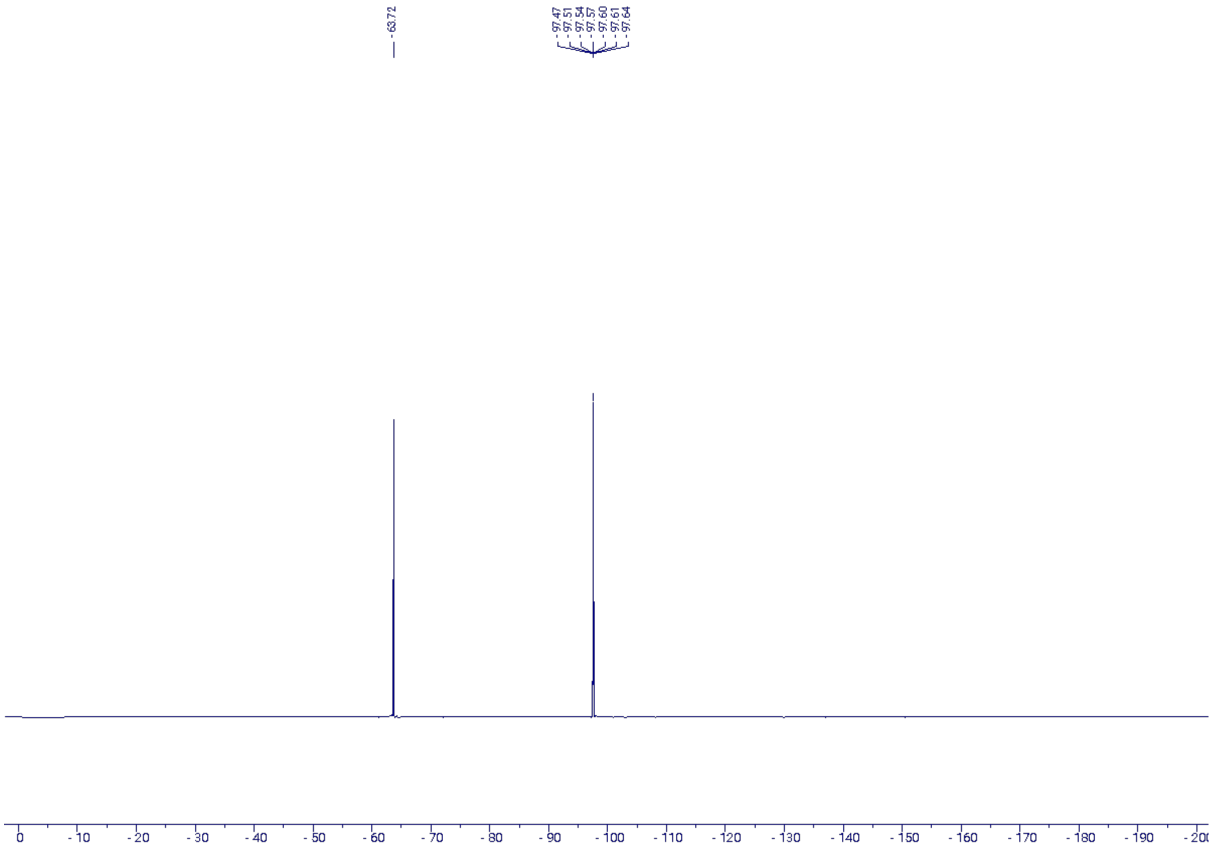

28 – ^{1}H NMR (400 MHz, $CDCl_3$)

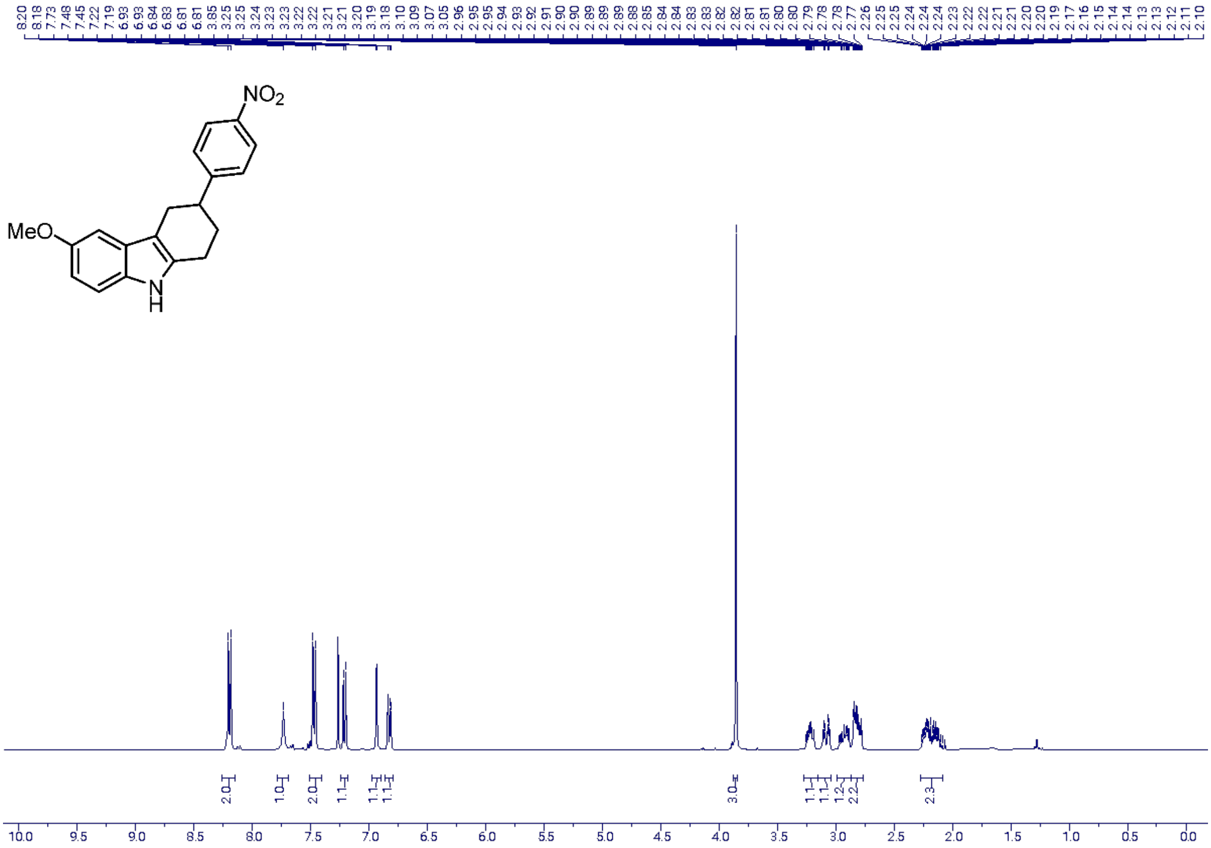

28 – ^{13}C NMR (101 MHz, $CDCl_3$)

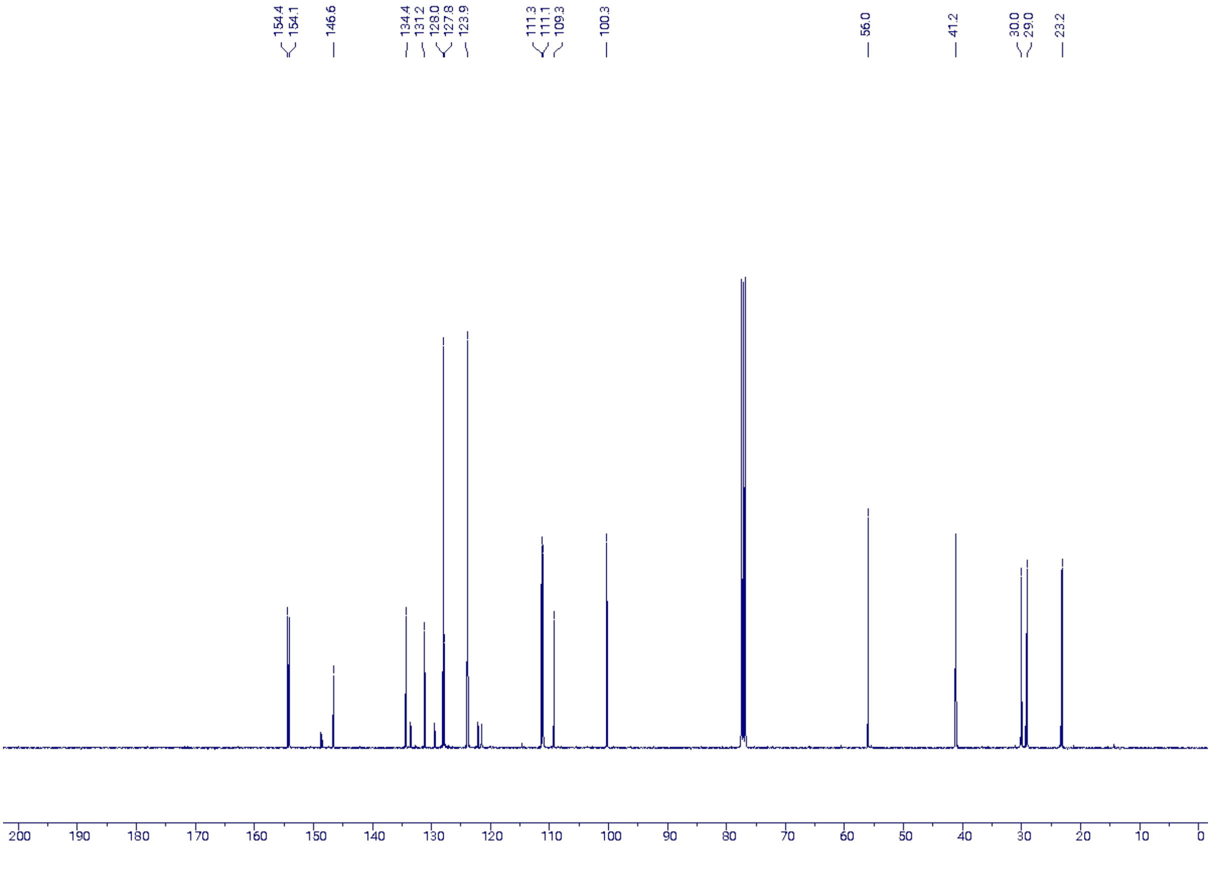

29 – ^{1}H NMR (400 MHz, $CDCl_3$)

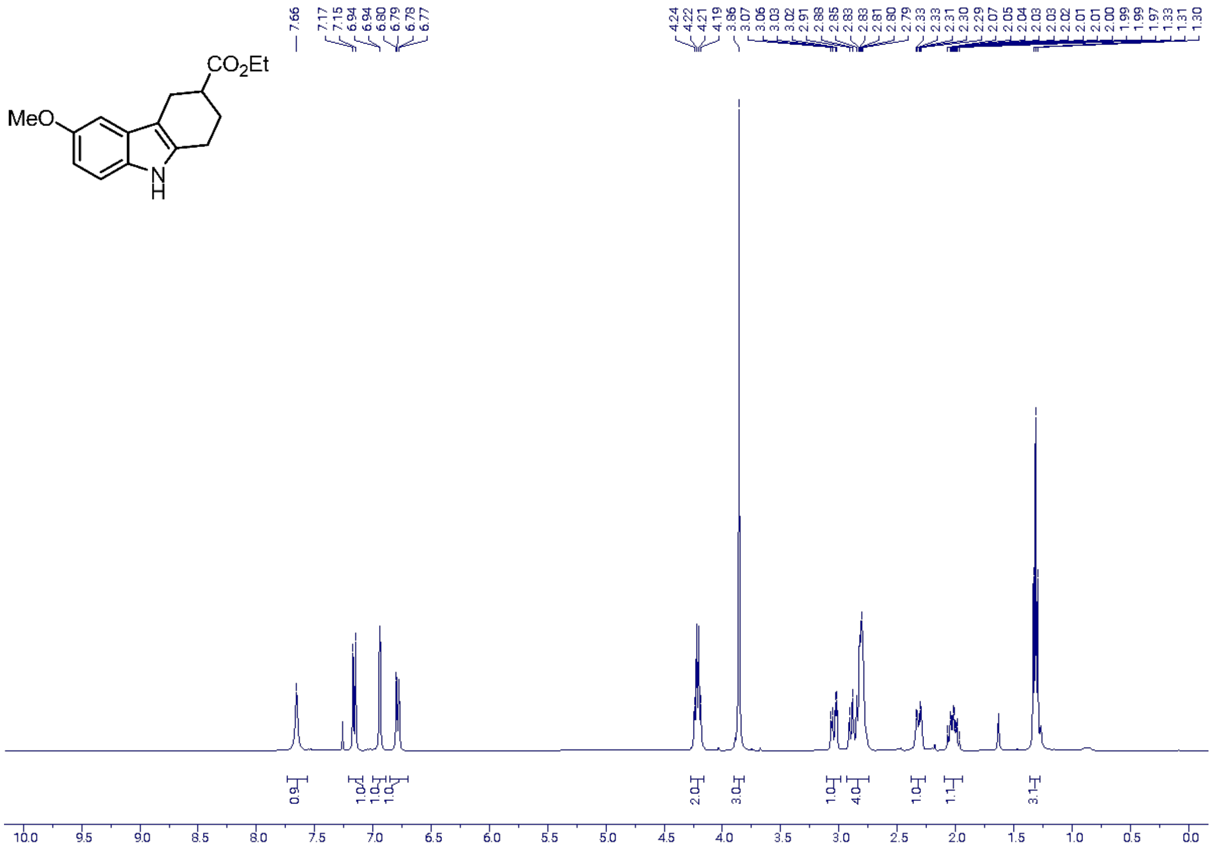

29 – ^{13}C NMR (101 MHz, $CDCl_3$)

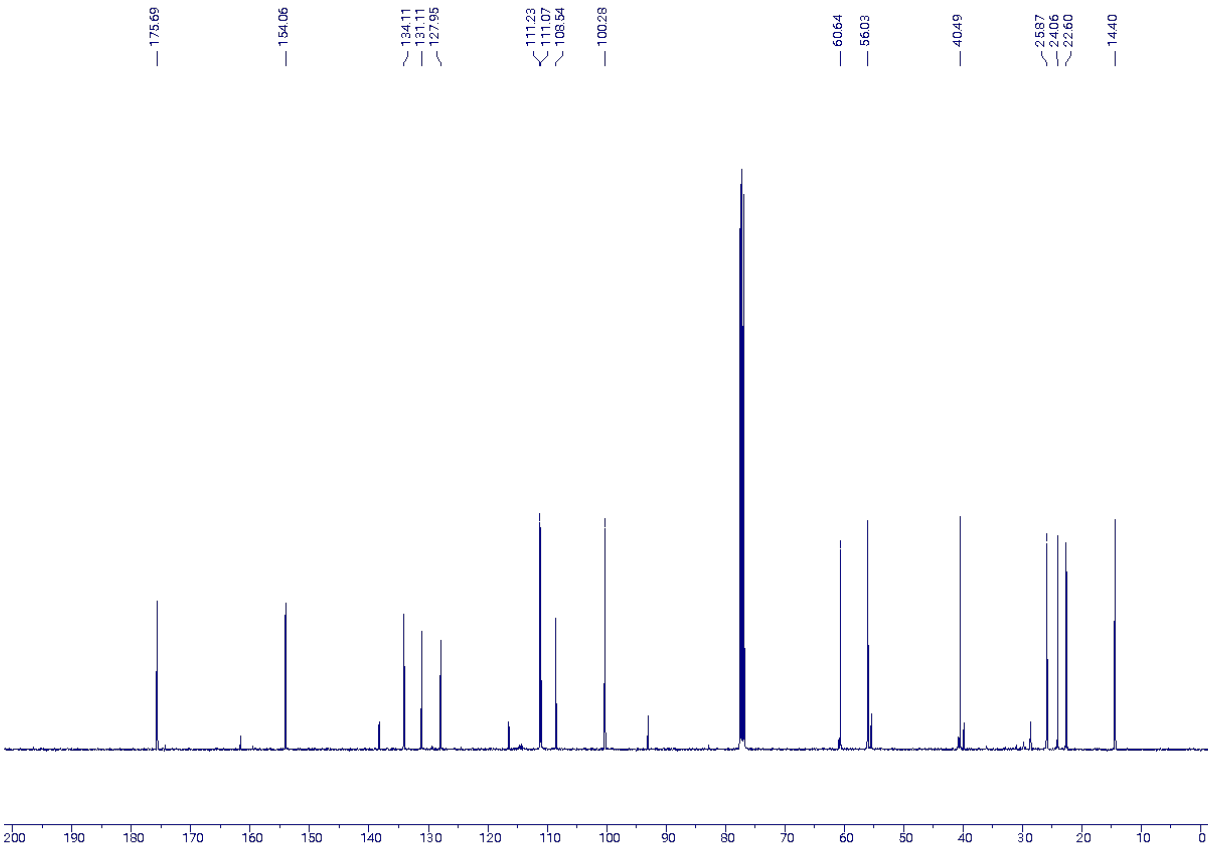

30 – ^{1}H NMR (400 MHz, $CDCl_3$)

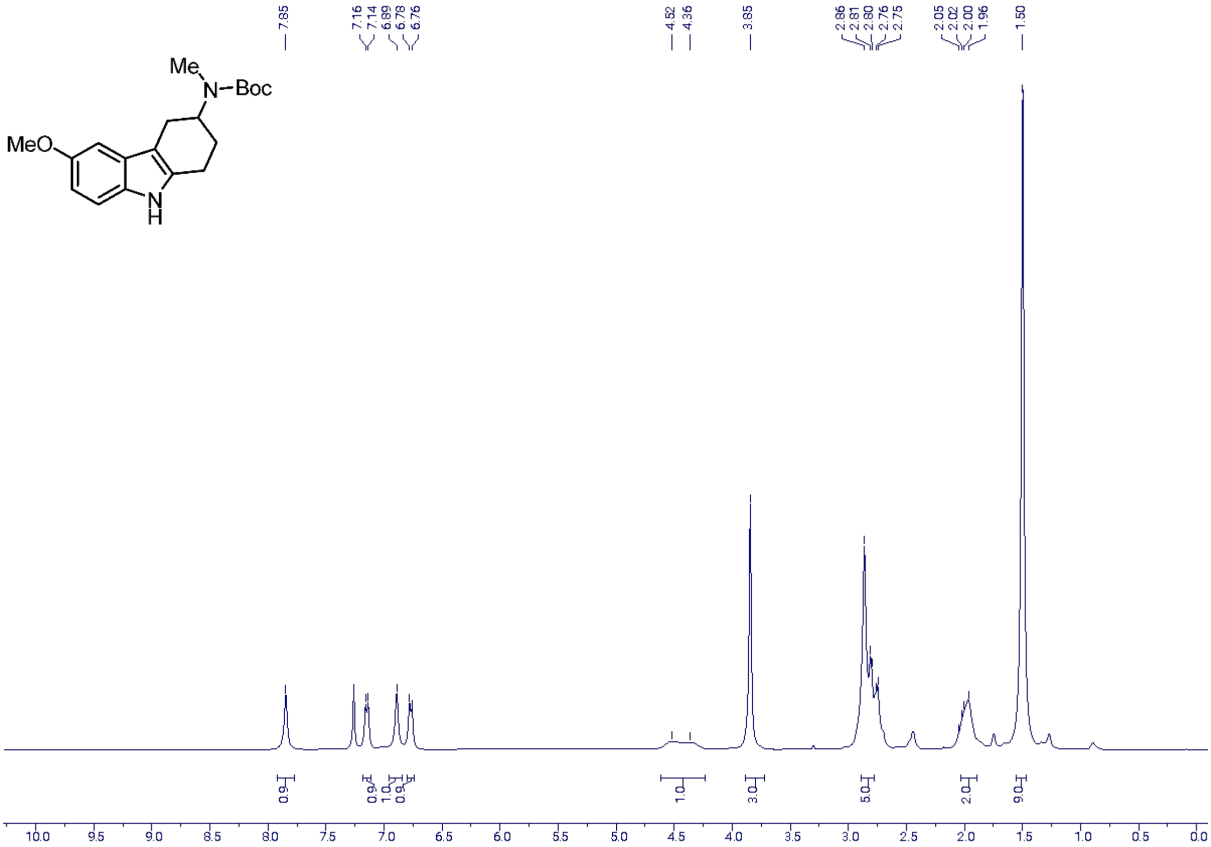

30 – ^{13}CNMR (101 MHz, $CDCl_3$)

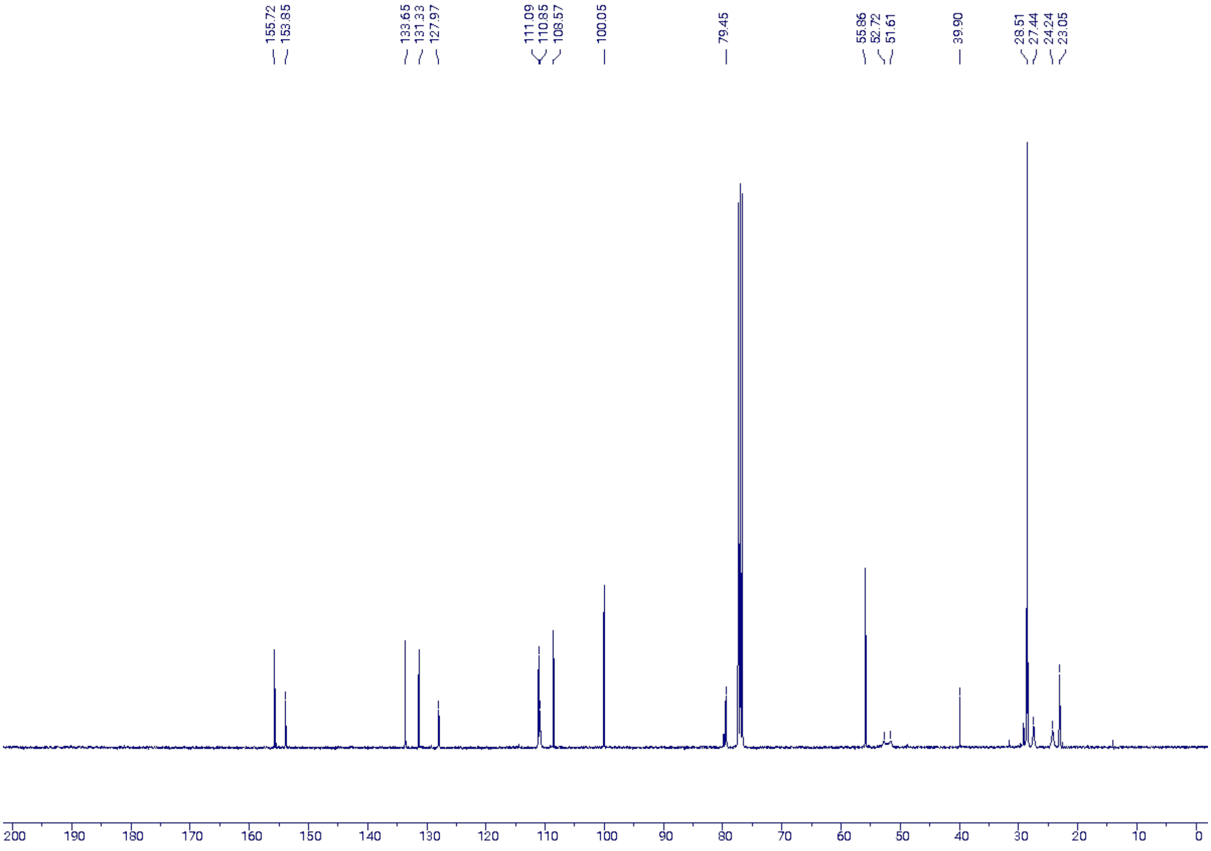

31 – ^{1}H NMR (400 MHz, $CDCl_3$)

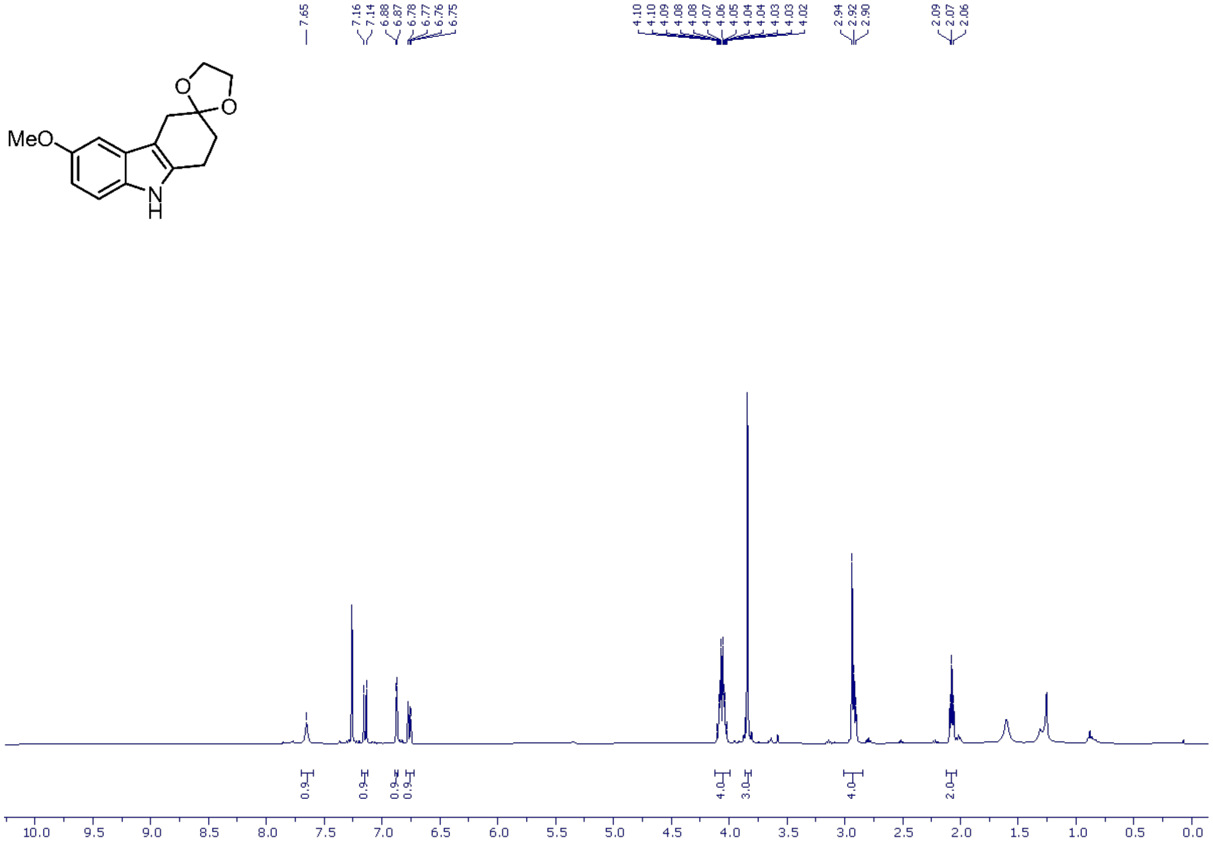

31 – ^{13}C NMR (101 MHz, $CDCl_3$)

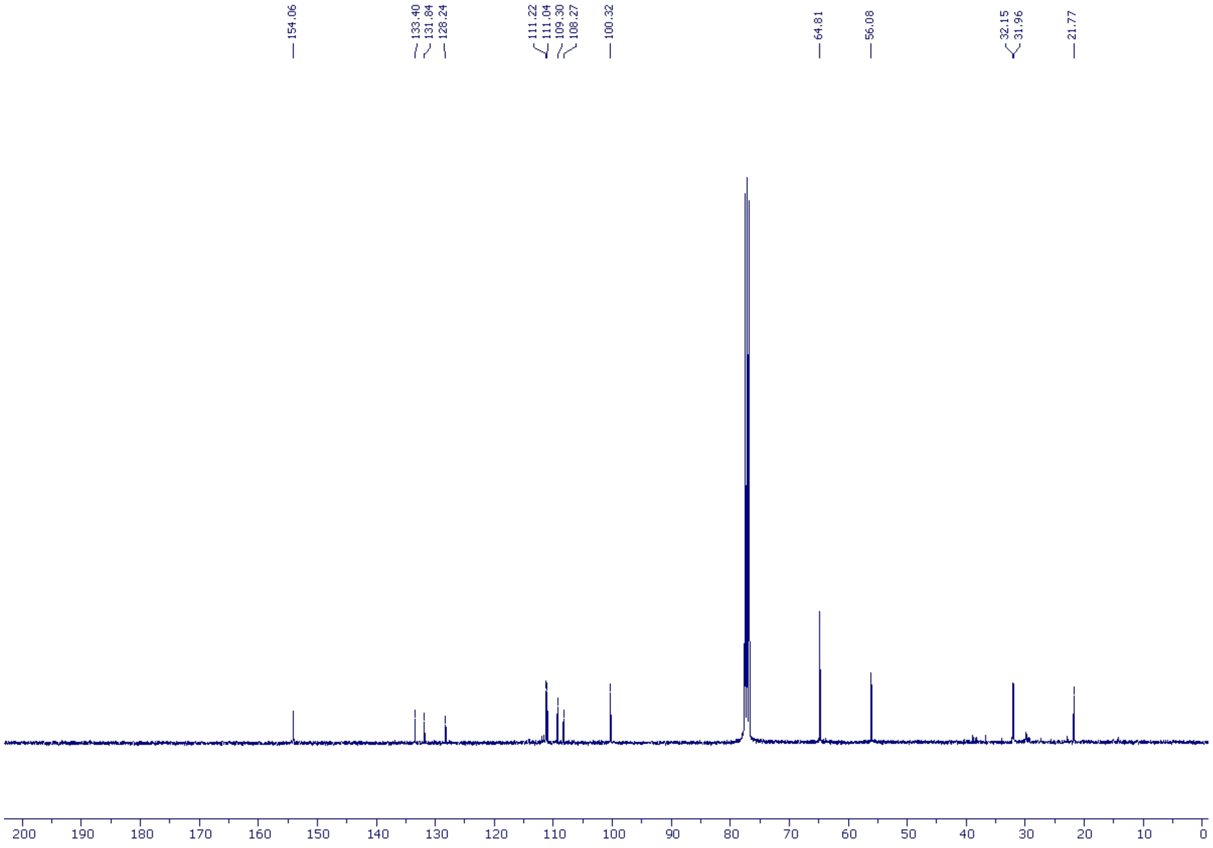

32 – ^{1}H NMR (500 MHz, CDCl$_3$)

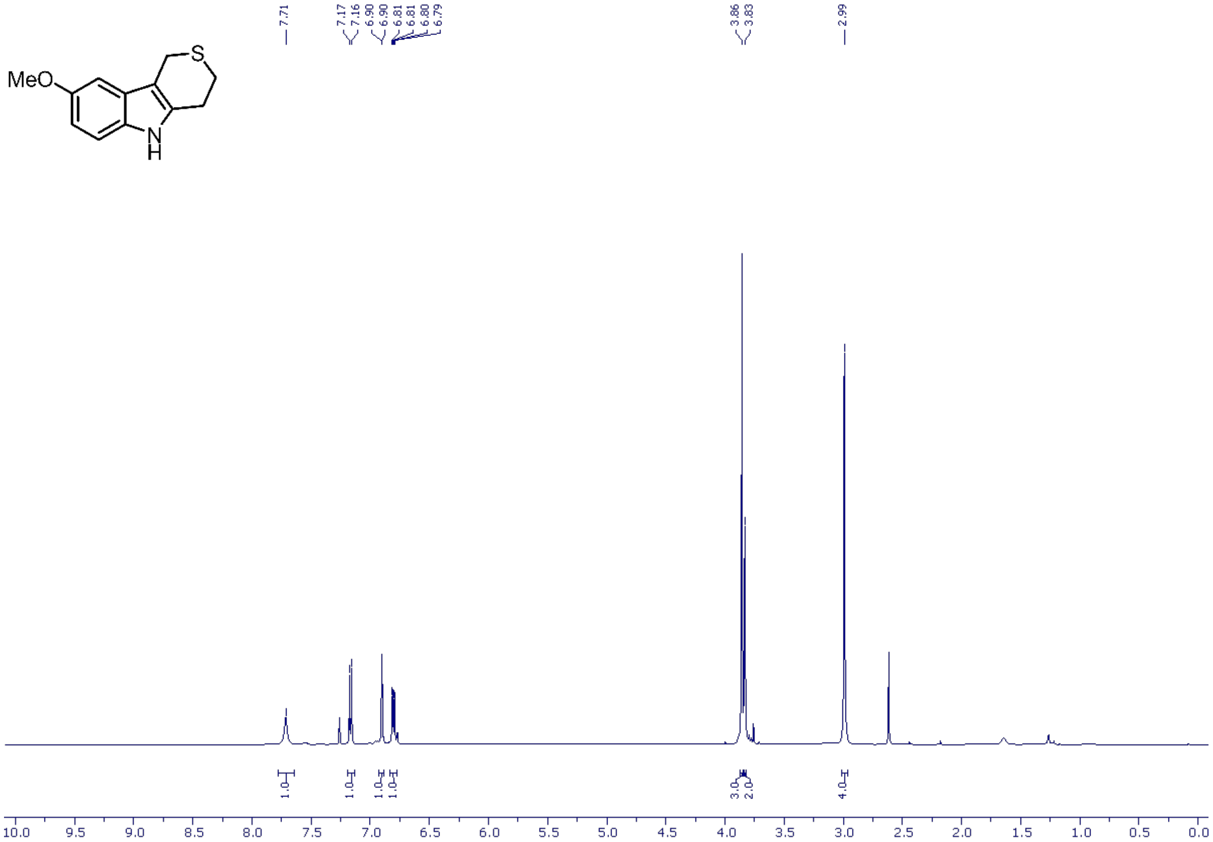

32 – ^{13}C NMR (126 MHz, CDCl$_3$)

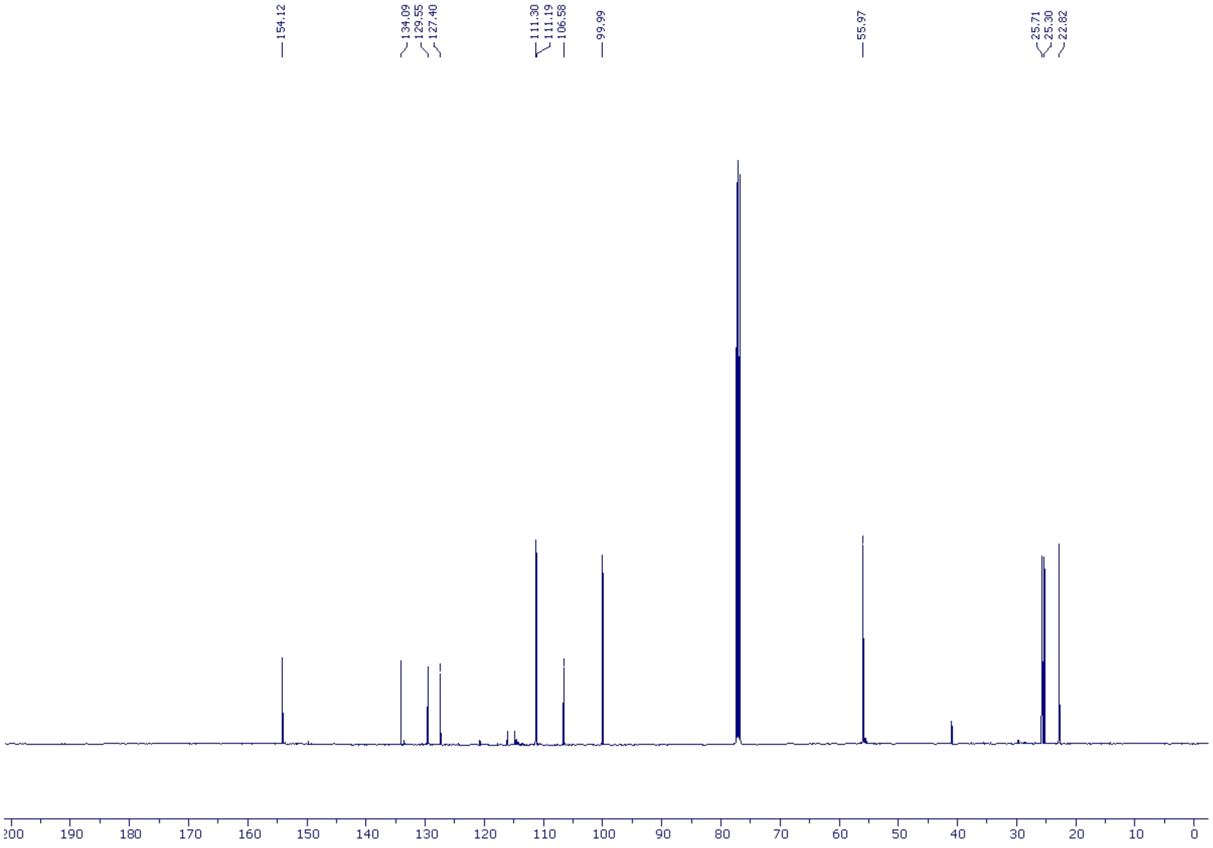

33 – ¹H NMR (400 MHz, $CDCl_3$)

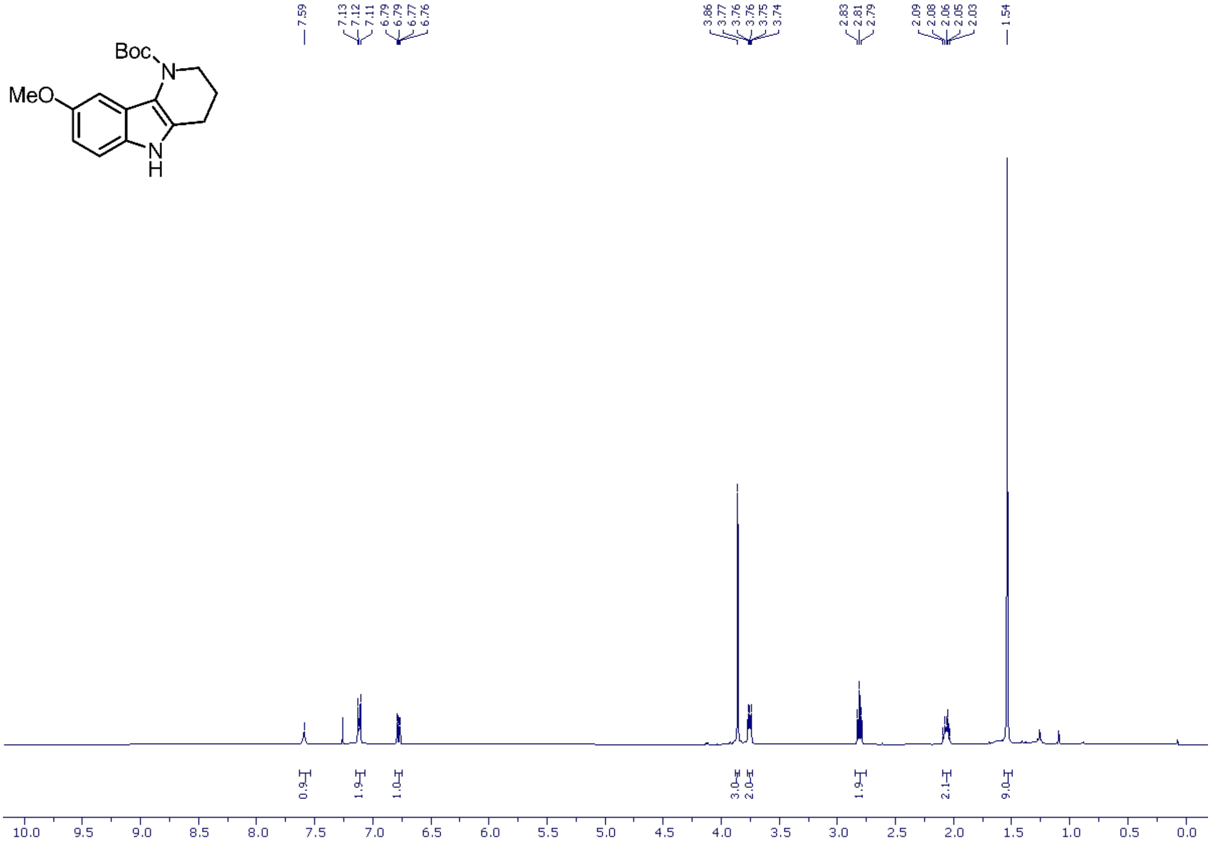

33 – ^{13}C NMR (101 MHz, $CDCl_3$)

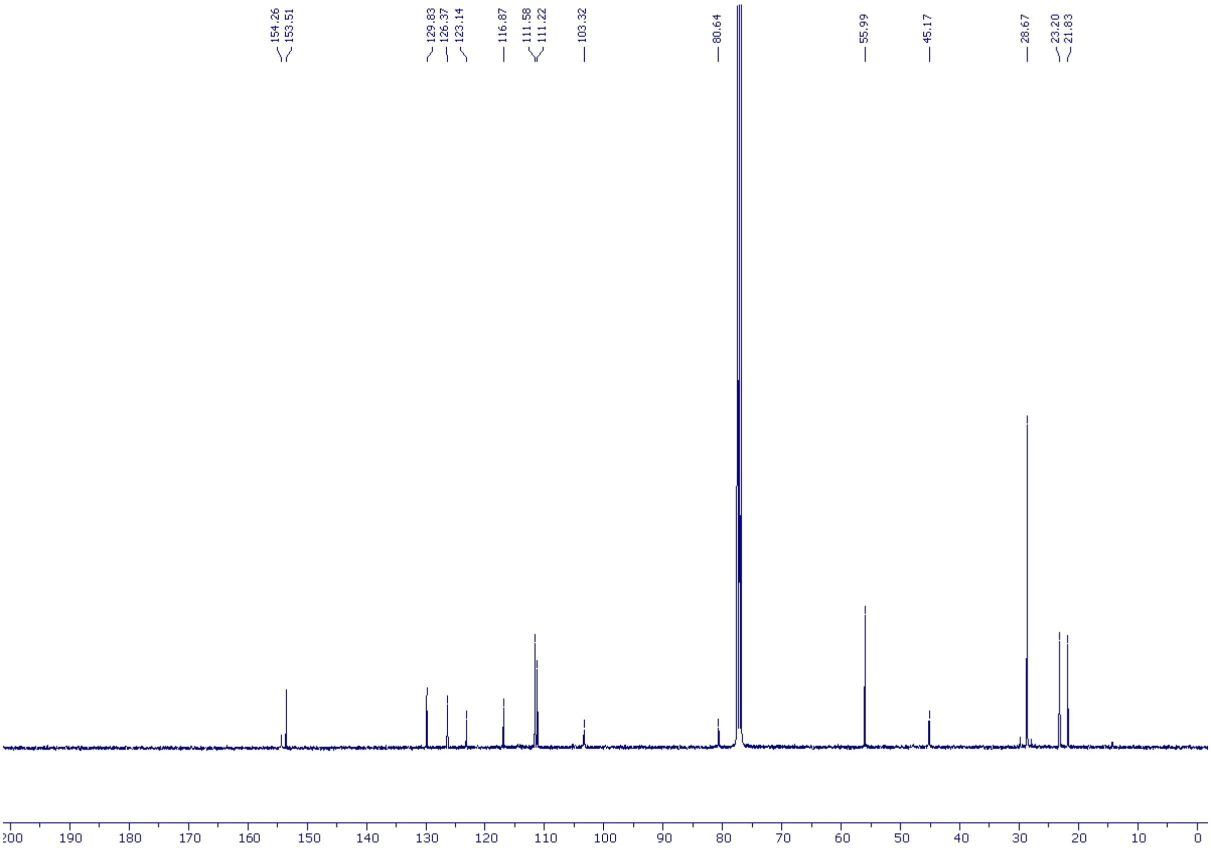

34 – ^{1}H NMR (400 MHz, $CDCl_3$, rotamers)

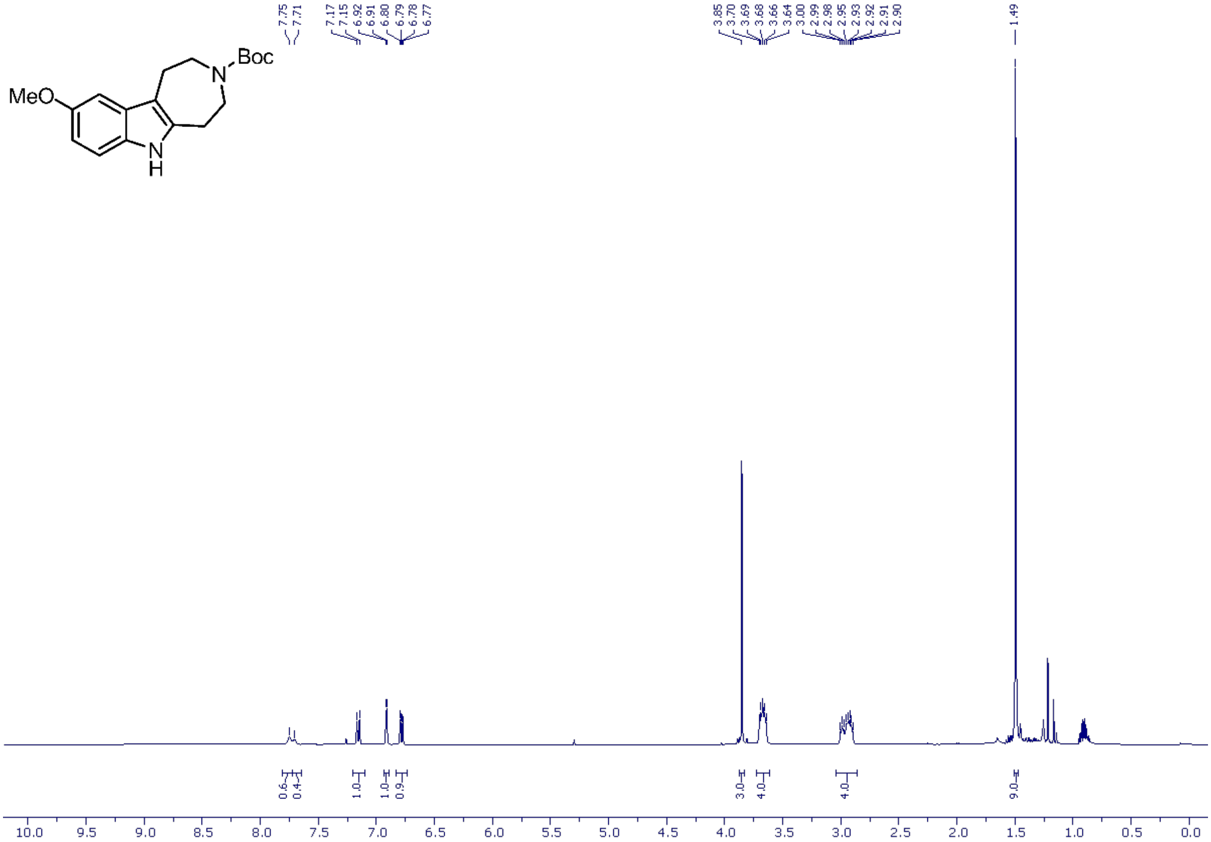

34 – ^{13}C NMR (101 MHz, $CDCl_3$, rotamers)

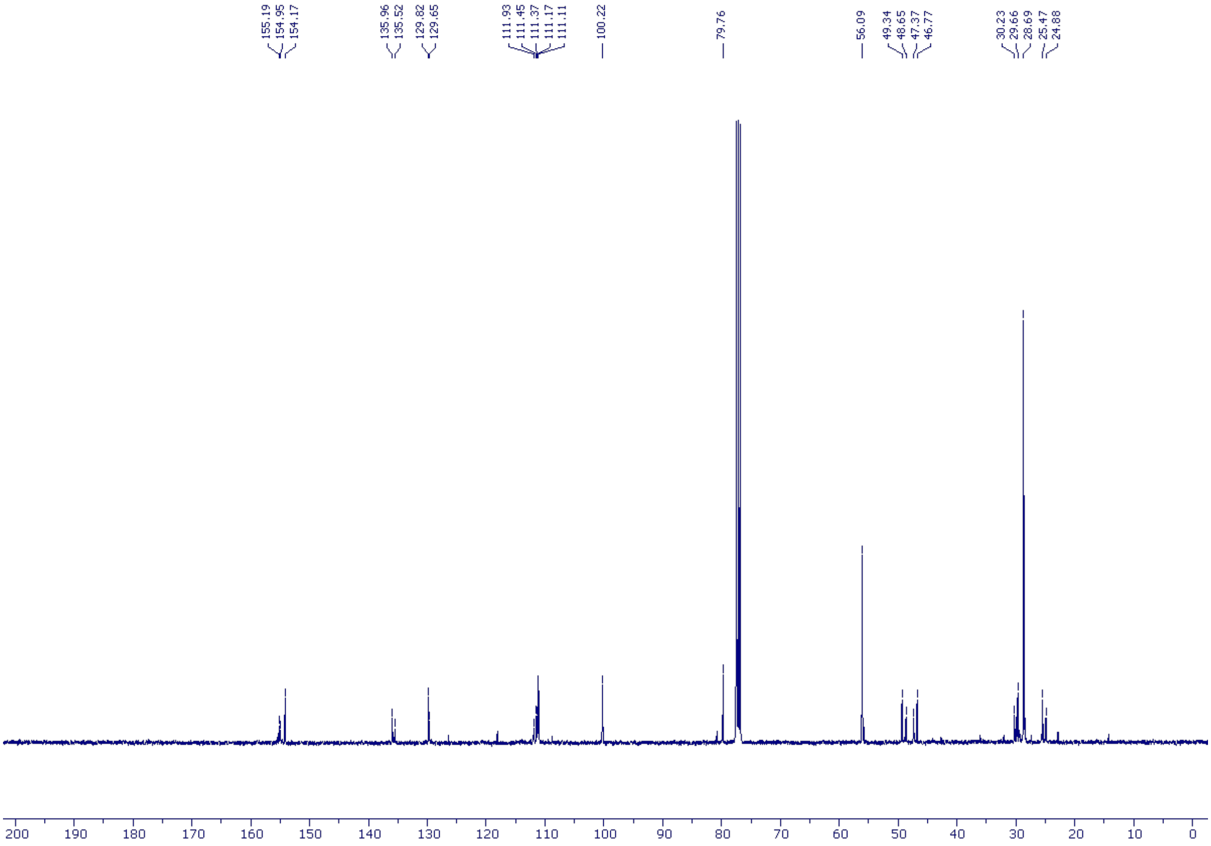

35 – ^{1}H NMR (400 MHz, THF-d_8)

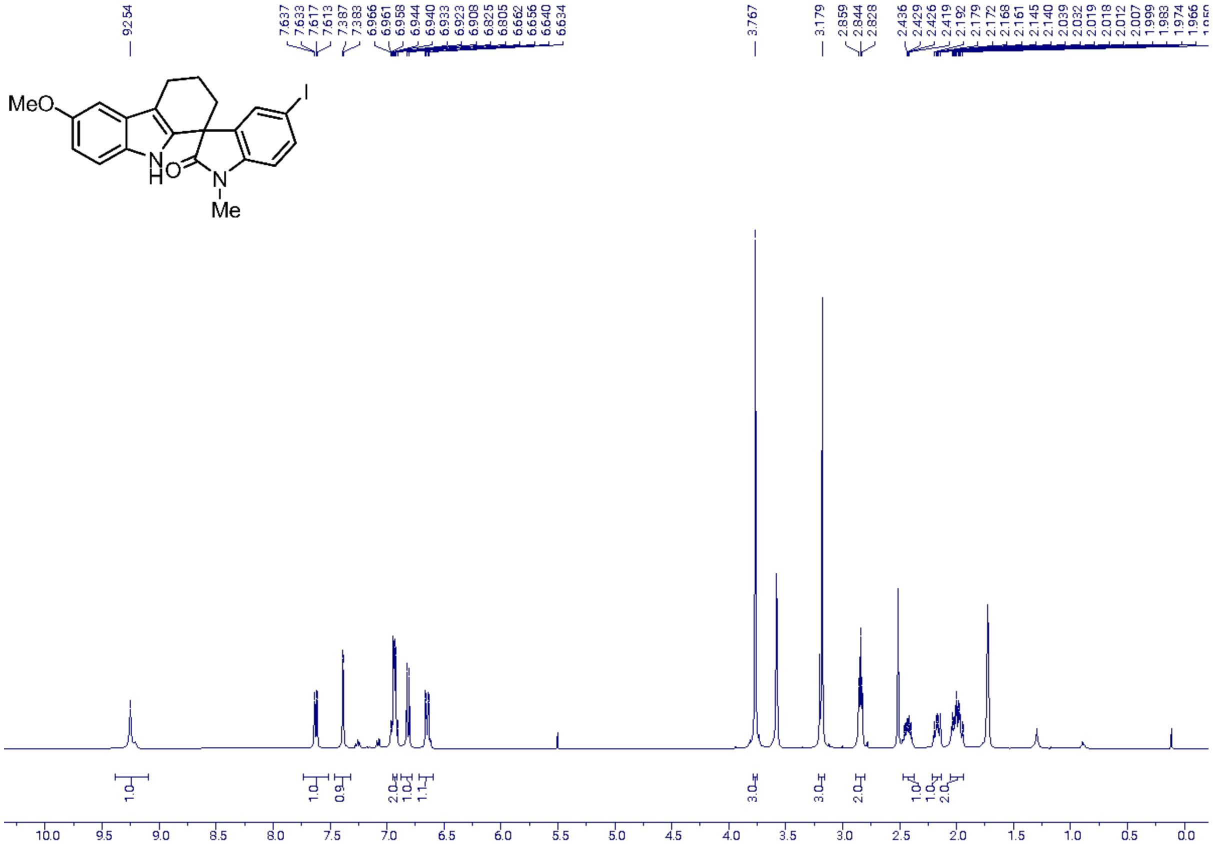

35 – ^{13}C NMR (101 MHz, THF-d_8)

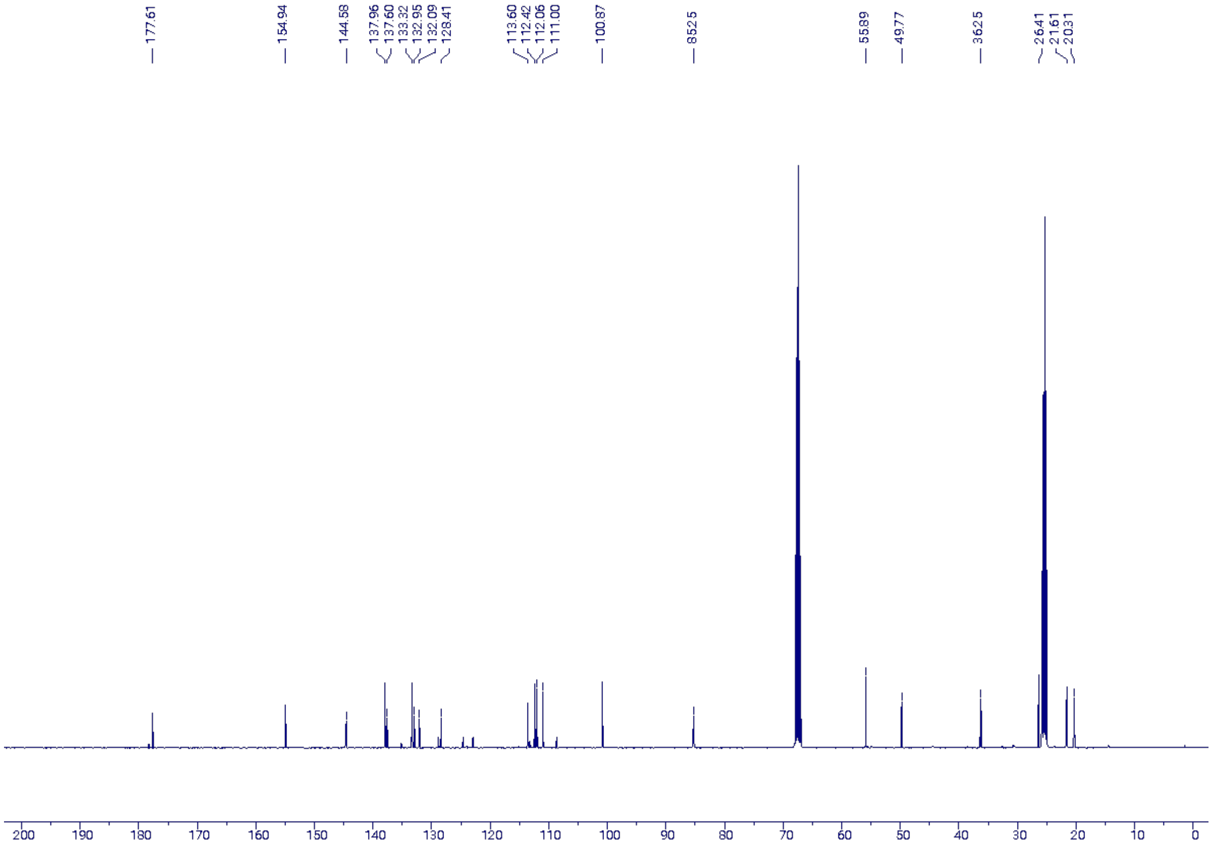

36 – ^{1}H NMR (400 MHz, $CDCl_3$)

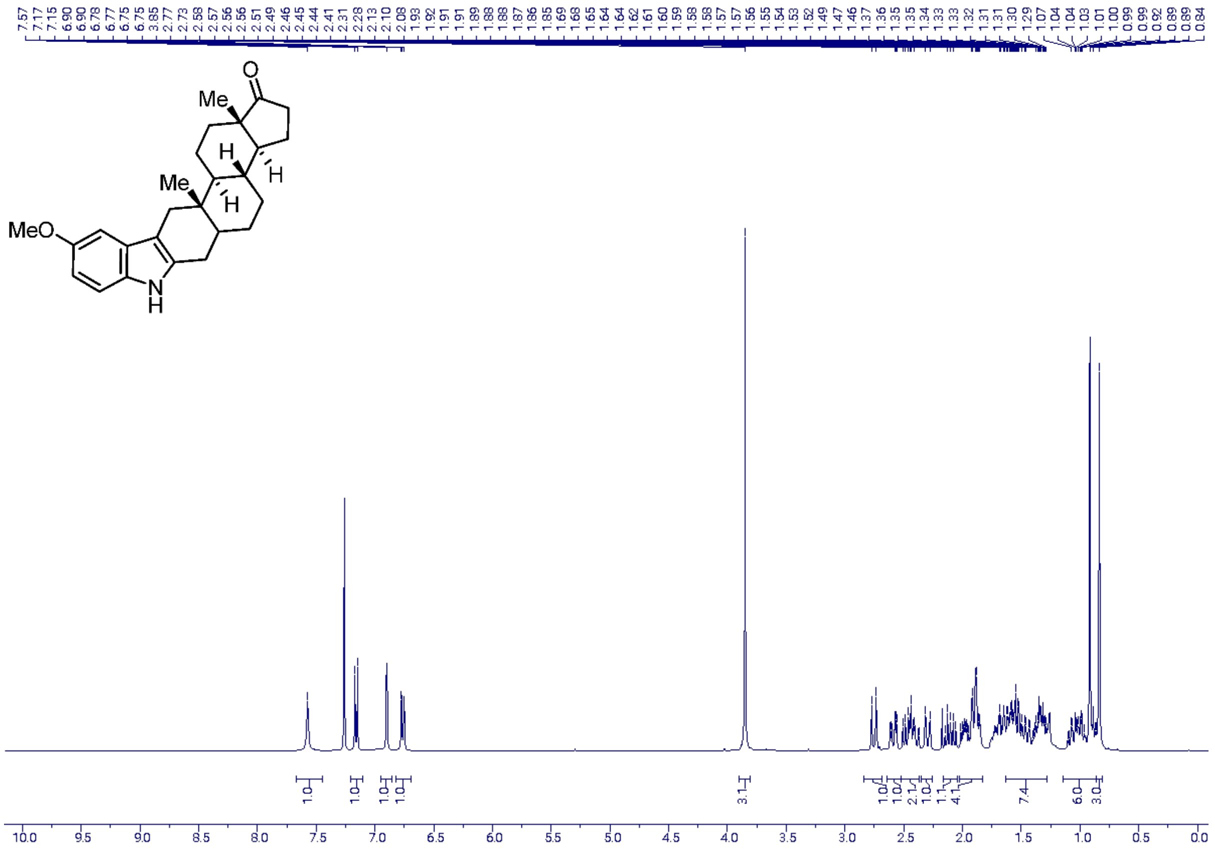

36 – ^{13}C NMR (101 MHz, $CDCl_3$)

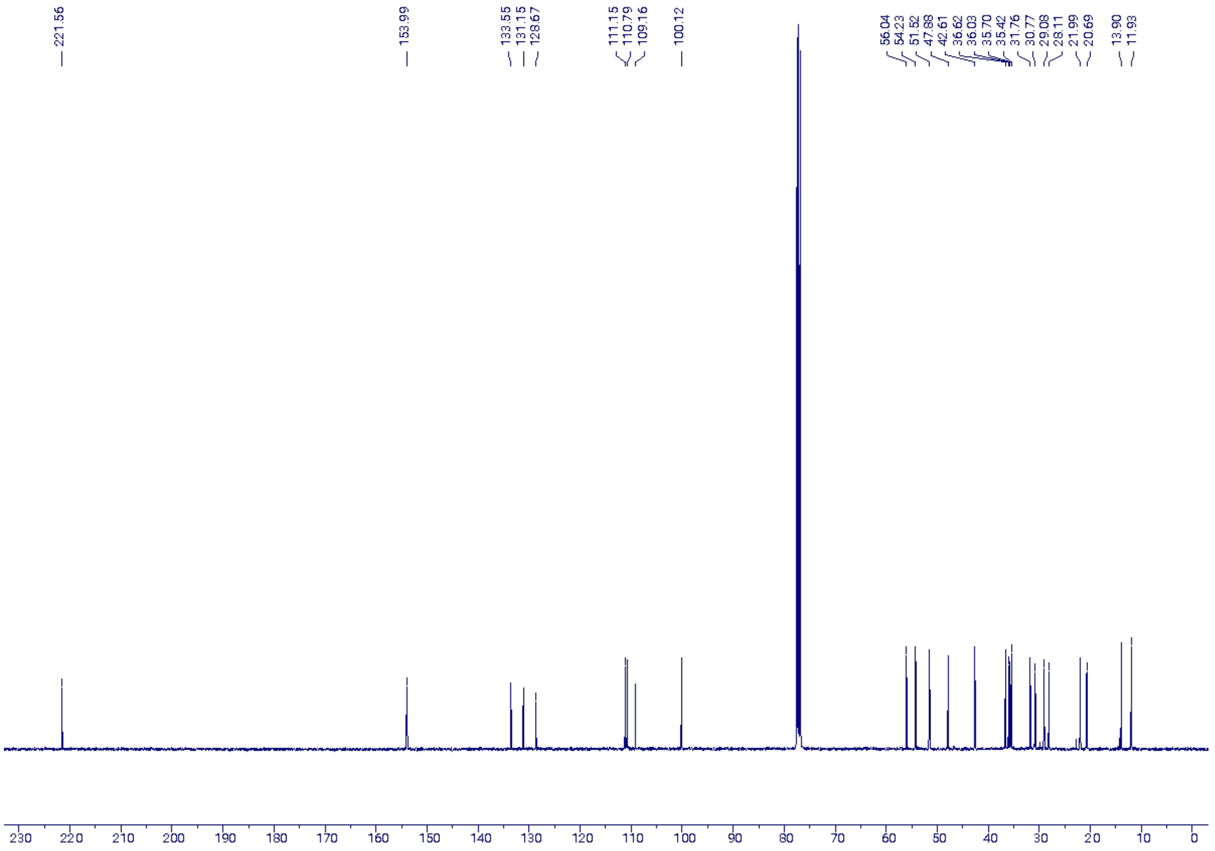

37 – ^{1}H NMR (400 MHz, THF-d_8)

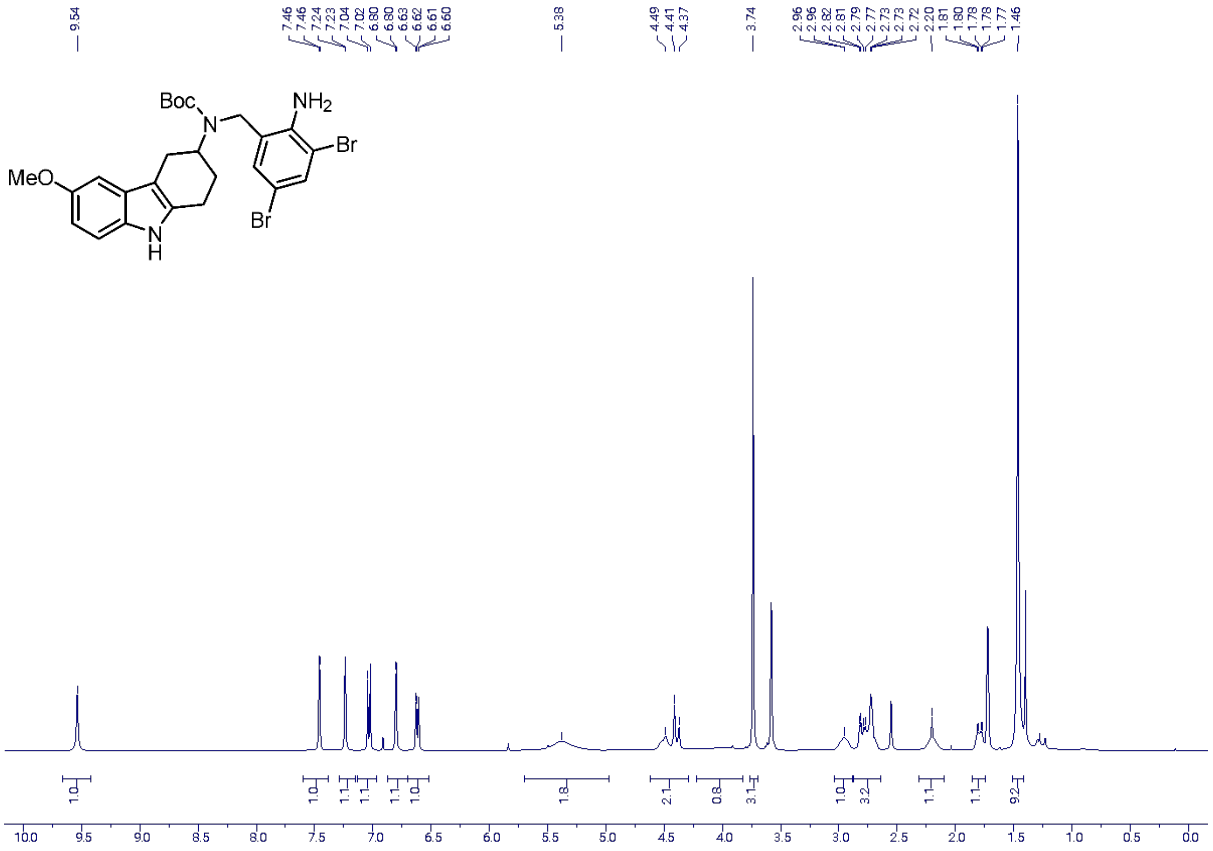

37 – ^{13}C NMR (101 MHz, THF-d_8)

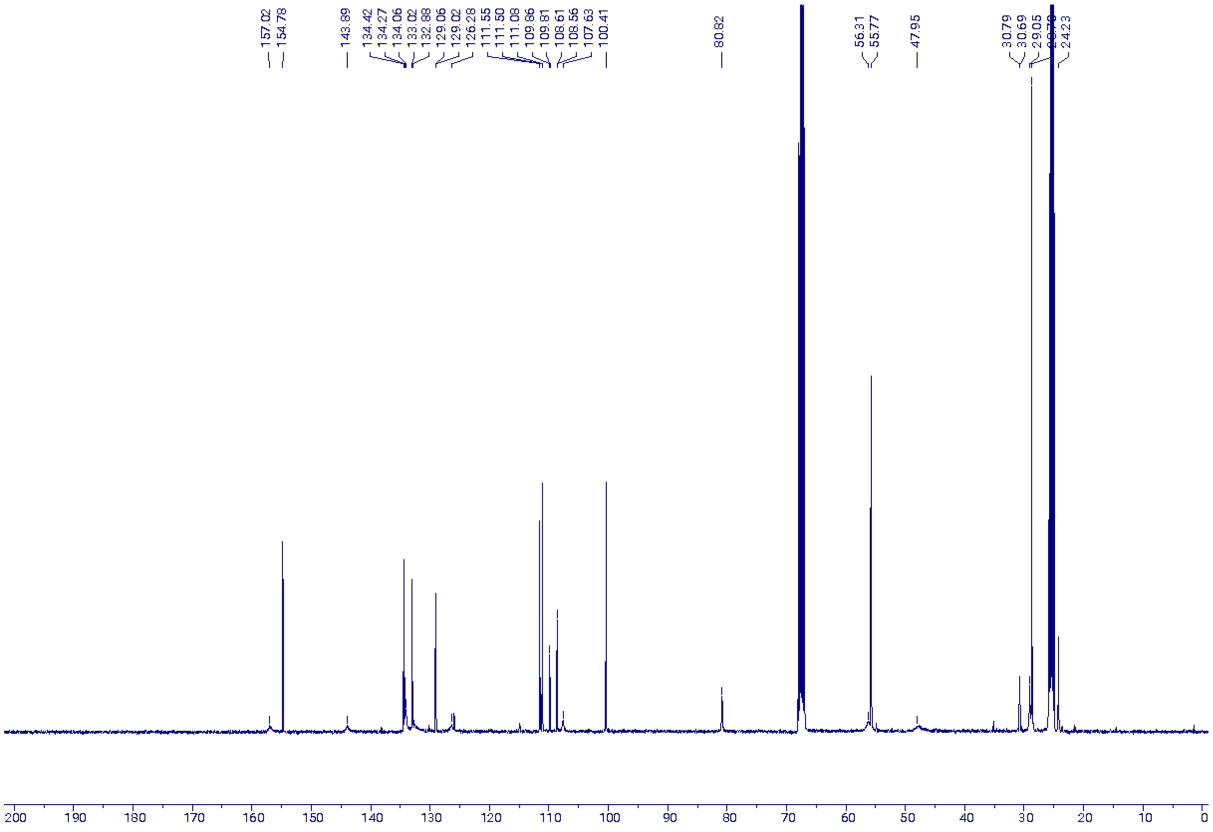

38 – ^{1}H NMR (400 MHz, $CDCl_3$, rotamers)

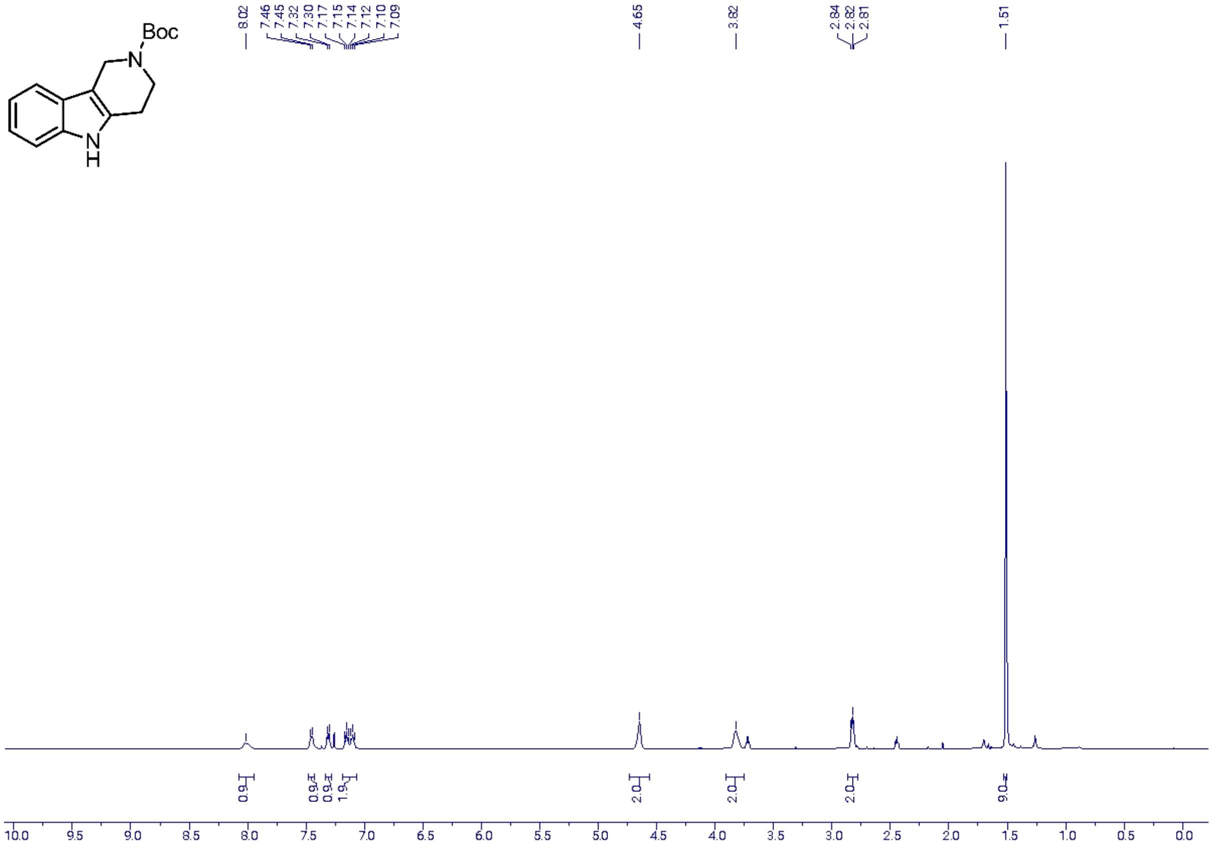

38 – ^{13}C NMR (101 MHz, $CDCl_3$)

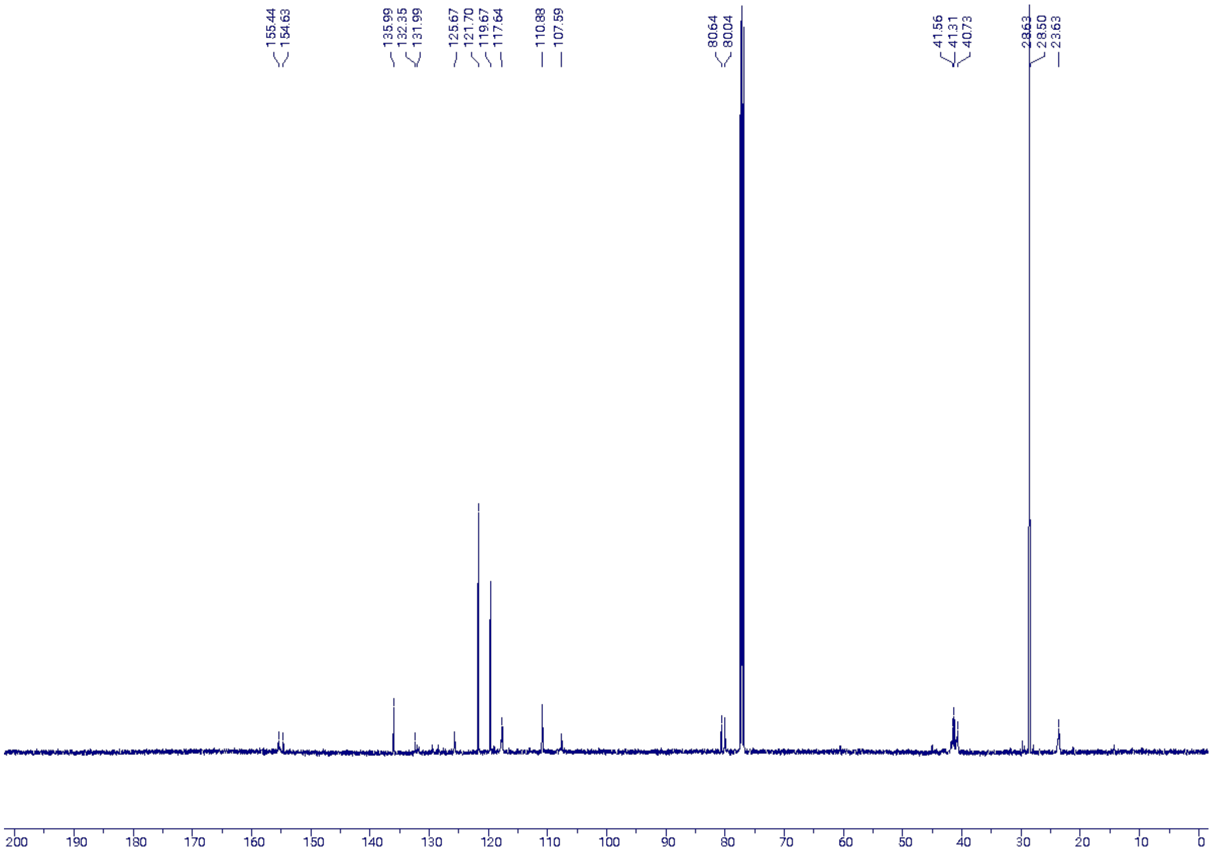

39 – ^{1}H NMR (400 MHz, $CDCl_3$)

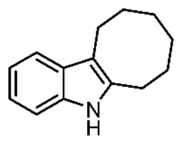

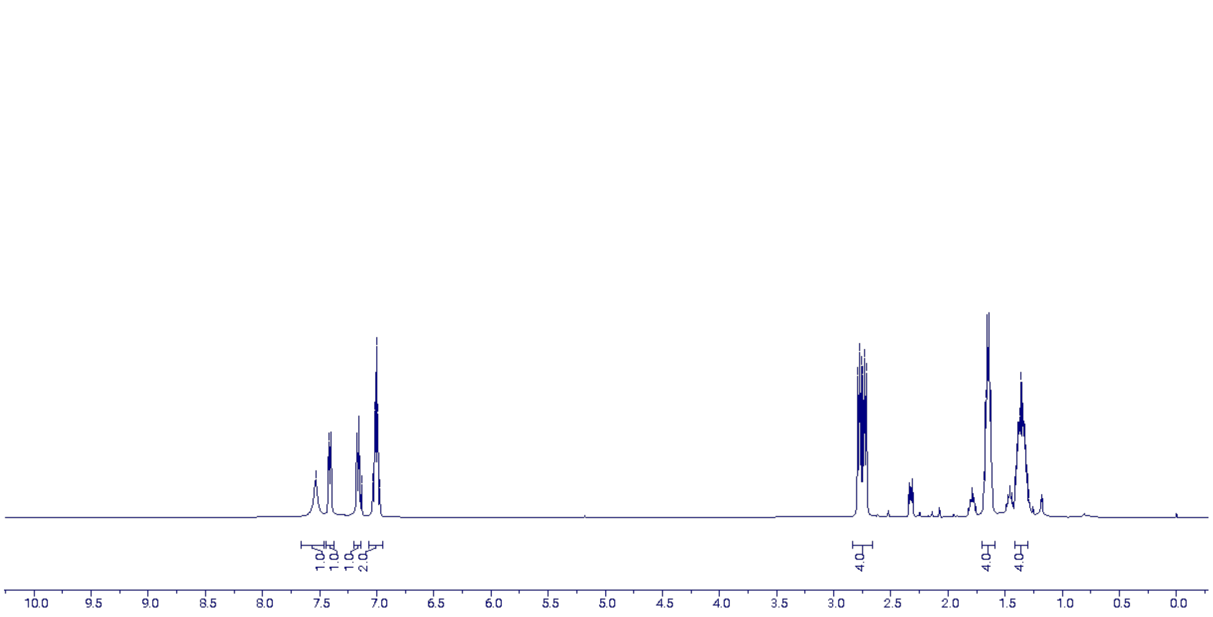

39 – ^{13}C NMR (101 MHz, $CDCl_3$)

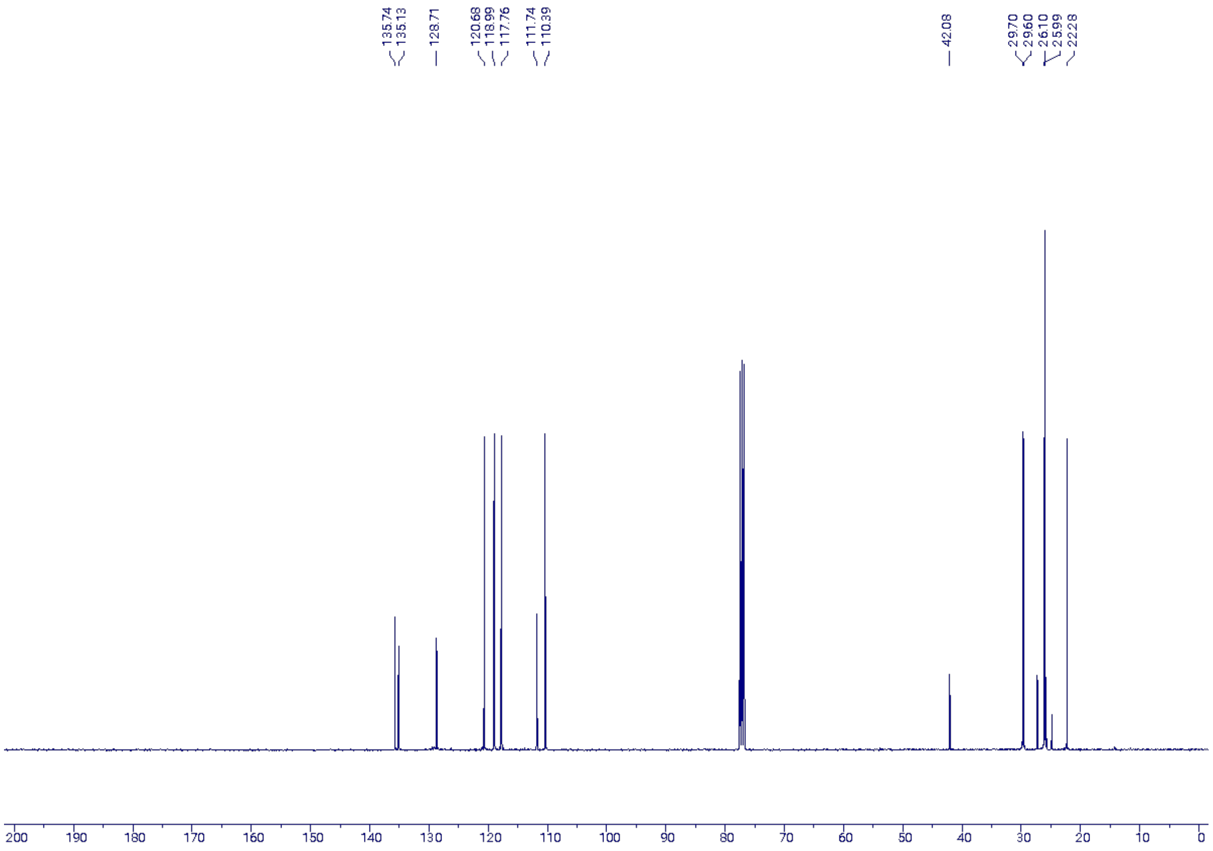

40 – ^{1}H NMR (500 MHz, $CDCl_3$)

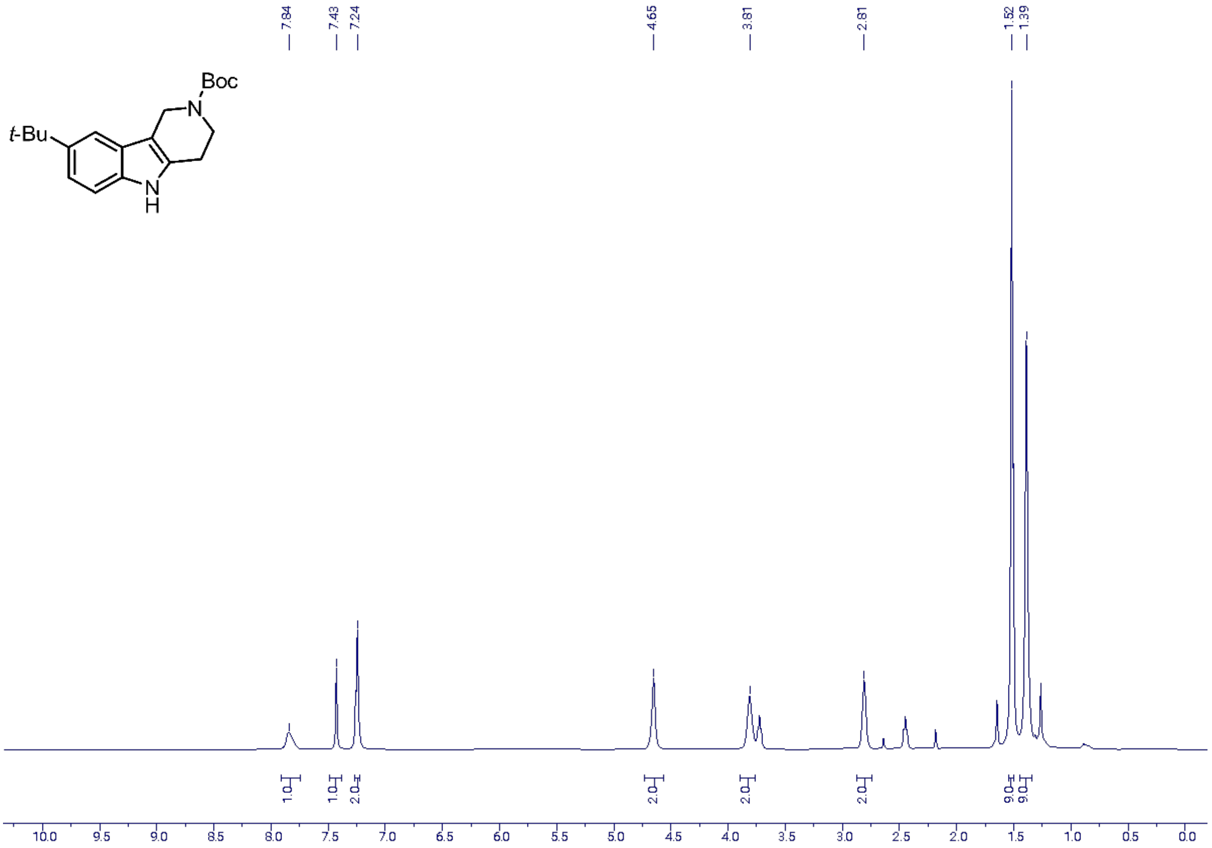

40 – ^{13}C NMR (126 MHz, $CDCl_3$, rotamers)

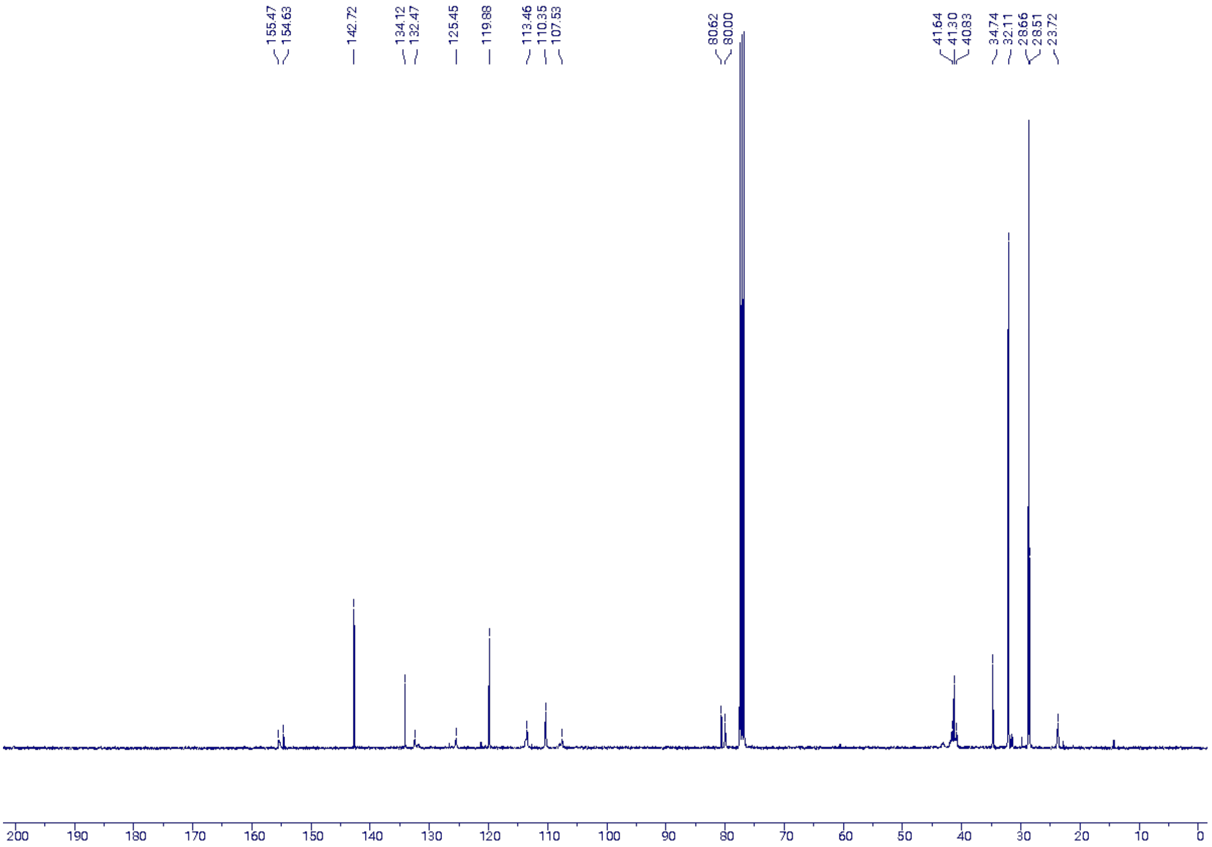

44 – ^{1}H NMR (400 MHz, CDCl$_3$)

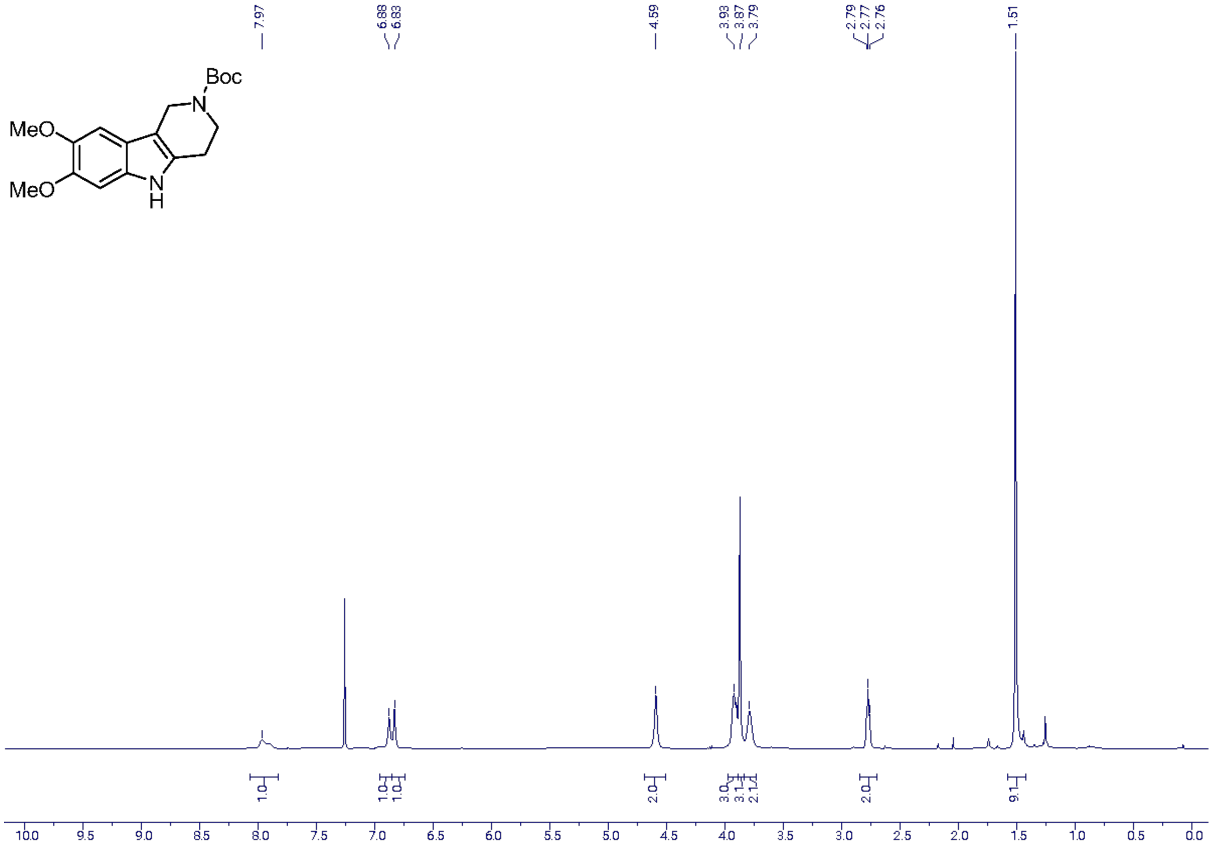

44 – ^{13}C NMR (101 MHz, CDCl$_3$)

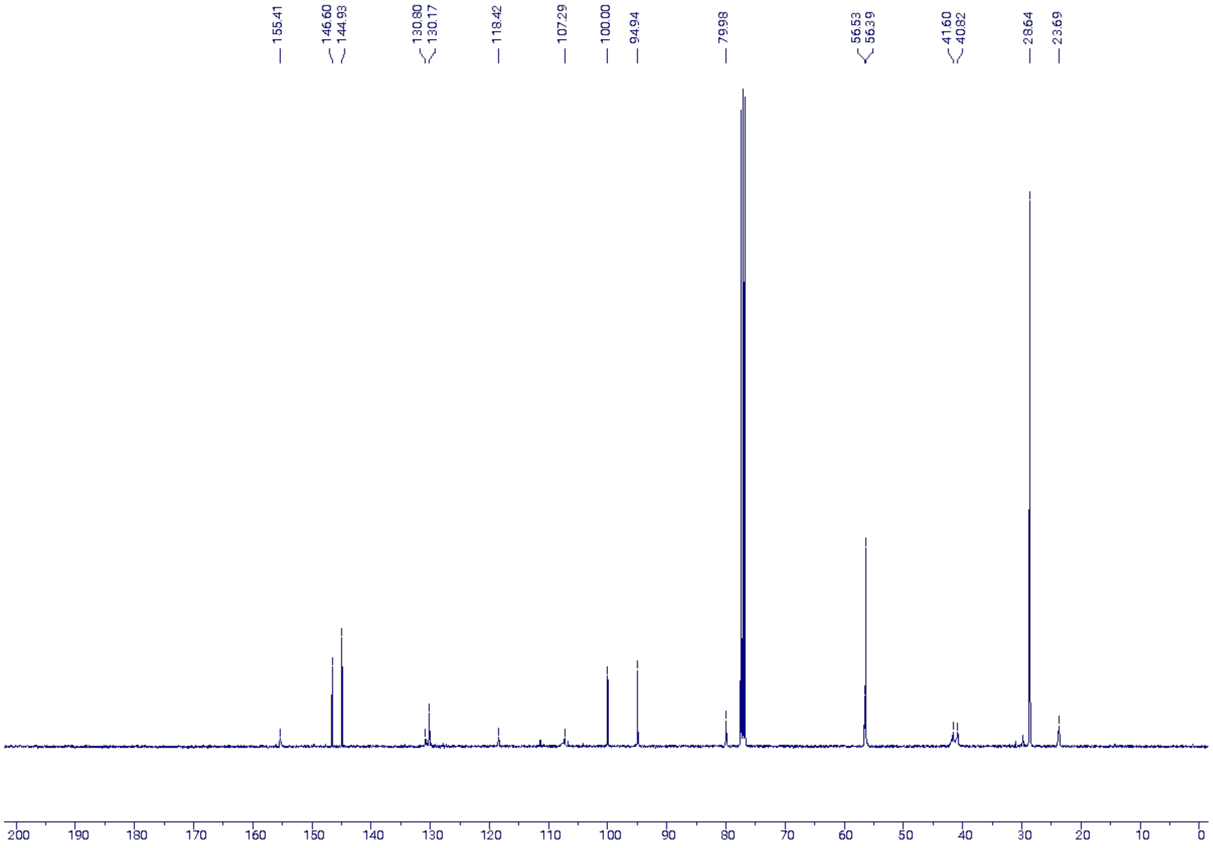

45 – ^{1}H NMR (400 MHz, $CDCl_3$)

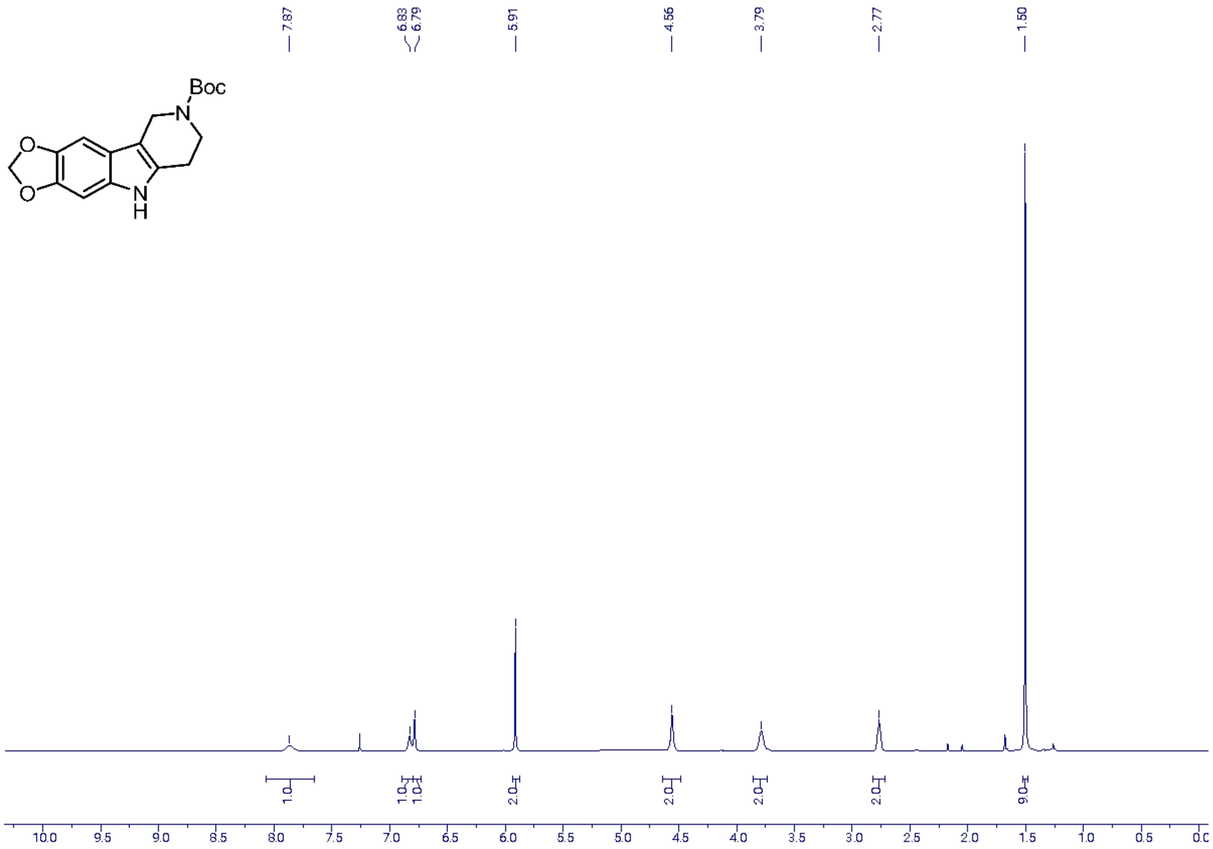

45 – ^{13}C NMR (101 MHz, $CDCl_3$)

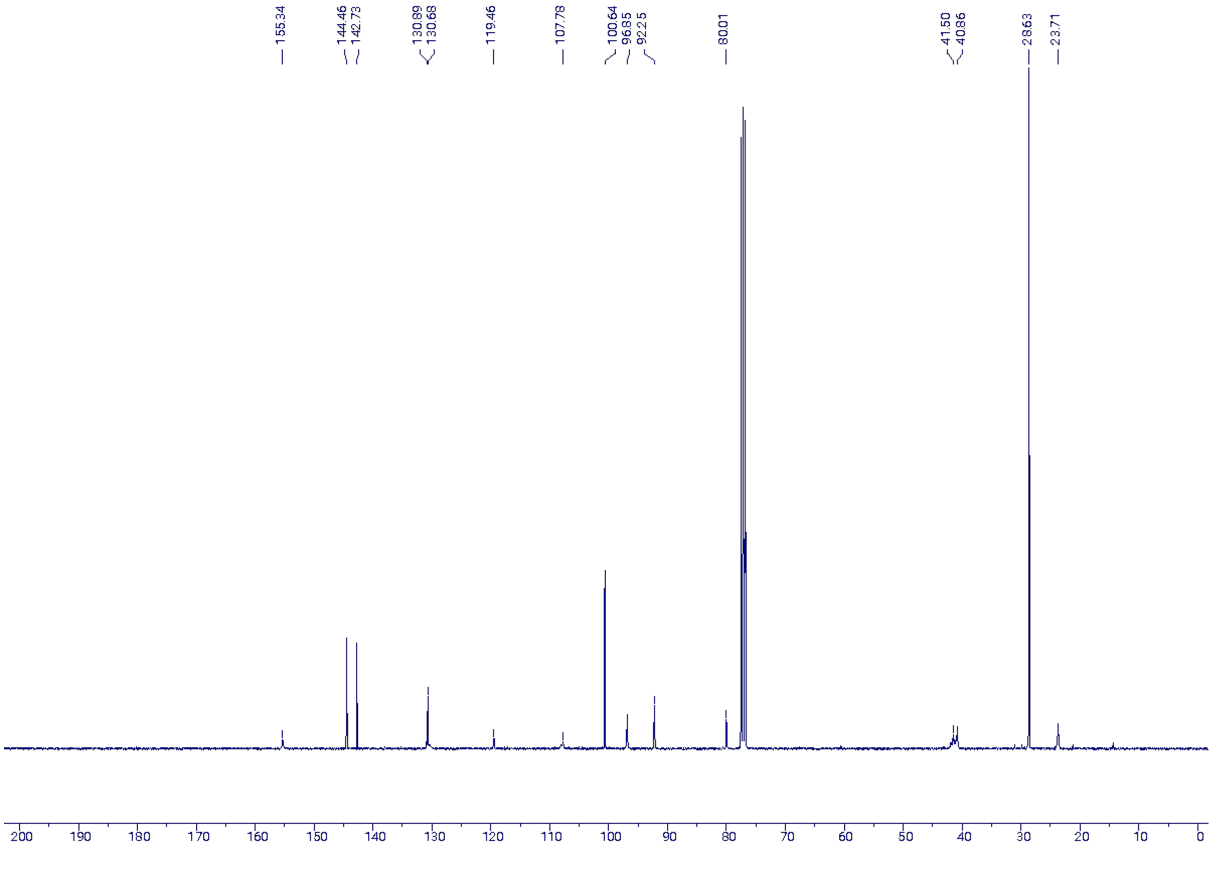

46 – ^{1}H NMR (400 MHz, CDCl3)

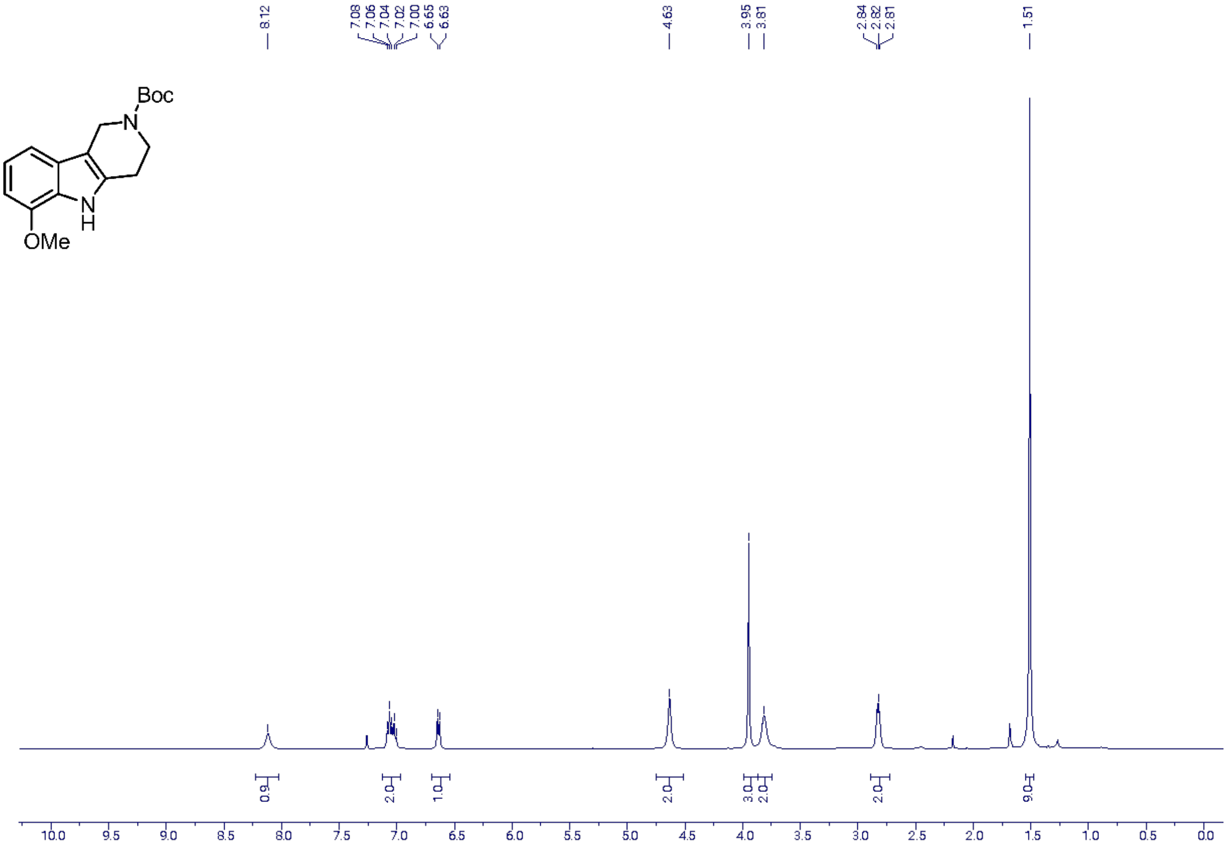

46 – ^{13}C NMR (101 MHz, CDCl3)

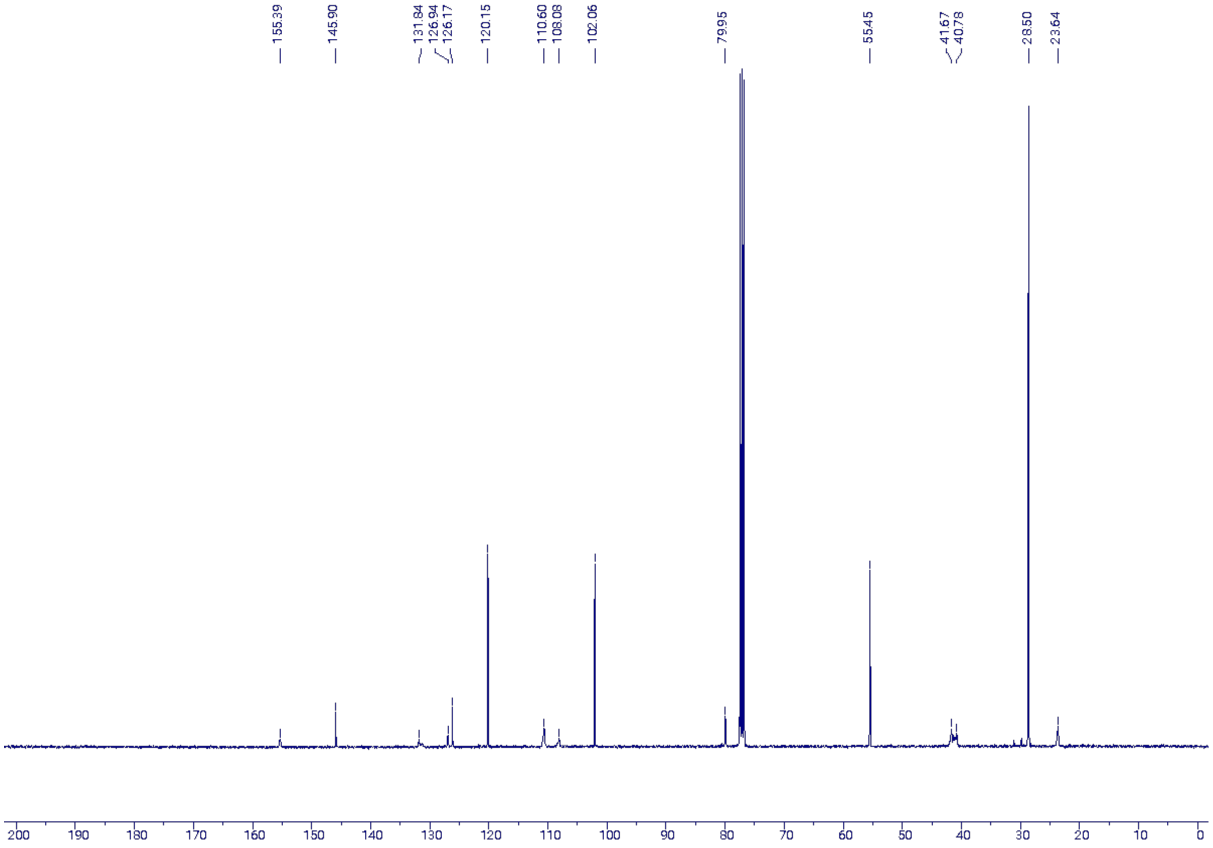

47 – ^{1}H NMR (400 MHz, $CDCl_3$)

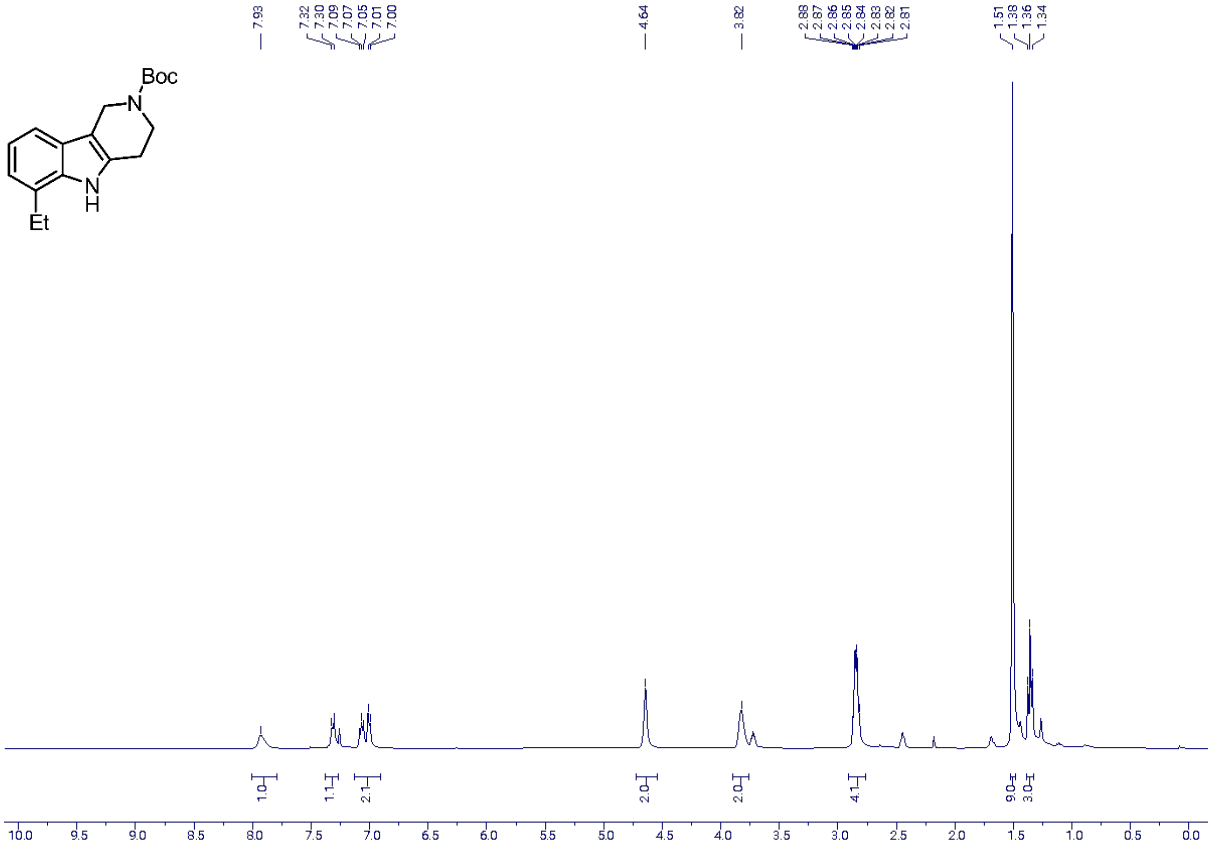

47 – ^{13}C NMR (101 MHz, $CDCl_3$)

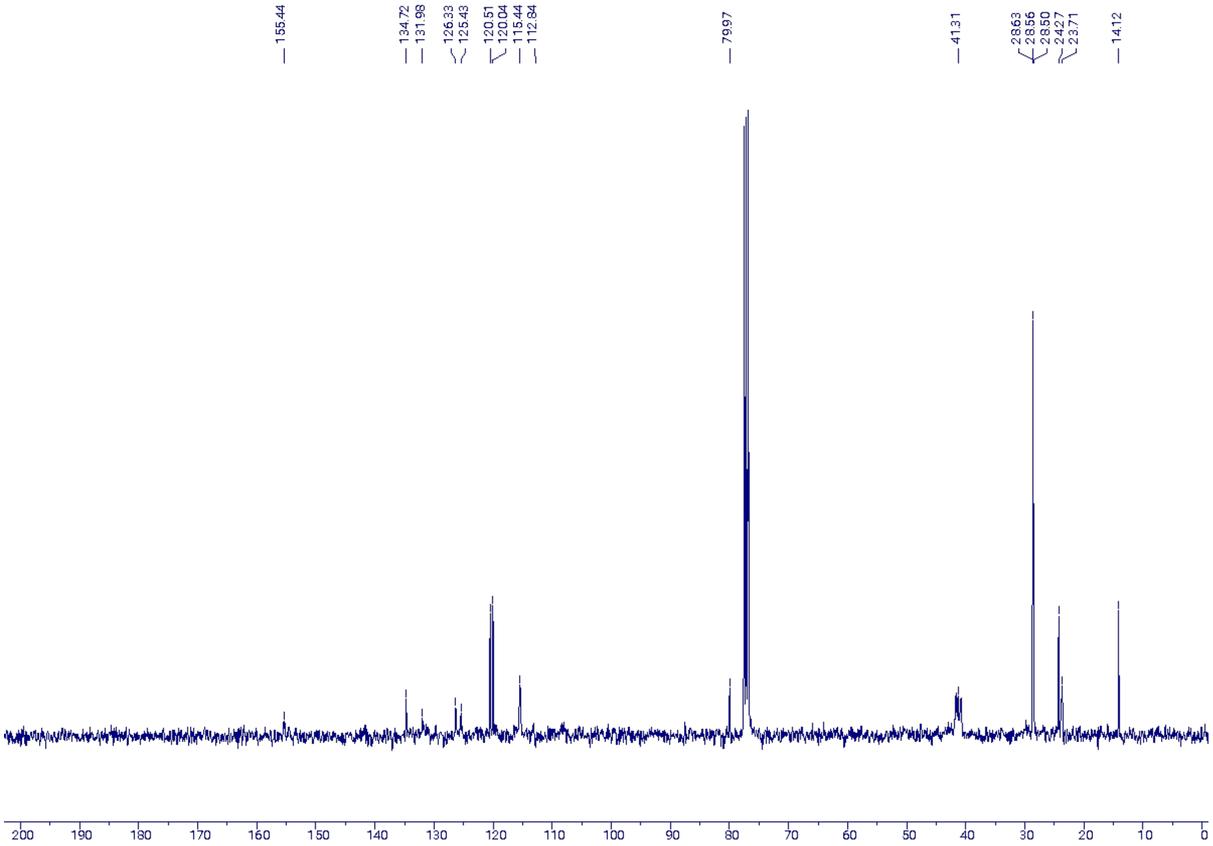

48 – ^{1}H NMR (400 MHz, $CDCl_3$)

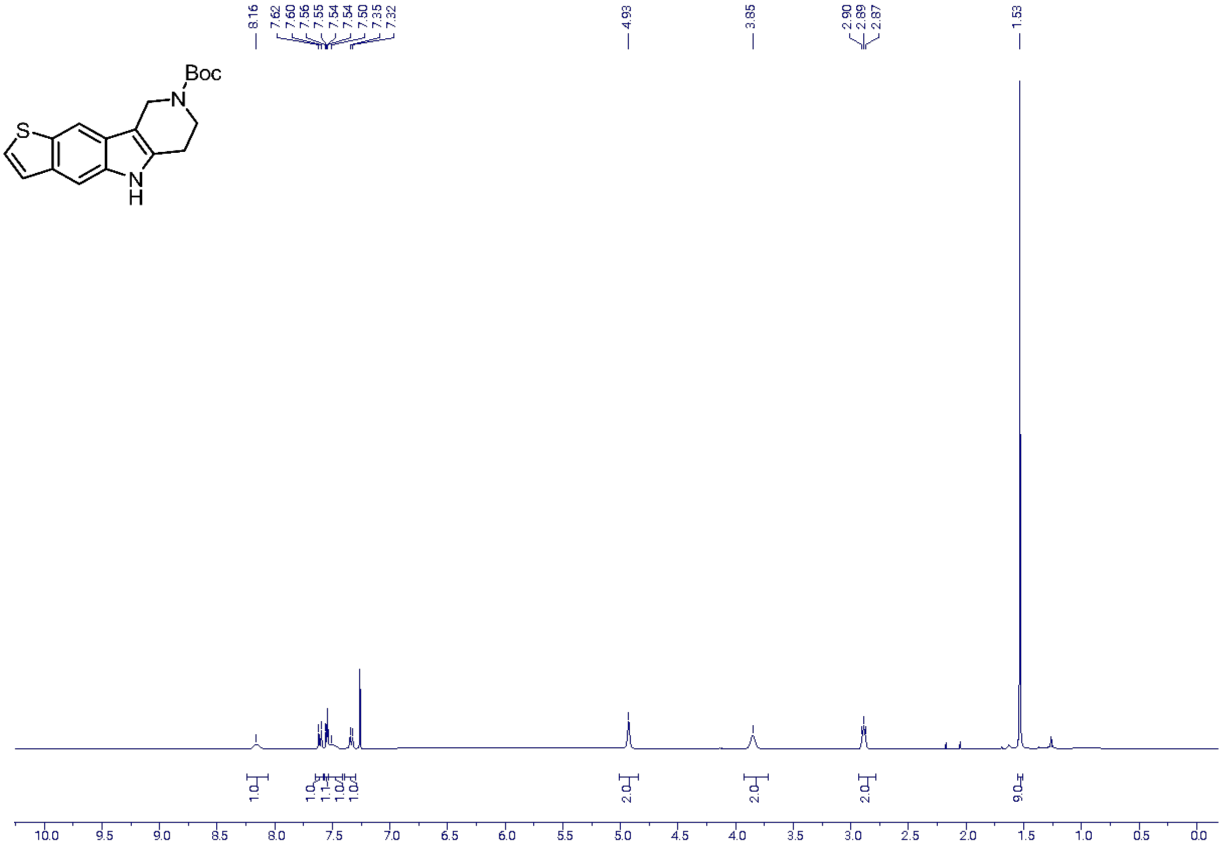

48 – ^{13}C NMR (101 MHz, $CDCl_3$)

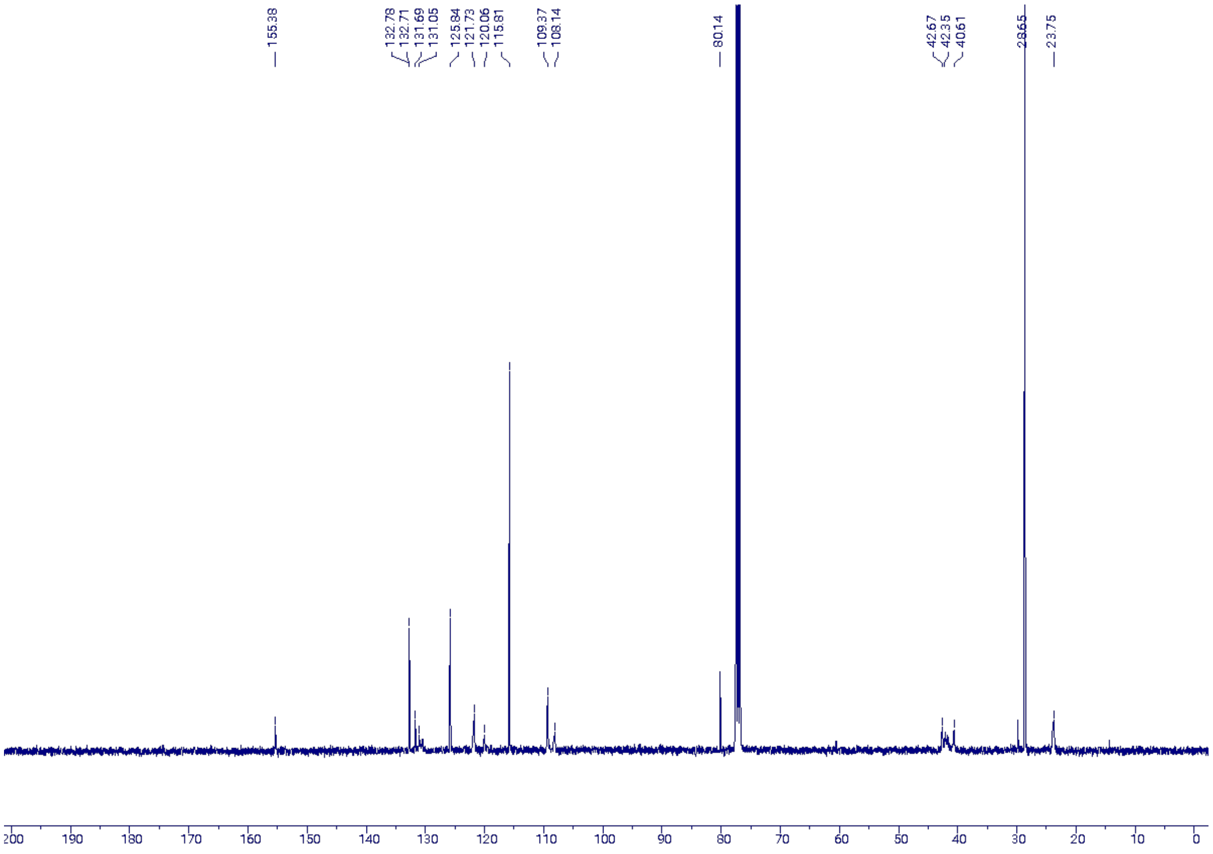

6 References

(1) Wei, H.-L.; Piou, T.; Dufour, J.; Neuville, L.; Zhu, J. Iodo-carbocyclization of electron-deficient alkenes: Synthesis of oxindoles and spirooxindoles. *Org. Lett.* **2011**, *13* (9), 2244-2247.

(2) Sivendran, N.; Belitz, F.; Sowa Prendes, D.; Manu Martínez, Á.; Schmid, R.; Gooßen, L. J. Photochemical Sandmeyer-type Halogenation of Arenediazonium Salts. *Chem. Eur. J.* **2022**, *28* (9), e202103669.

(3) Schotten, C.; Leprevost, S. K.; Yong, L. M.; Hughes, C. E.; Harris, K. D.; Browne, D. L. Comparison of the thermal stabilities of diazonium salts and their corresponding triazenes. *Org. Process Res. Dev.* **2020**, *24* (10), 2336-2341.

(4) Sherborne, G. J.; Gevondian, A. G.; Funes-Ardoiz, I.; Dahiya, A.; Fricke, C.; Schoenebeck, F. Modular and Selective Arylation of Aryl Germanes (C−$GeEt_3$) over C−Bpin, C−SiR_3 and Halogens Enabled by Light-Activated Gold Catalysis. *Angew. Chem. Int. Ed.* **2020**, *59* (36), 15543-15548.

(5) Shaaban, S.; Jolit, A.; Petkova, D.; Maulide, N. A family of low molecular-weight, organic catalysts for reductive C–C bond formation. *Chem. Commun.* **2015**, *51* (73), 13902-13905.

(6) Liu, A.-D.; Wang, Z.-L.; Liu, L.; Cheng, L. Aqueous and Visible-Light-Promoted C–H (Hetero) arylation of Uracil Derivatives with Diazoniums. *J. Org. Chem.* **2021**, *86* (23), 16434-16447.

(7) Çeken, B.; Kízíl, M. Synthesis and DNA-cleaving activity of a series of substituted arenediazonium ions. *Russ. J. Bioorganic Chem.* **2008**, *34* (4), 488-498.

(8) Zeng, C.; Shen, G.; Yang, F.; Chen, J.; Zhang, X.; Gu, C.; Zhou, Y.; Fan, B. Rhodium-Catalyzed Generation of Anhydrous Hydrogen Iodide: An Effective Method for the Preparation of Iodoalkanes. *Org. Lett.* **2018**, *20* (21), 6859-6862.

(9) Górski, B.; Barthelemy, A.-L.; Douglas, J. J.; Juliá, F.; Leonori, D. Copper-catalysed amination of alkyl iodides enabled by halogen-atom transfer. *Nat. Catal.* **2021**, *4* (7), 623-630.

(10) Moriya, K.; Knochel, P. Diastereoconvergent Negishi cross-coupling using functionalized cyclohexylzinc reagents. *Org. Lett.* **2014**, *16* (3), 924-927.

(11) Zhang, Z.; Górski, B.; Leonori, D. Merging Halogen-Atom Transfer (XAT) and Copper Catalysis for the Modular Suzuki–Miyaura-Type Cross-Coupling of Alkyl Iodides and Organoborons. *J. Am. Chem. Soc.* **2022**, *144* (4), 1986-1992.

(12) Caiger, L.; Sinton, C.; Constantin, T.; Douglas, J. J.; Sheikh, N. S.; Juliá, F.; Leonori, D. Radical hydroxymethylation of alkyl iodides using formaldehyde as a C1 synthon. *Chem. Sci.* **2021**, *12* (31), 10448-10454.

(13) Press, N. J.; Taylor, R. J.; Fullerton, J. D.; Tranter, P.; McCarthy, C.; Keller, T. H.; Arnold, N.; Beer, D.; Brown, L.; Cheung, R. Solubility-driven optimization of phosphodiesterase-4 inhibitors leading to a clinical candidate. *J. Med. Chem.* **2012**, *55* (17), 7472-7479.

(14) Nugent, J.; Arroniz, C.; Shire, B. R.; Sterling, A. J.; Pickford, H. D.; Wong, M. L.; Mansfield, S. J.; Caputo, D. F.; Owen, B.; Mousseau, J. J.; Duarte, F.; Anderson, E. A. A general route to bicyclo[1.1.1]pentanes through photoredox catalysis. *ACS Catal.* **2019**, *9* (10), 9568-9574.

(15) Gore, S.; Baskaran, S.; König, B. Fischer indole synthesis in low melting mixtures. *Org. Lett.* **2012**, *14* (17), 4568-4571.

(16) Sosic, I.; Anderluh, M.; Sova, M.; Gobec, M.; Mlinarič Raščan, I.; Derouaux, A.; Amoroso, A.; Terrak, M.; Breukink, E.; Gobec, S. Structure–activity relationships of novel tryptamine-based inhibitors of bacterial transglycosylase. *J. Med. Chem.* **2015**, *58* (24), 9712-9721.

(17) Zhang, J.; Yin, Z.; Leonard, P.; Wu, J.; Sioson, K.; Liu, C.; Lapo, R.; Zheng, S. A variation of the Fischer indolization involving condensation of quinone monoketals and aliphatic hydrazines. *Angew. Chem.* **2013**, *125* (6), 1797-1801.

(18) Li, Y.; Yan, T.; Junge, K.; Beller, M. Catalytic Methylation of C–H Bonds Using CO_2 and H_2. *Angew. Chem. Int. Ed.* **2014**, *53* (39), 10476-10480.

(19) Peña-López, M.; Neumann, H.; Beller, M. Ruthenium-Catalyzed Synthesis of Indoles from Anilines and Epoxides. *Chem. Eur. J.* **2014**, *20* (7), 1818-1824.

(20) Fretz, H.; Valdenaire, A.; Pothier, J.; Hilpert, K.; Gnerre, C.; Peter, O.; Leroy, X.; Riederer, M. A. Identification of 2-(2-(1-naphthoyl)-8-fluoro-3,4-dihydro-1*H*-pyrido[4,3-*b*] indol-5(2*H*)-yl) acetic acid (setipiprant/ACT-129968), a potent, selective, and orally bioavailable chemoattractant receptor-homologous molecule expressed on Th2 cells (CRTh2) antagonist. *J. Med. Chem.* **2013**, *56* (12), 4899-4911.

(21) Ye, L.; Tian, Y.; Li, Z.; Zhang, J.; Wu, S. Design and Synthesis of Some Novel 2,3,4,5-Tetrahydro-1*H*-pyrido[4,3-*b*] indoles as Potential c-Met Inhibitors. *Helv. Chim. Acta* **2012**, *95* (2), 320-326.
